# Structural homology screens reveal host-derived poxvirus protein families impacting inflammasome activity

**DOI:** 10.1016/j.celrep.2023.112878

**Published:** 2023-07-25

**Authors:** Ian N. Boys, Alex G. Johnson, Meghan R. Quinlan, Philip J. Kranzusch, Nels C. Elde

**Affiliations:** 1 Department of Human Genetics, University of Utah, Salt Lake City, UT 84112, USA; 2 Department of Microbiology, Harvard Medical School, Boston, MA 02115, USA; 3 Department of Cancer Immunology and Virology, Dana-Farber Cancer Institute, Boston, MA 02115, USA; 4 Howard Hughes Medical Institute, Chevy Chase, MD 20815, USA; 5 Lead contact

## Abstract

Viruses acquire host genes via horizontal transfer and can express them to manipulate host biology during infections. Some homologs retain sequence identity, but evolutionary divergence can obscure host origins. We use structural modeling to compare vaccinia virus proteins with metazoan proteomes. We identify vaccinia *A47L* as a homolog of gasdermins, the executioners of pyroptosis. An X-ray crystal structure of A47 confirms this homology, and cell-based assays reveal that A47 interferes with caspase function. We also identify vaccinia *C1L* as the product of a cryptic gene fusion event coupling a Bcl-2-related fold with a pyrin domain. C1 associates with components of the inflammasome, a cytosolic innate immune sensor involved in pyroptosis, yet paradoxically enhances inflammasome activity, suggesting differential modulation during infections. Our findings demonstrate the increasing power of structural homology screens to reveal proteins with unique combinations of domains that viruses capture from host genes and combine in unique ways.

## In brief

Viral homologs of host proteins can diverge beyond recognition by sequence comparison. Boys et al. use AlphaFold-based structural comparisons to identify homology between virus and host proteins. A47, a viral homolog of gasdermins, interferes with inflammasome activity, while C1, a pyrin-Bcl-2 fusion, is pro-inflammatory.

## Graphical Abstract

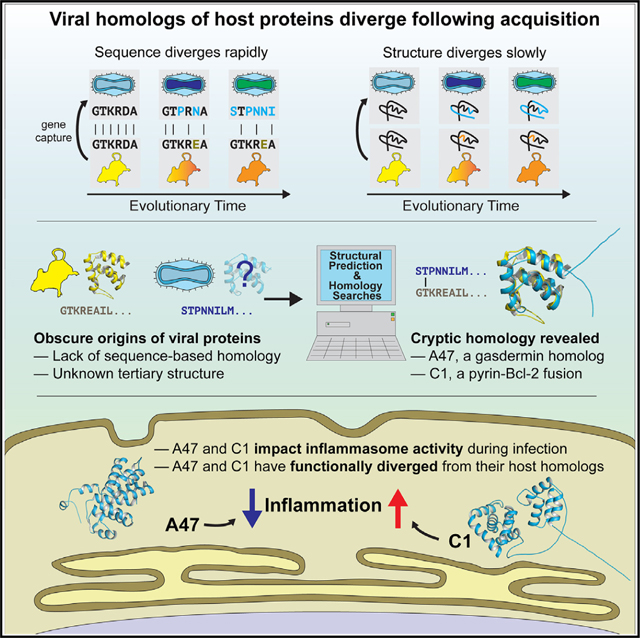

## INTRODUCTION

DNA viruses are particularly prominent “gene hunters” that capture a variety of host genes and express them in ways that alter host biology to promote viral replication.^1,2^ While the mechanisms facilitating gene capture are beginning to become clear,^3,4^ the identity and function of many host-derived viral genes remain unknown. In some cases, sequence-based comparisons reveal a clear identity between cellular and viral genes, and many crucial discoveries in cell biology and virology have originated from such comparisons.^5^ However, in many instances, rapid evolution and sequence divergence obscure homology with host genes.

While protein sequences diverge over time, critical structural elements are often preserved. Structural comparison is thus useful for inferring the origins and functions of viral proteins. For example, previous studies used structural homology searches to uncover hidden homology of viral proteins with receptor ligands,^6^ and other instances of mimicry have been proposed in homology searches.^7^ Such screens, however, are hindered by the relative dearth of structural data for viral proteins. Of the over 5,000,000 viral proteins in the UniProt database, fewer than 4,000 have associated experimental structural data (UniProt, January 2023). Recent advances in structural modeling such as those implemented in AlphaFold2^8^ have the potential to bridge this gap, and the utility of this breakthrough in identifying cryptic homology is already being appreciated. For example, *ab initio* modeling has been used to broadly identify pathogen mimics of host proteins,^9^ to identify evolutionary connections among pathogen effectors,^10^ to expand an understanding of immunoglobulin gene family evolution,^11^ and to provide insight into the distant cellular origins of structural proteins found in viruses.^12^ In this study, we use AlphaFold to enable searches for hidden homology in viral proteomes and inform the mechanistic study of host-pathogen interfaces.

We screened the vaccinia virus proteome for cryptic homology with metazoan proteins. From these comparisons, we identified and characterized two vaccinia proteins that influence host immunity. Vaccinia virus A47, encoded by the gene *A47L*, is predicted to share extensive structural conservation with gasdermin proteins, which we confirmed by determining a crystal structure of A47. A second gene, *C1L*, encodes a unique pyrin-Bcl-2 domain fusion protein. In functional studies, we provide mechanistic insight into the ability of A47 and C1 to modulate inflammasome-based immune responses.

Inflammasomes, and their ability to promote pyroptotic cell death, are important components of the host response to many viruses,^13^ including poxviruses.^14^ Previous studies have shown that poxviruses encode multiple inflammasome inhibitors,^15^ and by identifying two additional poxvirus effectors that modify inflammasome-mediated host defenses, our study broadens our understanding of how crucial the poxvirus-inflammasome interface is during infection. Importantly, these findings highlight the emerging power of structural modeling to detect cryptic homology among proteins and reveal new mechanisms for virus manipulation of host biology.

## RESULTS

### Model-based homology screens uncover relationships between vaccinia and metazoan proteins

**Figure 1.**
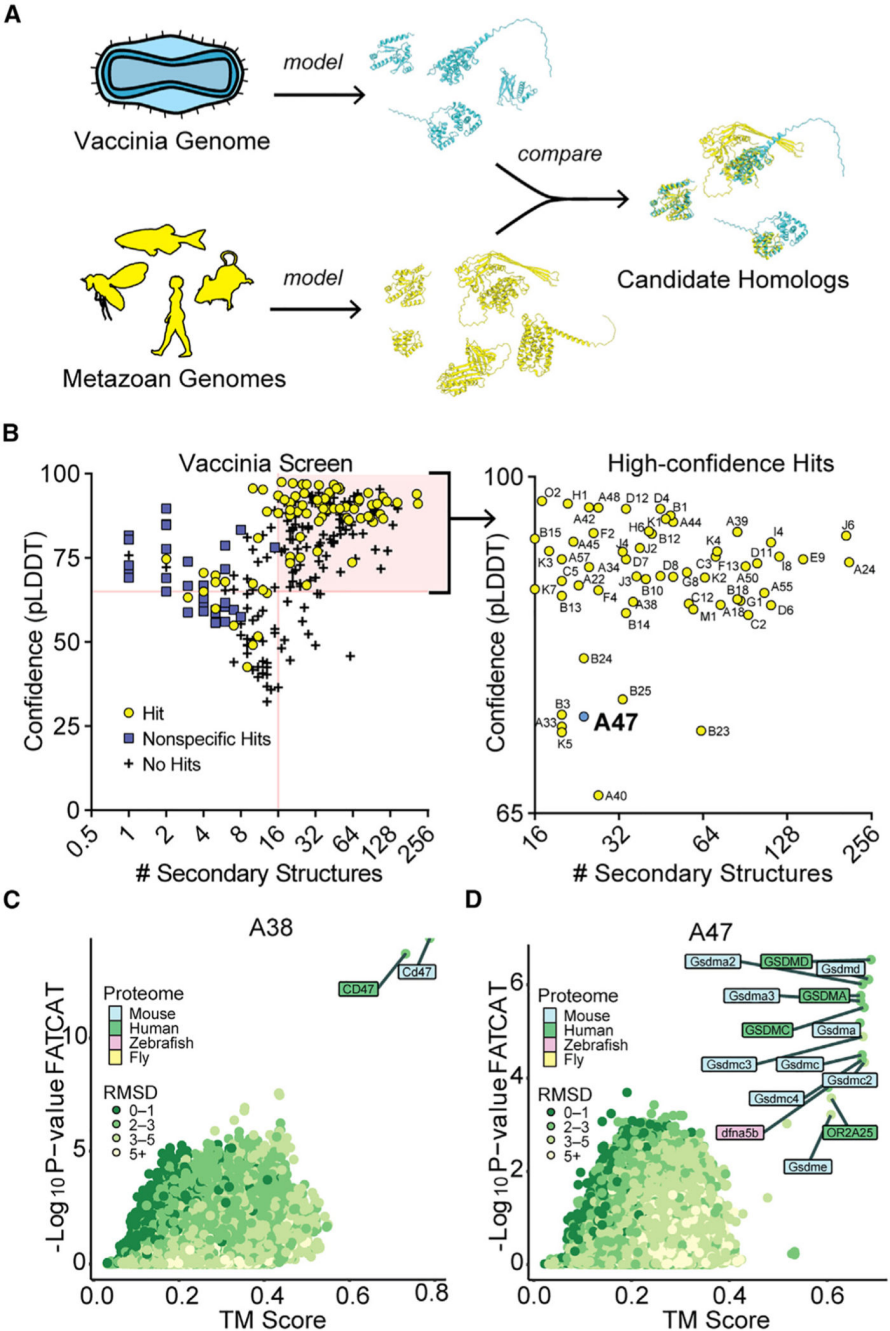
Structure-based homology screens reveal candidate homologs of vaccinia proteins (A) Homology screening pipeline. See STAR Methods for details. (B) Screen results. Right: high-confidence hits (those with model confidence [pLDDT] >65 and number of secondary structures >15 for which FATCAT and TM-align converged) are indicated. (C) FATCAT and TM-align results for A38, the poxvirus CD47 homolog. (D) FATCAT and TM-align results for A47.

We performed a structural homology screen for vaccinia virus, a model virus and vaccine strain related to mpox virus and the eradicated variola virus, the causative agent of smallpox. Vaccinia virus encodes over 200 proteins, many of which originated as host genes that were captured by horizontal transfer. While links between vaccinia genes and some host genes are evident from sequence identity, the origins of much of the viral proteome remain unclear.^16^ We modeled the vaccinia virus proteome using AlphaFold2 and performed homology searches using two orthogonal approaches. First, we used TM-align,^17^ a sensitive homology search tool that has been widely adopted for structural homology screens. We also used FATCAT,^18^ an alignment tool that permits rotations at flexible regions in structural alignments to enable searches with relaxed stringency where models may have ambiguities. We modeled all 257 predicted open reading frames (ORFs) found in the vaccinia virus strain Copenhagen and performed homology searches using AlphaFold-modeled proteomes of representative metazoans (Figure 1A).

Focusing on proteins with homology revealed by both TM-align (TM score greater than 0.6)^19^ and FATCAT (p value cutoff of 0.01), we observed that small proteins (~100 amino acids or fewer) had a high proportion of nonspecific hits in our homology searches. In such instances, TM-align and FATCAT searches either produced divergent results or identified large groups of unrelated proteins as hits (Figure S1A). Seemingly nonhomologous proteins often have similar secondary structural elements,^20^ and while the evolutionary relationships among such features are worth considering, it is difficult to distinguish between convergence or descent from a shared sequence. Many proteins with ambiguous homology were short, comprising few secondary structure elements. For example, A orf I, a 73-residue vaccinia protein, has 6 predicted secondary structural features. Homology searches identified many putative hits (Figure S1B), the strongest being p53-inducible protein 11. However, a comparison of the modeled structures of these proteins (Figure S1C) revealed that A orf I only matches a small portion of the top hit. In addition, phylogenetic analyses (Figure S1D) failed to resolve which of the widely divergent “hits” is biologically relevant. While such results may be informative in searches for distant origins of poxvirus proteins, this ambiguity led us to exclude these nonspecific hits from our screens.

By removing proteins with fewer than 16 secondary structural elements, we eliminated most nonspecific results (Figure S1E). Our search yielded 60 proteins for which structural homologs could be confidently predicted (Figure 1B; Table S1). We identified known homologs of host proteins, such as A38, the vaccinia homolog of CD47^21^ (Figure 1C). Other conserved folds, such as that of the helicase A18, the resolvase A22, the methyltransferase D12, and other components of the poxvirus transcriptional machinery, were identified, in line with previous studies^16^ (Table S1).

Alongside well-characterized homologs of host proteins, our screen identified vaccinia A47 protein (encoded by the gene *A47L*) as a structural homolog of the regulatory domain of gasdermins, the protein executioners of pyroptotic cell death (Figure 1D). Pyroptosis is an inflammatory form of programmed cell death that serves to limit pathogen spread and is defined by the activity of the gasdermin protein family.^22^ Gasdermins are two-lobed molecules comprising a lipophilic N-terminal domain (NTD) and an autoinhibitory C-terminal domain (CTD). During canonical inflammasome signaling, diverse microbe-associated stimuli promote the supramolecular assembly of inflammasomes, which in turn activate inflammatory caspases. Active inflammatory caspases subsequently cleave the interdomain linker of gasdermins, releasing the lipophilic NTD to oligomerize in membranes and form pores that induce lytic cell death and enable release of the cytokines interleukin-18 (IL-18) and IL-1β. Several viruses are known to antagonize inflammasome activity at multiple steps,^23^ including by targeting GSDMD (gasdermin D) for inactivating cleavage^24^ and by blocking its cleavage by caspases.^25^ However, the impact on inflammasome signaling by viral homologs of gasdermins have not been experimentally studied.

### A crystal structure of a poxvirus A47 paralog confirms homology with gasdermins

**Figure 2.**
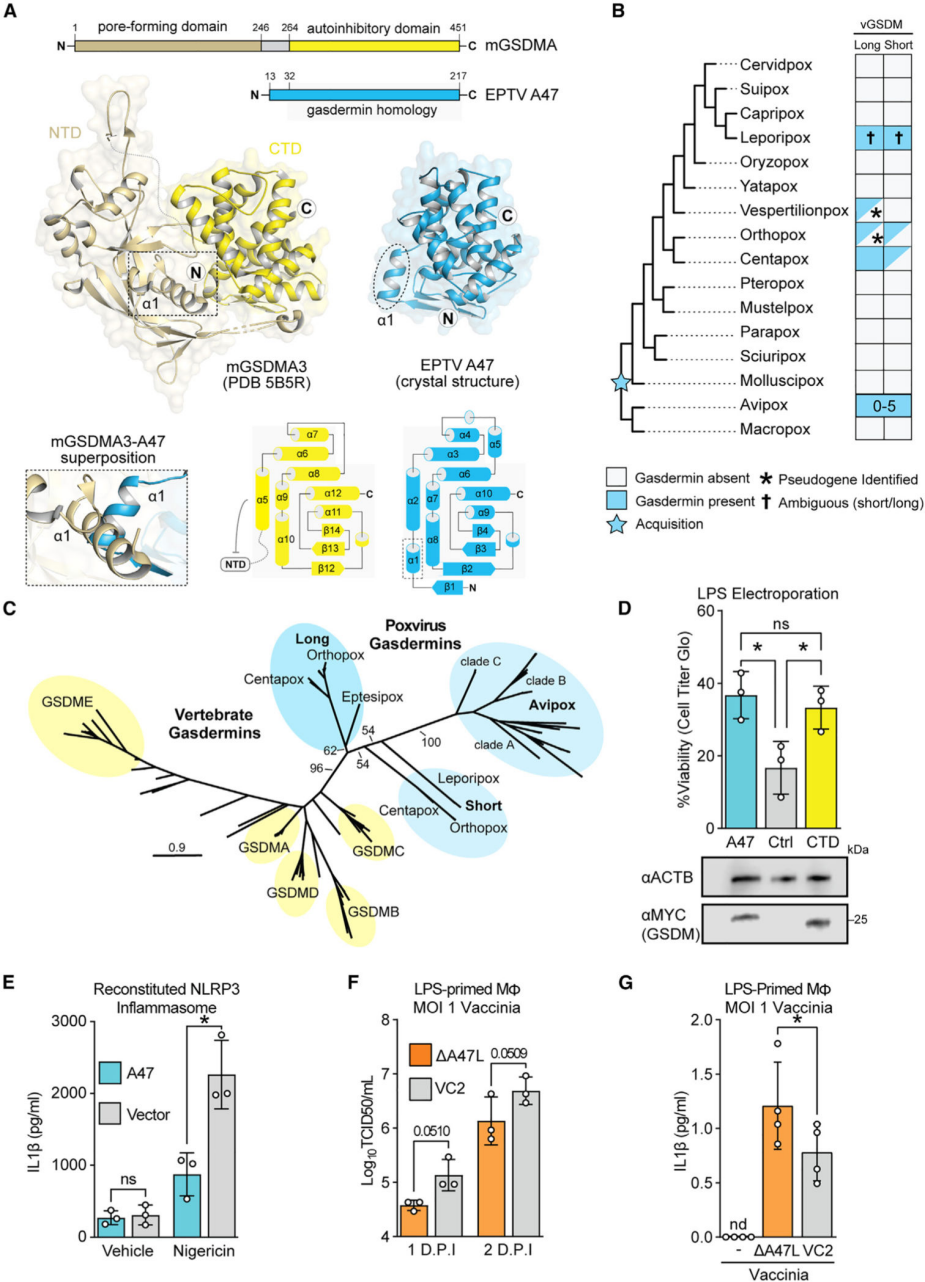
A poxvirus homolog of gasdermins interferes with inflammatory caspases (A) A crystal structure of Eptesipox virus (EPTV) gasdermin reveals homology to mammalian gasdermins. Top: domain organization of mouse GSDMA3 (mGSDMA3) and EPTV gasdermin indicating the pore-forming N-terminal domain (NTD) and the autoinhibitory C-terminal domain (CTD). Middle: crystal structures of full-length mGSDMA3 (PDB: 5B5R) and EPTV gasdermin (this study). The N and C termini are indicated with the circled letters N and C, and the dashed box and oval indicate the first α-helix of each structure. Bottom left: superposition of mGSDMA3 and EPTV gasdermin predicts a steric clash between the α1 helices of each protein. Bottom right: topology diagrams of the mGSDMA3 CTD and EPTV gasdermin indicate a conserved region of gasdermin homology. (B) Overview of probable gasdermin gain/loss events based on phylogenetic analyses in Figures 2C, S2H, and S2I. Gasdermins were identified by a combination of sequence and structure-based approaches; see STAR Methods for details. (C) Maximum likelihood tree of select vertebrate gasdermin regulatory domains and poxvirus gasdermins. Clusters of vertebrate gasdermins are labeled based on human gasdermins. Bootstrap values from 1,000 replicates are indicated for select branches. Scale: AA substitutions per site. (D) Top: HeLa cells expressing GSDMD-CTD, vaccina GSDM, or a vector control were electroporated with LPS to activate the noncanonical inflammasome. Viability as assessed by ATP levels relative to mock are indicated. n = 3 biological replicates. One-way ANOVA with Tukey’s multiple comparison test. Data are represented as mean ± SD. Bottom: alongside one replicate, levels of transfected gasdermin proteins in mock-electroporated wells were assessed by western blotting. (E) The murine NLRP3 inflammasome was reconstituted in 293T cells stably expressing *A47L* or a vector control. Following nigericin stimulation, IL-1β concentrations in supernatants were quantified by ELISA. n = 3 biological replicates. Two-way ANOVA with Šídák’s multiple comparisons test. Data are represented as mean ± SD. (F) Vaccinia replication (inoculum MOI of 1) in LPS-primed murine macrophages was measured by TCID50. n = 3 biological replicates. Two-way ANOVA with Fisher’s least significant difference (LSD) test. Data are represented as mean ± SD. ANOVA statistic for viral strain across time points: p = 0.0118. (G) LPS-primed murine macrophages were infected with wild-type (VC2) or A47-deficient vaccinia virus at an MOI of 1, and IL-1β was quantified by ELISA 1 day post-infection. n = 3 biological replicates. Paired t test. Data are represented as mean ± SD.

To definitively determine whether A47 is a virus-encoded gasdermin homolog, we sought to experimentally define its structure and tested several viral gasdermin homologs for expression and solubility in *E. coli* (see STAR Methods). We determined a crystal structural of the bat-infecting vespertilionpoxvirus eptesipoxvirus A47 ortholog at 1.46-Å resolution (Figure 2A). Eptesipox gasdermin exhibits marked structural homology with diverse metazoan gasdermin CTDs and the predicted AlphaFold model of vaccinia A47 (Figures S2A–S2D). Eptesipox gasdermin and the gasdermin CTD share the distinctive α-helical bundle fold, comprising eight α-helices and three antiparallel β-strands (Figure 2A).^26,27^ Besides the homologous regions, eptesipox gasdermin has an additional α-helix off its N termini that is not present in metazoan gasdermins (Figures 2A and S2D). Structural superposition demonstrates a potential steric clash of this helix with the gasdermin NTD, suggesting that homologs of A47 that share this structural feature, including vaccinia A47 (Figure S2E), may not directly inhibit pore formation by directly binding gasdermin NTDs and interfering with their function (Figure 2A). We also observe that some viral gasdermins do not contain an N-terminal helix with a predicted steric clash (Figure S2F), perhaps indicative of divergent functions. While viral gasdermins do not contain a caspase cleavage site, inflammatory caspases bind gasdermin regulatory domains at an exosite.^28,29^ A superposition of eptesipox gasdermin with the gasdermin D CTD from a co-crystal structure of human caspase-1 and GSDMD does not reveal any steric clashes, indicative of a potential caspase-directed role during infection (Figure S2G). These results confirm the presence of distinct viral gasdermin paralogs that are widely conserved among diverse poxviruses.

### A diverse family of poxvirus gasdermin homologs

Given predicted structural overlap between A47 and gasdermins, we performed complementary searches for viral gasdermins in poxvirus proteomes using PSI-BLAST,^30^ a pox-specific local BLAST database (see STAR Methods), and HMMER.^31^These sensitive queries revealed poxvirus gasdermins in ortho- and centapoxviruses consistent with a recent survey^16^ as well as additional hits in vespertilion-, lepori-, and avipoxviruses. These findings are consistent with the acquisition of the poxvirus gasdermin gene from an ancient host followed by extensive sequence divergence that obscured a host origin (Figure 2B).

Intriguingly, some poxvirus genomes in the mammal-infecting orthopox and centapox clades have two copies of viral gasdermin, one “long” form, shared with vaccinia virus, and another somewhat truncated “short” form. Strikingly, avipoxviruses have up to 5 viral gasdermins that cluster separately from mammal-infecting poxviruses into three distinct clades. When compared to vertebrate gasdermin regulatory domains, all viral gasdermins cluster phylogenetically (Figure 2C), indicative of a single acquisition. Additionally, we found that centapox and orthopox gasdermins are proximally located in poxvirus genomes (Figure S2H), implying that they are descended from a single acquisition and early duplication. It is unclear whether present-day leporipox gasdermins are descended from the “long” or “short” gasdermins, as they are syntenic with the “long” gasdermin in ortho- and centapoxviruses (Figure S2H) but cluster with the “short” gasdermin phylogenetically (Figure 2C). It is possible that leporipox gasdermins convergently evolved to include truncations like those in the “short” gasdermins found in other poxviruses or that additional recombination events led to a rearrangement of the genes present at this locus.

Compared with other poxviruses, the genomes of bird- and reptile-infecting avipoxviruses have undergone extensive structural rearrangements,^32^ complicating our efforts to perform synteny analyses in this poxvirus clade. Instead, we performed a detailed gain/loss analysis (Figure S2I) of the three major avipox gasdermin paralogs (Figure 2C). Strikingly, of the fourteen full-length avipoxvirus genomes we analyzed, viral gasdermins were present in all but one (crowpox), which has two degraded gasdermin pseudogenes, indicative of recent losses. Additionally, each individual clade of avipox gasdermins is preserved across multiple divergences, consistent with widespread adaptive utility to poxviruses.

The persistence of two gasdermin paralogs in several genomes in the orthopox and centapox viruses, and of all three avipox gasdermin clades in several avipoxviruses (albatrosspox, cooks petrelpox, pigeonpox, and flamingopox), further supports the idea that viral gasdermins are beneficial to poxviruses and may indicate that they serve distinct roles in poxvirus biology.

### Vaccinia A47 interferes with inflammatory caspases

To test whether A47 is an inhibitor of inflammasome activity and pyroptosis, we used an established HeLa cell-based assay^33^ to assess gasdermin D-dependent pyroptosis. In this assay, electroporated lipopolysaccharide (LPS) is sensed by the noncanonical inflammasome, promoting gasdermin D cleavage via activated caspase-4/–5, resulting in pyroptotic cell death. Importantly, expression of the regulatory domain of gasdermin D prevents LPS-induced cell death in this assay,^34^ though it has not been established whether this is via the regulatory domain serving as a “sponge” that sequesters gasdermin pore-forming domains or via its occluding the exosite on activated inflammatory caspases. We found that expression of vaccinia virus gasdermin similarly rescues cells from LPS-induced cell death (Figure 2D), consistent with a model in which poxviruses repurposed this homolog of a gasdermin regulatory domain as an inhibitor of inflammasome activity and pyroptosis.

Since our crystal structure indicates a likely steric clash between vaccinia A47 and gasdermin pore-forming domains (Figures 2A and S2E) yet does not show a steric clash between A47 and inflammatory caspases (Figure S2G), we hypothesized that A47 dampens inflammasome signaling and inhibits pyroptosis by interfering with inflammatory caspases. We used an established system^35^ to reconstitute the NLRP3 inflammasome in 293T cells which lack gasdermin D^36^ to ask whether A47 can inhibit caspase-dependent processing of pro-IL-1β. Indeed, we found that IL-1β release, and therefore caspase-1 processing of pro-IL-1β, was significantly reduced in cells expressing vaccinia A47 (Figure 2E). Supporting this conclusion, the individual components of the NLRP3 inflammasome expressed to similar levels in A47-expressing and control cells (Figure S2J). We additionally used mammalian- (Figure S2K), yeast- (Figure S2L), and bacteria-based (Figure S2M) systems to ask whether A47 could inhibit gasdermin-mediated cell death in the absence of caspase activity and found that A47 did not inhibit gasdermin D NTD-mediated killing in any system, suggesting that A47 does not act as a “sponge” for gasdermin D NTDs. Together, these results support a model in which vaccinia A47 protein interferes with the ability of inflammatory caspases to process their substrates following inflammasome activation.

We next assessed the role of A47 during vaccinia infection by generating A47L-deficient virus (Figures S2N and S2O). Consistent with previous reports,^37^ A47L-deficient vaccinia virus replicated to wild-type levels in permissive cells such as BHKs (Figure S2P). However, we found that A47L-deficient virus was attenuated in a murine macrophage line (Figure 2F; ANOVA p = 0.0118) and promoted higher levels of IL-1β release during infection (Figure 2G). Together, these findings provide complementary evidence that the ancient acquisition of a gasdermin gene by poxviruses led to evolution of viral gasdermins, such as vaccinia A47, that suppress inflammasome activity during infection. A47 adds to a collection of poxvirus proteins that interfere with inflammasomes or their downstream effectors and cytokines.^23^ Among them are soluble IL-1β^38^ and IL-18^39^ binding proteins, an NLRP1 inhibitor,^40^ and serine protease inhibitors implicated in regulating caspase activity.^41^ The diversity of means by which poxviruses target this immune pathway highlights its importance in host responses to poxvirus infections.

### Domain-specific homology searches reveal a cryptic pyrin domain-Bcl-2 fusion protein

**Figure 3.**
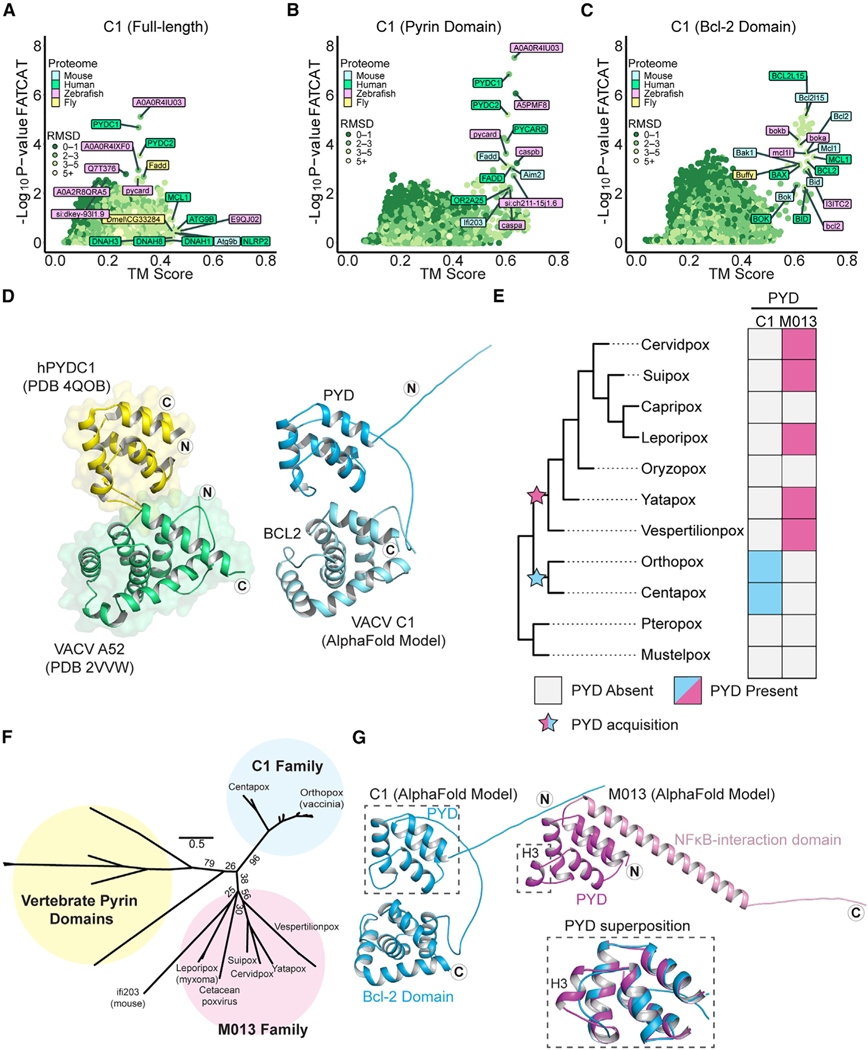
Vaccinia C1L encodes a unique pyrin-Bcl-2 fusion protein (A) FATCAT and TM-align results for full-length C1 protein. Top results for each individual search tool are labeled. (B) FATCAT and TM-align results for the N-terminal (1–107) residues of C1 protein. Only a subset of hits are labeled. (C) FATCAT and TM-align results for the C-terminal (108–224) residues of C1 protein. Only a subset of hits are labeled. (D) C1 protein is a Pyrin-Bcl-2 fusion. Left: crystal structures of human PYDC1 (PDB: 4QOB) and vaccinia virus A52 (PDB: 2VVW). Right: AlphaFold model of vaccinia C1 protein. The N and C termini are indicated with the circled letters N and C. (E) Reconstructed gain/loss tree highlighting C1 and M013 pyrin-domain-containing proteins. (F) Maximum likelihood phylogenetic tree of vertebrate pyrin domains from Figure 4B alongside the M013 and C1 families. Bootstrap values from 1,000 replicates are indicated for select branches. Scale: AA substitutions per site. (G) AlphaFold models of vaccinia virus C1 protein and myxoma virus M013 protein. The N and C termini are indicated with circled letters N and C. Inset: superposition of the C1 and M013 pyrin domains showing the predicted absence of helix 3 in C1 protein.

While analyzing host gene capture events (Figure 1B; Table S1), an interesting class of genes encoding potential domain fusions became apparent. These were instances where TM-align and FATCAT results failed to converge and where FATCAT, which allows for flexible alignments, identified homology with multiple distinct protein families. A notable example is vaccinia C1, which was previously described as a member of the poxvirus A46/Bcl-2 domain protein family.^42^ FATCAT identified limited overlap with Bcl-2 family members such as MCL1 and, at the same time, indicated robust homology with pyrin-domain-containing proteins (Figure 3A). Consistent with it being the product of a fusion event, the structural model of C1 contains two globular protein domains attached by a flexible linker, and domain-specific searches (Figures 3B and 3C) demonstrated that C1 is comprised of an N-terminal pyrin and a C-terminal Bcl-2 domain. We used DALI^43^ to search the Protein Data Bank (PDB) for solved structures similar to these two domains and found that the pyrin domain most closely resembles pyrin-domain-containing proteins such as NLRP3 and ASC, while the Bcl-2 domain is most similar to other poxvirus Bcl-2-domain-containing proteins (Figure S3A). A structural comparison of the AlphaFold model of C1 protein to hits for each domain revealed marked homology, consistent with results from our model-based homology screens (Figure 3D).

Members of the poxvirus Bcl-2 domain family have been variously implicated as immunomodulatory proteins that act through multiple mechanisms,^42^ though no immunomodulatory function has been ascribed to C1 protein. Indeed, the only prior mechanistic study of C1 protein showed that unlike many other poxvirus Bcl-2 family proteins, C1 is not antiapoptotic and does not bind or inhibit the pore-forming Bcl-2 protein BAX.^44^ Pyrin domains are protein-protein interaction domains that mediate many of the steps involved in inflammasome activation and pyroptosis. The pyrin-domain-containing protein family is comprised of the pattern recognition receptors NLRPs (NOD-like receptor proteins), the DNA sensors AIM2 and IFI16, the adapter protein ASC, and pyrin-only proteins (POPs), which serve as regulators of inflammasome signaling.^45^ Notably, no proteins containing both a pyrin and a Bcl-2 domain have been described to date, highlighting the unusual composition of this poxvirus protein.

### C1 protein is a member of a unique family of viral pyrin-domain-containing proteins

We sought to determine whether C1 protein is the result of *de novo* gene acquisition events or if it is a divergent ortholog of a known poxvirus pyrin protein, myxoma virus M013, which might have acquired a Bcl-2 domain. To date, the M013 family is the only family of pyrin-domain-containing proteins described in poxviruses. M013 regulates both nuclear factor κB (NF-κB) signaling and inflammasome activity through distinct mechanisms^46,47^ and is a major contributor to myxoma virus pathogenesis.^48^ We searched poxvirus genomes for pyrin domains using both M013 and C1 proteins as seeds for psi-BLAST^30^ and HMMER^31^ searches and only identified orthologs of C1 in clades of orthopox and centapox viruses (Figure 3E). As previously described, orthologs of M013 were found in clade II poxviruses^49^ and were absent in orthopoxviruses^50^ (Figure 3E). We also identified distantly related homologs of M013 in the bat-infecting vespertilionpoxviruses eptesipox virus and hypsugopox virus, as well as in a cetacean poxvirus (Figure 3E; Table S2).

Phylogenetic analysis of poxvirus pyrin-domain-containing proteins and select vertebrate pyrin domains supports a model in which C1 and M013 resulted from independent gene acquisition events (Figure 3F). Furthermore, synteny analysis revealed that the genes encoding C1 and M013 are located in distinct regions of poxvirus genomes, also consistent with independent acquisitions of genes encoding these pyrin domains (Figure S3B). Finally, superimposing models of C1 and M013 revealed structural differences between the two proteins (Figure 3G), which are also apparent from comparisons of amino acid sequences (Figure S3C). These data suggest that poxviruses acquired pyrin domains independently on at least two occasions, highlighting the potential of viral proteins containing pyrin domains to impact poxvirus infections.

### Vaccinia C1 protein promotes ASC-dependent inflammasome activation

**Figure 4.**
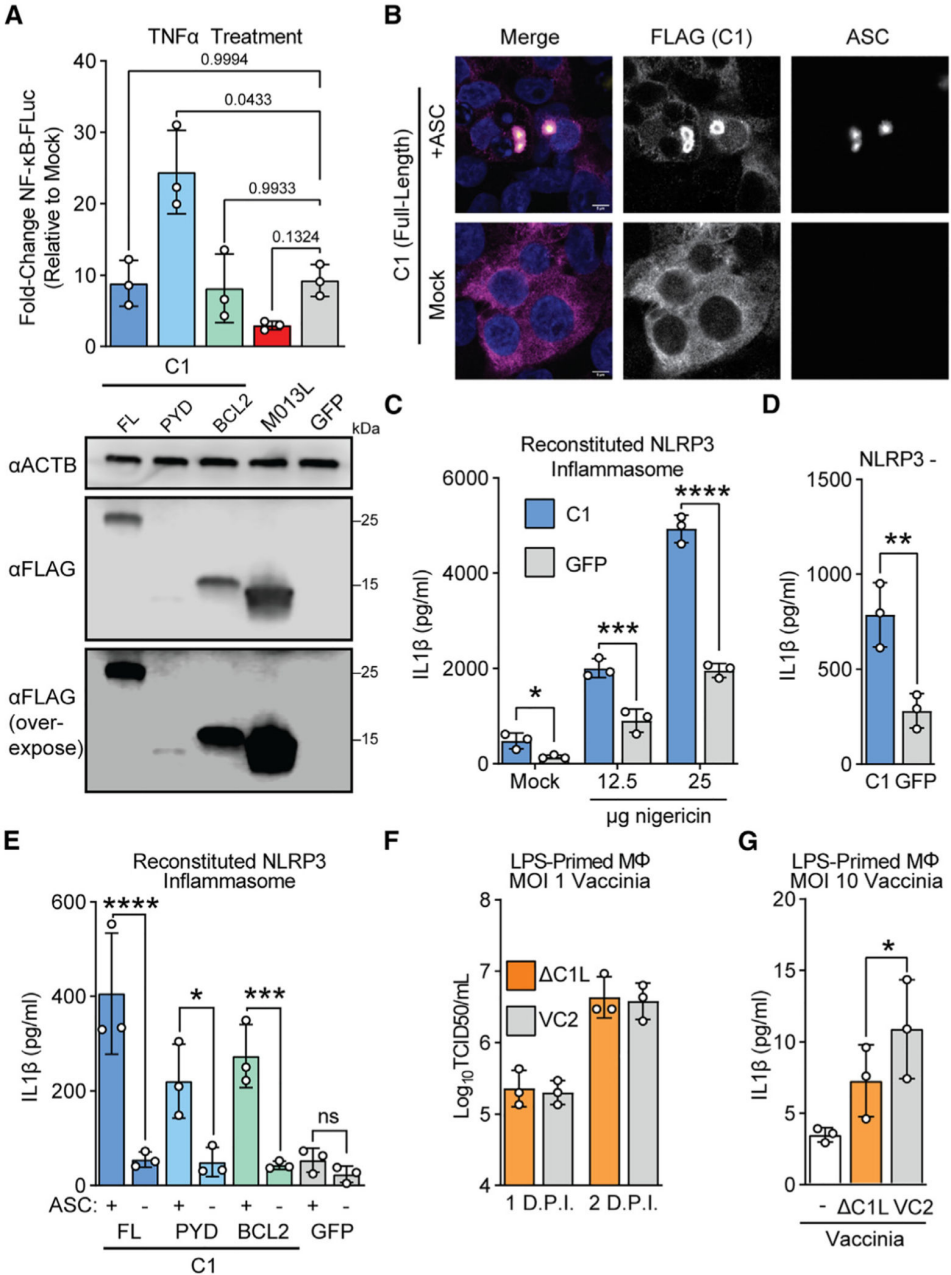
C1 protein promotes ASC-dependent inflammasome activation (A) Top: 293T cells were co-transfected with the indicated plasmids, a plasmid encoding firefly luciferase driven by an NF-κB promoter, and a constitutive Renilla luciferase plasmid as a transfection control. 24 h post-transfection, cells were treated with 1 ng/mL TNF-α, and promoter activity was assessed by luciferase assay 6 h post-transfection. n = 3 biological replicates. Repeated measures (RM) one-way ANOVA with Dunnett’s correction. Data are represented as mean ± SD. Bottom: in parallel, cells were transfected with equal amounts of the indicated plasmids, and protein expression was determined by western blotting. (B) 293T cells were co-transfected with a plasmid encoding FLAG-C1 and either ASC-GFP^51^ or an empty vector. 24 h post-transfection, cells were fixed and stained for FLAG (C1) and subsequently imaged. Representative image from n = 3 independent experiments. Scale bar: 5 μm. See also Figures S4B and S4C. (C) 293T cells were co-transfected with plasmids encoding the NLRP3 inflammasome components NLRP3, ASC, NEK7, caspase-1, and pro-IL-1β, in addition to either C1 or a GFP control. Following nigericin stimulation, IL-1β concentrations in supernatants were quantified by ELISA. n = 3 biological replicates. Two-way ANOVA with Šídák’s multiple comparisons test. Data are represented as mean ± SD. (D) 293T cells were co-transfected as in (C) but with NLRP3 replaced by an empty vector. Cells were not stimulated with nigericin. IL-1β concentrations in supernatants were quantified by ELISA. n = 3 biological replicates. Paired t test. Data are represented as mean ± SD. (E) 293T cells were co-transfected as in (C) but with ASC replaced by an empty vector where indicated. Cells were not stimulated with nigericin. IL-1β concentrations in supernatants were quantified by ELISA. n = 3 biological replicates. Two-way ANOVA with Šídák’s multiple comparisons test. Data are represented as mean ± SD. (F) Vaccinia replication (inoculum MOI of 1) in LPS-primed murine macrophages was measured by TCID50. Data are represented as mean ± SD. (G) LPS-primed murine macrophages were infected with wild-type (VC2) or C1L-deficient vaccinia virus at an MOI of 10, and IL-1β was quantified by ELISA 4 h post-infection. n = 3 biological replicates. Paired t test. Data are represented as mean ± SD.

The unique composition of C1 raises questions about its function during infections. Previous studies demonstrated that myxoma M013 protein regulates host immunity by interfering with NF-κB-dependent signaling.^46^ We assessed whether C1 or its individual domains had similar functions to M013. In an assay measuring NF-κB signaling following tumor necrosis factor α (TNF-α) treatment, C1 protein did not phenocopy M013, suggesting that these proteins serve distinct functions (Figure 4A), a result consistent with our phylogenetic analyses suggesting that C1 protein is not an ortholog of M013. Curiously, the pyrin domain of C1 alone enhanced cellular responses to TNF-α, which potentially implicates the Bcl-2 domain in a role preventing aberrant activation of cellular inflammatory pathways by C1 during infection.

To further characterize C1 function, we considered whether it interacts with ASC, a critical pyrin-domain-containing protein, also known as pycard (pyrin- and CARD-domain-containing protein). ASC is an adapter protein that links innate immune sensors and caspases for multiple inflammasomes including AIM2 and NLRP3, two inflammasomes implicated in counteracting vaccinia virus infections.^15^ We found that ectopically expressed C1 protein associates with ASC by forming a concentrated shell peripheral to ASC specks (Figures 4B, S4B, and S4C). Surprisingly, the individual pyrin and Bcl-2 domains of C1 can separately associate with ASC (Figures S4B and S4C), suggesting multiple means of interfacing with ASC.

Because C1 appears to interact extensively with ASC, we tested whether C1 protein can modulate inflammasome signaling using a reconstituted NLRP3 inflammasome system.^35^ Unexpectedly, expression of C1 protein enhanced IL-1β release in the presence or the absence of the NLRP3 agonist nigericin, indicating that C1 protein directly promotes ASC-dependent caspase activation (Figure 4C). This increase in inflammasome activity was NLRP3 independent (Figure 4D) and relied upon ASC (Figure 4E). We found that both domains of C1 protein could promote ASC-dependent IL-1β processing (Figure 4E), consistent with our observations that individual domains of C1 protein can associate with ASC (Figures S4B and S4C).

The apparent promotion of inflammasome activation by C1 seems at odds with the diverse range of inflammasome inhibitors present in poxviruses, so we next asked whether C1 protein impacted vaccinia virus replication or inflammasome activity during infection. We generated C1-deficient vaccinia virus (Figures S4D and S4E) and found that it replicated at levels comparable to wild-type virus in BHK-21J cells (Figure S4F) and in inflammasome-sufficient macrophages (Figures 4F and S4G). However, we found decreased IL-1β secretion early during infections with C1-deficient vaccinia (Figure 4G), consistent with a pro-inflammatory role also observed from cell-based reporter assays. Together, these findings suggest that C1 protein enhances inflammasome signaling, adding a twist to poxvirus regulation of inflammasomes during infections as compared to a collection of inhibitory mechanisms.

## DISCUSSION

In the present study, we used *ab initio* structural modeling to search for cryptic homology between host and virus proteins. Our screen revealed two vaccinia virus proteins that modulate inflammasome activity, underscoring the importance of this immune pathway in viral infections. One, a homolog of gasdermins (A47), inhibits inflammasome function by interfering with inflammatory caspases. Another, a unique pyrin-Bcl-2 fusion protein (C1), is counterintuitively pro-inflammatory, driving ASC-dependent inflammasome activity.

Homology with gasdermin proteins guided our mechanistic studies of A47. A previous study^37^ established that A47 has no impact on viral replication in permissive cells such as BHK-21J cells, which is consistent with our findings using the same cell line (Figure S4E). The involvement of gasdermins in pyroptosis led us to explore the role of A47 in macrophages, where we found that A47 contributes to viral replication (Figure 2E) and suppression of cytokine release (Figure 2F). It is notable that the A47-dependent phenotypes are subtle, perhaps due to redundant mechanisms by which vaccinia virus inhibits inflammasome activity and pyroptosis.^23^ While combinatorial knockouts of A47 and other antipyroptotic proteins may be informative, we note the potential for homology screens to reveal overlapping functions and inform mechanistic study.

Our results support a model in which vaccinia A47 dampens inflammasome signaling and pyroptosis by interfering with inflammatory caspases. However, the presence of up to five viral gasdermin paralogs in some poxvirus genomes (Figures 2B, 2C, and S2E) suggests that they may have diverged to target inflammasome activity and pyroptosis through multiple mechanisms, perhaps with individual paralogs specializing in unique inhibitory roles. It is also possible that orthologs within the distinct clades of viral gasdermins adopted distinct functions as poxviruses adapted to infect different hosts. Future mechanistic studies will shed light on how different viral gasdermins work to benefit viral infection.

Several gene fusions in poxvirus genomes have been described, though in many cases, the contributions of individual domains to the function of hybrid proteins have not been explored.^16^ Our study identified a cryptic pyrin domain in the poxvirus Bcl-2 family protein C1 (Figure 3). To our knowledge, this is the only known example of a pyrin-Bcl-2 fusion protein, and the combination of these domains by poxviruses presents a unique opportunity to explore new facets of the biology of both domains. Curiously, the pyrin and Bcl-2 domains of C1 generally are redundant in function, with a notable difference being the ability of the pyrin domain to enhance NF-κB signaling following stimulation with TNF-α (Figure 4A). We suspect that this “masking” is not the only function unique to the individual domains of C1 protein and anticipate that future genetic and biochemical studies of this protein will expand our understanding of the complexities of inflammasome activity in poxvirus infection.

Unlike an inhibitor of inflammasome signaling such as A47, a pro-inflammatory protein is difficult to reconcile with a beneficial role for a virus. One possibility is that pro-inflammatory signals promoted by C1 protein allow vaccinia to recruit and infect cells such as macrophages, as infected macrophages may promote vaccinia dissemination.^52^ However, previous experimental infections of mice showed that macrophages are a critical component of the immune response to vaccinia infection.^53^ In contrast, it is possible that the promotion of inflammasome signaling by C1 is detrimental to poxviruses, though its retention in multiple poxvirus lineages (Figure 3E) is consistent with it conferring a selective advantage to poxviruses, at least in some virus-host combinations. Future *in vivo* pathogenesis studies of C1-deficient vaccinia would help better define the role of this protein, as well as of inflammation and macrophages, during vaccinia infection.

Identification of a novel domain organization in C1 protein raises the prospect that diverse gene fusion events are more common in viruses than currently appreciated. Freed from constraints in host genomes, individual domains and combinations thereof may diverge to acquire new functions, with viral genomes serving as a testbed of evolutionary innovation. Our study joins a growing body of work demonstrating the transformative power of structural modeling in identifying evolutionary connections^10,54,55^ and informing mechanistic study.^11^ The ability to detect distant homology is especially important for hybrid proteins such as C1, where global homology searches may be inconclusive (Figures 3A–3C). Tools such as Foldseek^56^ and the AlphaFold-based domain identification tool DPAM^57^ are beginning to address these limitations and will empower future studies of structural homology, with continuing computational advances unlocking new biology. Pipelines like ours have great potential to reveal cryptic captured genes in classes of viruses beyond poxviruses, such as the emerging class of giant viruses that can encode thousands of genes, deepening our understanding of how viruses manipulate their hosts during infection.

### Limitations of the study

To increase the specificity of our homology screens, we imposed rigid cutoffs for what we consider a “hit” indicative of shared structural features between virus and host proteins. While this was important for our selection of candidate proteins for mechanistic follow up, we may have excluded instances of homology that, while ambiguous given our methodology, could be of biological importance. While our data support a model in which A47 interferes with inflammatory caspases, leading to defects in downstream effects such as pyroptosis (Figure 2D) and IL-1β production (Figure 2E), it does not establish the precise mechanism by which it does so, and we therefore cannot conclude that A47 is a *bona fide* caspase inhibitor. Our work does not distinguish between a model in which A47 is a competitive inhibitor that occludes the GSDMD exosite of inflammatory caspases and one in which it promotes caspase degradation by serving as a degron. Additionally, while our study establishes both a functional interaction and co-localization of C1 and ASC using ectopic expression, it remains to be determined whether C1 associates with ASC during viral infection. Furthermore, while our experimental and imaging analysis suggest that C1 protein associates with ASC through multiple interaction interfaces, the nature of these interactions, such as which domains of ASC interface with which domains of C1, is currently unclear.

## STAR★METHODS

### RESOURCE AVAILABILITY

#### Lead contact

Further information and requests for resources and reagents should be directed to and will be fulfilled by the Lead Contact, Nels Elde (nelde@genetics.utah.edu).

#### Materials availability

All reagents generated in this study are available on request from the Lead Contact.

#### Data and code availability

- Original/source data for the current study are available from the Lead Contactupon request. Scripts generated and used in this study are included in Data S1.
- Atomic coordinates for eptesipox gasdermin have been deposited in the protein data bank (PDB) under accession 8GBE.
- Any additional information required to reanalyze the data reported in this work paper is available from the Lead Contact upon request.

### EXPERIMENTAL MODEL AND SUBJECT DETAILS

#### Cell culture

BHK-21J (male) (ATCC) and 293T (female) (a kind gift from Wes Sundquist, University of Utah) and HeLa (female) (a kind gift from Adam Geballe, Fred Hutchinson Cancer Research Center) cells were cultured in DMEM supplemented with 10% FBS. Transduced cells were maintained in the presence of 4μg/mL puromycin. Immortalized mouse bone marrow derived macrophages (sex unknown [not reported by source; data from this cell line therefore cannot be used to determine whether there may be sex-specific differences in phenotypes observed with recombinant vaccinia virus strains]) (a kind gift from Sunny Shin, University of Pennsylvania) were cultured in RPMI supplemented with 10% FBS. All cells were cultured at 37°C in 5% CO_2_.

#### Microbe strains

*S. cerevisiae* strain W303 (a kind gift of Tom Dever), was grown at 30°C in a rotary incubator. Strains were stored as glycerol stocks at −80°C. *E. coli* strain BL21(DE3) (Thermo Scientific) was grown at 37°C with agitation. Chemically-competent *E. coli* was prepared using the Mix & Go system (Zymo Research) and stored at −80°C.

#### Viruses

Vaccinia strain Copenhagen (VC2)^58^ was propagated in BHK-21J cells by passaging. Virus was harvested by freezing plates at −80°C, thawing, and sonicating the entire contents of the well in a Qsonica Q500 cup-horn sonicator (50% amplitude, 2s on/2s off, 60s processing time). Lysates were cleared by centrifugation at >14,000×g for 3–5 minutes. Virus was subsequently aliquot and stored at −80°C prior to determination of titer.

### METHOD DETAILS

#### Lentiviral pseudoparticle production and transductions

All lentiviral pseudoparticles were generated by co-transfecting sub-confluent 293T cells with expression plasmids pSCRPSY (a kind gift of Paul Bieniasz, The Rockefeller University), pSPAX2 (a gift from Didier Trono (Addgene plasmid # 12260)), and VSV-glycoprotein at a ratio of 25:5:1 using Lipofectamine 3000 (ThermoFisher). Two to six hours post-transfection, media was replaced with DMEM containing 3% FBS. Supernatants were collected at 48h, cleared by centrifugation, supplemented with 20mM HEPES, aliquotted, and stored at −80°C.

Cells were transduced by passive infection. Briefly, lentivirus was added to a minimum volume of transduction media (3% FBS, DMEM, 4μg/mL polybrene, 20mM HEPES) and added to cells. Cells were allowed to rest with pseudoparticle-containing media for 1–2 hours before addition of complete medium.

#### Viral infections

BHK or immortalized bone-marrow derived macrophages cells were seeded at 200,000 cells per well in a 12-well plate one day prior to infection. Virus was added in a minimal volume of media (RPMI or DMEM + 1%FBS, 400μl), after which 600μl of the appropriate complete (10% FBS) media was added per well. Virus was harvested by freezing plates at −80°C, thawing, and sonicating the entire contents of the well in a Qsonica Q500 cup-horn sonicator (50% amplitude, 2s on/2s off, 60s processing time). Lysates were cleared by centrifugation at >14,000×g for 3–5 minutes.

For viral stocks, virus was subsequently aliquot and stored at −80°C prior to determination of titer. Titers of experimental samples were immediately determined by TCID50.

#### TCID50 assays

Cells seeded at 20–30,000 cells per well on 96-well plates. Viral stocks were serially-diluted in DMEM supplemented with 1% FBS, and cells were infected with 50μl of inoculum for 2 hours, after which 100μl of DMEM supplemented with 10% FBS was added per well. Cells were scored for cytopathic effect (CPE) four to five days post-infection. For all experimental assays, samples were coded and randomized prior to TCID50 infections to ensure that the investigator was unbiased in assessing for CPE. TCID50 values were calculated by the method of Spearman and Kärber.

#### Recombinant vaccinia production

To generate recombinant vaccinia, BHK-21J cells were seeded on 6-well plates at 400,000. The next day, cells were infected at MOI of 2–5 in a 200μl of DMEM supplemented with 1% FBS. One hour post-infection, cells were transfected with 2μg of a homology donor plasmid containing a monomeric ultrastable GFP (muGFP)^59^ marker using Lipofectamine 3000 (3.5μl of lipofectamine per microgram of plasmid, 2μl of p3000 reagent per microgram). Media was changed four to six hours post-transfection. The next day, virus was harvested as indicated above.

Recombinant virus was subsequently purified by 2–3 rounds of limiting dilution assays on 96 well plates, followed by two rounds of plaque purification on 6w plates. Purity of recombinant virus was verified by PCR on viral stocks, as described below. At each stage, recombinant virus was identified by GFP expression.

ΔA47L virus was generated by fully deleting the A47L ORF, replacing it with muGFP. ΔC1L virus was generated by introducing an in-frame stop codon and partial deletion of the C1L ORF to avoid disrupting N1L, which partially overlaps with C1L.

#### Isolation of viral genomic DNA

For PCR: One volume of viral stock (typically 50ml) was mixed with one volume of digestion buffer (0.9% NP50, 0.9% Tween-20, 20mM Tris-HCl pH 8.3, 3mM MgCl_2_, 100mM KCl). The mixture was supplemented with 1/20 volume of Proteinase K at 20mg/mL, and the reaction was incubated at 40°C for 45 minutes. The completed reaction was transferred to a fresh tube and heat-killed by incubation at 95C for 10 minutes. This crude genomic DNA prep was used as input (1/10 reaction volume) for PCR analysis of viral DNA.

For nanopore sequencing: A sub-confluent 10cm dish of BHK-21J cells was infected at an MOI of 1 for 24h. Cells were subsequently scraped, pelleted, washed with PBS, and high molecular-weight DNA from approximately 2E6 cells was isolated using a Circulomics Nanobind CBB kit (cat# NB-900–001-01) per manufacturer protocol.

#### PCR genotyping of viral stocks

For all recombinant viruses, four PCRs were performed. Primers flanking the 5′ and 3′ regions of the insertion region were paired with primers specific to either the GFP insertion or the native sequence. Recombinant virus was only considered to be pure if both recombinant PCRs were positive and both native PCRs were negative. Both water and wild-type VC2 DNA was included as controls in all assays.

#### Nanopore sequencing of recombinant vaccinia virus

1.5μg of high molecular weight DNA was used as input for library prep using Oxford Nanopore’s V14 ligation sequencing kit (Oxford Nanopore, SQK-LSK114).

#### LPS electroporation assay

HeLa cells were plated one day prior to transfection at 250,000 cells per well on a 6w plate. The next day, cells were transfected with 2μg of the indicated constructs using Lipofectamine 3000 (2μl Lipofectamine 3000 per microgram of DNA and 2μl of p3000 reagent per microgram of DNA). Media was changed to fresh DMEM supplemented with 10% FBS six hours post-transfection. Two days after transfection, cells were electroporated with one microgram of LPS per million cells (or mock) using a Neon transfection system (1300V, 30ms pulse width, 1 pulse) (ThermoFisher). Cells were returned to pre-warmed DMEM supplemented with 10% FBS in 96-well plates for cell titer glo viability assays.

#### NF-κB reporter assay

One day prior to transfection, 293T cells were seeded at a density of 50,000 cells per well on opaque 96 well plates. Cells were transfected with 200ng of the indicated pEF construct, 50ng of pNFκBpro-FLuc (A kind gift of Neal Alto, UT Southwestern Medical Center), and 10ng pRL-TK (Promega) per well using Lipofectamine 3000 (0.7μl lipofectamine per microgram of plasmid DNA, 2μl of p3000 reagent per microgram of DNA). 24 hours post-transfection, media was changed to 75μl of DMEM supplemented with 10% FBS containing TNF-α (R&D Systems 210-TA) in either a dilution series or at approximately two times the EC50 within this assay, 1ng/mL, or a vehicle control. Six hours after treatment, plates were sealed and stored at −80°C until assay. Promoter activity (firefly luciferase) was determined by Dual-Glo luciferase assay (Promega) following manufacturer specifications, using Renilla signal as a transfection control. Luciferase readings were performed using either a BioTek Synergy HT or a BioTek H1 plate reader.

#### Reconstituted NLRP3 inflammasome assay

The murine NLRP3 inflammasome was reconstituted in 293T cells per an established protocol.^35^ Briefly, 200,000 293T cells were seeded in a 24 well plate 18–24 hours prior to transfection. Cells were co-transfected with pCMV-pro-IL1β-C-Flag (200ng), pcDNA3-N-Flag-NLRP3 (200ng), pcDNA3-N-Flag-ASC (20ng), pcDNA3-N-Flag-Caspase-1 (100ng), pcDNA3-N-HA-NEK7 (200ng), and the indicated pEF (300ng) construct. For some assays, an empty CMV plasmid was used in place of indicated inflammasome components.

24 hours post-transfection, media was changed to 250μl of DMEM + 10% FBS. For some assays, cells were stimulated with nigericin (12.5 or 25 μg per well) diluted in ETOH or a vehicle control one hour prior to harvest. Six hours post-media change, supernatant was collected, cleared by centrifugation, and stored at −80 prior to downstream assays.

For western blots of reconstituted cells, all cells, including those which were non-adherent at the time of harvest, were collected by centrifugation and resuspended in Laemmli buffer.

#### ELISA

For all ELISA assays, supernatants were clarified by centrifugation at >14,000×g for 3–5 minutes. Cleared supernatants were frozen at −80°C prior to assay. Supernatants were either assayed without dilution (most viral infections) or at an appropriate dilution in NS dilution buffer (Abcam).

Murine IL-1β was assayed using SimpleStep ELISA (Abcam ab197742). Briefly, samples and antibodies (detection and capture) were added to a pre-bound plate. Plates were incubated at room temperature for one hour with shaking, after which the plate was washed three times and TMB solution was added. Following 15–20 minutes of development, stop solution was added to all wells and OD_450_ absorbance was read on a BioTek HT plate reader. Concentrations of IL-1β were determined by comparison with a standard curve. All standards and experimental samples were assayed in technical duplicates.

#### Bacterial gasdermin toxicity assay

BL21(DE3) cells were transformed with pETDuet-1 plasmids containing the gasdermin D NTD and other orfs (gasdermin D CTD, GFP, or vaccinia gasdermin) under control of separate T7 promoters. Individual colonies were picked and grown to saturation overnight at 37°C. The next morning, overnight cultures were diluted to an OD_600_ of 0.1 in LB and were subsequently serially diluted in LB. 5μl drops of each dilution were spotted onto pre-warmed plates containing carbenicillin or carbenicillin supplemented with IPTG and X-GAL. Plates were incubated for approximately 20 hours at 37°C and imaged with a GelDoc (Bio-Rad).

#### Immunofluorescence

Cells were fixed with 4% PFA in PBS. Cells were washed with PBS, then permeabilized with 0.2% Triton-X 100. Cells were blocked with 5% goat serum in PBS for at least 30 minutes. Primary antibody was added in blocking solution and incubated for 1–2 hours. Cells were washed 3× with PBS, after which secondary antibody was added in 3% BSA and incubated for 30 minutes. Cells were washed 3× with PBS, and then mounted using Fluormount-G (Southern Biotech). Imaging was performed on a Zeiss LSM980. Images were processed in ImageJ. When made, linear adjustments were applied evenly across all samples within each experiment.

#### CellTiter-glo viability assays

For most viability assays, cells were seeded at 50,000 cells per well in 96 well plates. At the experimental endpoint, one volume of CellTiter-Glo (Promega) assay reagent was added to each well. Cells were incubated at room temperature for 10 minutes with shaking, after which luminescence was read on a BioTek Synergy HT plate reader.

#### Western blotting

Unless otherwise noted, cells were lysed directly in 1× SDS loading buffer (10% glycerol, 5% BME, 62.5mM TRIS-HCl pH 6.8, 2% SDS, and BPB), boiled, and sonicated (Qsonica Q500). Samples were run on “Any kD” acrylamide gels (Bio-Rad) and transferred to PVDF membranes using a Trans-Blot SD (Bio-Rad) or a Trans-Blot Turbo (Bio-Rad). Blots were blocked in 5% dry milk/TBS-T for 30 minutes to an hour at RT or overnight at 4°C. Primary antibodies were diluted in 5% dry milk/TBS-T and added for 1 to 2 hours at RT or overnight at 4°C. Blots were washed four times in TBS-T before addition of HRP-conjugated secondary antibody in 5% milk for thirty minutes. Blots were washed four times in TBS-T prior to detection with either ProSignal Dura (Prometheus) or Clarity ECL (Bio-Rad) substrate and exposure to radiography film or imaging on a C-Digit imager (LiCOR) or Azure Q500 (Azure).

#### Generation of yeast strains

A47L, the GSDMD CTD, and eGFP were cloned by PCR and restriction digest-based cloning into the stable expression vector pRS405. These cloned plasmids were then integrated into the *S. cerevisiae* W303 strain under -leucine selection. Expression of integrant proteins was confirmed by western blot. The GSDMD NTD was cloned into the pSB146 galactose inducible expression vector and transformed into all generated yeast-integrant strains under -uracil selection.

#### Yeast spot assays

Yeast strains were grown overnight in -Leu, -Ura glucose selective media at 30°C. The following day, yeast cultures were washed two times with water and diluted to an OD_600_ of 3.0. Each yeast strain was plated for spot assays by a five-fold dilution on both glucose and galactose (induction) plates. Spot assay plates were incubated at 30°C for one to three days prior to imaging.

#### Cytotoxicity assays

Cells were seeded and transfected as for CellTiter-Glo assays (see above). At the experimental endpoint, one volume of AAF-Glo assay reagent was added to cells and incubated for 10 minutes with shaking, after which an initial luminescence read was collected. One volume of digitonin-containing buffer was subsequently added to each well and cells were again incubated for 10 minutes with shaking to ensure complete lysis, after which a total luminescence read was collected. Percent cytotoxicity was calculated for each well.

#### Cloning

##### Transient overexpression clones

DNA encoding the A47L, C1L, M013L, and GSDMD sequences were synthesized (IDT, GeneWiz) and cloned into expression constructs by digest-based cloning. C1L truncations were cloned by PCR and restriction digest. Vaccinia A47L was cloned into the lentiviral expression plasmid pSCRPSY by LR recombinase reactions using gene-specific ATTB-containing primers. Vaccinia A47L and the GSDMD CTD were subcloned into the yeast expression plasmid pRS405 by digest from pCMV vectors. eGFP was cloned into pRS405 by PCR and digest-based cloning. The GSDMD NTD was cloned into the inducible yeast plasmid pSB146 by PCR and digest-based cloning. See annotated primer table (Table S3) for details.

##### Recombinant vaccinia donor plasmids

The A47L homology donor was synthesized and cloned into pUC19 (Genscript). The C1L homology donor was generated by PCR using VC2 (wild-type vaccinia virus strain Copenhagen) DNA as a template and sequential restriction digest-based cloning for the 3′ and 5′ homology arms. See annotated primer table (Table S3) for details.

#### Phylogenetic analyses

Multiple sequence alignments were generated using MUSCLE as implemented in MEGA 11.^60^ For gasdermin proteins (Figure 2C), avipox DNA polymerases (Figure S2H), and pyrin-domain containing proteins (Figure 3F), IQTREE^61^ was used to infer phylogeny.

#### Identification of viral homologs of vaccinia screen hits

NCBI BLAST^30^ and HMMER^31^ were used to identify homologs of vaccinia A47L and C1L. Alignments of all previously-identified A47 and C1 homologs were used for iterative HMMER searches until no new homologs were identified. TBLASTN, with known viral gasdermin protein sequences as queries, was used to search for pseudogenized viral gasdermins in poxvirus genomes.

#### Local poxvirus BLAST database searches

A local installation of BLAST+ (version 2.13.0)^62^ was used to generate a BLAST database comprised of representative poxvirus genomes (see Table S2). All identified poxvirus gasdermins were as search queries for BLASTN and discontinuous megablast searches to identify additional poxvirus proteins.

#### Homology search pipeline

We used existing tools to perform deep homology searches for the vaccinia virus proteome. First, AlphaFold2 (v2.1.2)^8^ was used as implemented at the University of Utah Center for High Performance Computing (CHPC) to generate models for all vaccinia virus proteins. Models with the overall greatest confidence (pLDDT) were used for homology searches.

Model organism (human, drosophila, mouse, and zebrafish) AlphaFold v2 proteomes were downloaded from the EBI AlphaFold Database to generate a target database for homology searches. TM-align searches were performed on a local Linux system (see Data S5), while FATCAT searches were performed on a high-performance cluster at the University of Utah CHPC.

FATCAT and TM-align results were processed and cleaned using BASH scripts (see Data S1). STRIDE^63^ was used to tabulate the number of secondary structures in protein models for filtering. Cleaned data were further processed and visualized using R^64^ (see Data S1). Additional R packages ggplot2,^65^ ggrepel,^66^ and ggnewscale^67^ were used to visualize data. Silhouettes of model metazoans used in Figure 1 were sourced from PhyloPic (phylopic.org) under a Creative Commons license.

#### Vaccinia virus genome assembly

Raw nanopore reads were mapped to a VC2 reference genome using minimap2.^68^ SAMtools^69^ was used to sort, index, and extract mapped reads. Flye^70^ was used to *de novo* assemble mapped reads for individual viruses for downstream processing. See Data S1 for additional details.

#### Comparison of sequenced genomes

NCBI BLAST (megablast) was used to align assembled vaccinia genomes. Resulting alignments and dot plot visualizations were used to assess recombinant viruses for off-target mutations.

#### Protein expression and purification

Poxvirus homologs of vaccinia *A47L* (VACV BAA01821.1, RCNV YP_009143477.1, EPTV YP_009408097.1, Yokapox A47L YP_004821504.1, Penguinpox YP_009046216.1, Flamingopox YP_009448152.1) were codon-optimized for *E. coli* expression, synthesized as gene fragments (IDT), and cloned into a custom pET vector encoding an N-terminal 63His-hSUMO2 solubility tag. Cloning was performed by Gibson assembly and N-terminal truncation variants were subcloned by PCR. All plasmids were verified by Sanger sequencing. Plasmids were transformed into BL21 CodonPlus(DE3)-RIL *E. coli*, grown as starter cultures in MDG media (0.5% glucose, 25mM Na_2_HPO_4_, 25mM KH_2_PO_4_, 50mM NH_4_Cl, 5mM Na_2_SO_4_, 2mM MgSO_4_, 0.25% aspartic acid, 100mg/mL ampicillin, 34mg/mL chloramphenicol, and trace metals), and expressed in M9ZB media (0.5% glycerol, 1% Cas-amino Acids, 47.8mM Na_2_HPO_4_, 22mM KH_2_PO_4_, 18.7mM NH_4_Cl, 85.6mM NaCl, 2mM MgSO_4_, 100mg/mL ampicillin, 34mg/mL chloramphenicol, and trace metals). M9ZB cultures were grown at 37°C with 230 RPM shaking to an OD_600_ of ~2.5. Protein expression was induced by cooling cultures on ice for 20 min and then supplementing cultures with addition of 0.5mM IPTG before incubation overnight at 16°C with shaking at 230 RPM. For each A47L variant, 2× 1L cultures were expressed, pelleted, flash frozen in liquid nitrogen, and stored at −80°C prior to purification.

All protein purification steps were performed at 4°C using buffers containing 20mM HEPES-KOH (pH 7.5). Expression cell pellets were thawed and lysed by sonication in buffer containing 400mM NaCl and 1mM DTT, clarified by centrifugation and glass wool, bound to NiNTA agarose beads (QIAGEN), washed with buffer containing 1M NaCl and 1mM DTT, and eluted with buffer containing 400mM NaCl, 300mM imidazole (pH 7.5), and 1mM DTT. The SUMO2 tag was cleaved by the addition of recombinant hSENP2 protease (D364–L589, M497A) to the NiNTA elution with overnight dialysis in buffer containing 125–250mM KCl and 1mM DTT. The resulting dialyzed protein was purified by size-exclusion chromatography using a 16/600 Superdex 75 column (Cytiva) pre-equilibrated with buffer containing 250mM KCl and 1mM TCEP. Sized proteins were concentrated to >50mg/mL using 10 kDa molecular weight cut-off concentrators (Millipore), flash frozen on liquid nitrogen, and stored at −80°C. Selenomethionine(SeMet)-substituted proteins were prepared in M9 media and purified in buffers containing 1 mM TCEP in place of DTT.

#### Protein crystallization and structure determination

Of the homologs and variants tested, Eptesipox virus (EPTV) gasdermin (GenBank accession ASK51347) was found to have optimal yield and solubility. Eptesipox gasdermin crystals were grown by the hanging-drop vapor diffusion method at 18°C using NeXtal crystallization screens and optimized with EasyXtal 15-well trays (NeXtal) and the Slice pH screen (Hampton Research). Proteins for crystallization were thawed from −80°C stocks on ice and diluted to concentrations of 20mg/mL protein and 20mM HEPES-KOH (pH 7.5), 60mM KCl, and 1mM TCEP. 15-well crystal trays were set with 2mL drops containing diluted protein and reservoir at a 1:1 ratio over wells with 350μL reservoir. Crystals were grown for at least 3 days, incubated in reservoir solution without additional cryoprotectant, and harvested by flash freezing in liquid nitrogen. Native EPTV gasdermin (amino acids 12–217) crystals grew in 100mM ADA (pH 6.4) and 40% PEG-200. SeMet-substituted EPTV gasdermin (amino acids 18–217) crystals grew in 100mM DL-malic acid (pH 5.3) and 40% PEG-200. All crystals were cryoprotected in reservoir solution. X-ray diffraction data were acquired using Northeastern Collaborative Access Team beamlines 24-ID-C and 24-ID-E (P30 GM124165), and used a Pilatus detector (S10RR029205), an Eiger detector (S10OD021527) and the Argonne National Laboratory Advanced Photon Source (DE-AC02–06CH11357).

Data were processed with XDS and AIMLESS using the SSRL autoxds script (A. Gonzalez).^71^ All structures were phased with anomalous data from SeMet-substituted crystals using Phenix Autosol version 1.19.^72,73^ Atomic models were built in Coot^74^ and refined in Phenix using native diffraction data. Statistics were analyzed as described in Table S4.^75–77^ Structure data were deposited in the Protein DataBank (PDB ID 8GBE). Structure figures were generated using PyMOL version 2.4.0 (Schrö dinger, LLC).

### QUANTIFICATION AND STATISTICAL ANALYSIS

Statistical analyses were performed using GraphPad Prism unless otherwise noted. Unless otherwise indicated, all comparisons are relative to control (Ctrl, GFP, or VC2), as labeled. For data with two groups, two-tailed t tests were used. For data with more than two groups, ANOVA tests were used and appropriate adjustments were made for multiple hypothesis testing. Unless otherwise specified, P values are denoted as follows: n.s. not significant, *P<0.05, **P<0.01, ***P<0.001, ****P<0.0001. Additional details (n, definition of center, and dispersion and precision measures, and exact statistical tests used for each comparison) can be found in the corresponding Figure Legends for panels containing statistical comparisons.

## Supplementary Material

1

2

3

4

5

## Figures and Tables

**Table:** KEY RESOURCES TABLE

REAGENT or RESOURCE	SOURCE	IDENTIFIER

Antibodies		

Mouse-anti-beta-actin	BD Biosciences	612657
Rabbit-anti-FLAG (D6W5B)	CellSignaling Technology	14793
Rabbit-anti-MYC (71D10)	CellSignaling Technology	2278
Goat anti-Rabbit IgG (H+L) Cross-Adsorbed Secondary Antibody, Alexa Fluor^™^ 647	Invitrogen	A-21244
Goat-anti-Rabbit IgG HRP conjugate	MilliporeSigma	AP132
Goat-anti-Mouse IgG/IgM HRP conjugate	MilliporeSigma	AP130P
Goat-anti-mouse 700	Azure Biosystems	AC2129
Goat-anti-rabbit 800	Azure Biosystems	AC2134

Bacterial and virus strains		

Vaccinia virus strain Copenhagen (VC2)	Adam Geballe	N/A
VC2ΔA47L	This study	N/A
VC2ΔC1L	This study	N/A

Chemicals, peptides, and recombinant proteins		

Lipofectamine 3000	ThermoFisher	L3000015
Recombinant Human TNF-alpha Protein	R&D Systems	210-TA
Nigericin sodium salt	ThermoFisher	J61349.MA
Fluormount G	SouthernBiotech	0100–01
Hoechst 33342	Invitrogen	H3570

Critical commercial assays		

Mouse IL-1 beta ELISA Kit	Abcam	ab197742
Circulomics Nanobind CBB	Circulomics, PacBio	NB-900–001-01
V14 ligation sequencing kit	Oxford Nanopore	SQK-LSK114
DualGlo	Promega	E2920
CellTiter-Glo	Promega	G7571
CyTox-Glo	Promega	G9290

Deposited data		

Coordinates of eptesipox gasdermin	This paper	8GBE

Experimental models: Cell lines		

BHK-21J	ATCC	CCL-10
HEK293T	Wes Sundquist	N/A
HeLa	Adam Geballe	N/A
Bone marrow macrophages (immortalized)	Sunny Shin	N/A

Experimental models: Organisms/strains		

*S. cerevisiae*: Strain background: W303	Laboratory of Tom Dever	N/A
*E. coli*	Thermo Scientific	BL21(DE3), EC0114
*E. coli*	Agilent	BL21-CodonPlus (DE3) RIL, 230245

Oligonucleotides		

See Table S3 for oligonucleotide sequences		N/A

Recombinant DNA		

pCMV-Empty (pCDNA3.1 with alternate MCS)	Wes Sundquist	N/A
pCMV-vvA47L	This study	N/A
pCMV-GSDMD-CTD	This study	N/A
pCMV-GSDMD-NTD	This study	N/A
pEF1α-GFP (pCAG GFP with alternate promoter)	Wes Sundquist	N/A
pEF1α-M013L	This study	N/A
pEF1α-vvC1	This study	N/A
pEF1α-vvC1-PYD	This study	N/A
pEF1α-vvC1-BCL2	This study	N/A
pSCRPSY-Empty	Paul Bieniasz	N/A
pSCRPSY-vvA47L	This study	N/A
pVSV-Glycoprotein	Cell BioLabs	RV-110
pSPAX2	Didier Trono, Addgene	12260
pUC19-muGFP_donor_A47	This study	N/A
pUC19-muGFP_donor_C1	This study	N/A
pET-SUMO2-EPTVgsdm	This study	N/A
pRL-TK	Promega	E2231
pRS405	Laboratory of David Stillman	N/A
pRS405-vvA47L	This study	N/A
pRS405-GSDMD-CTD	This study	N/A
pRS405-GFP	This study	N/A
pSB146-GSDMD-NTD	This study	N/A
pET-Duet-1	MilliporeSigma	71146
pET-Duet-1-GSDMD-NTD-eGFP	This study	N/A
pET-Duet-1-GSDMD-NTD-GSDMD-CTD	This study	N/A
pET-Duet-1-GSDMD-NTD-vvA47L	This study	N/A
pNFkBpro-FLuc	Neal Alto	
pCMV-pro-IL1b-C-FLAG	Bruce Beutler, Addgene	75131
pcDNA3-N-FLAG-NLRP3	Bruce Beutler, Addgene	75127
pcDNA-N-FLAG-ASC1	Bruce Beutler, Addgene	75134
pcDNA3-N-FLAG-Caspase-1	Bruce Beutler, Addgene	75128
pcDNA-N-HA-NEK7	Bruce Beutler, Addgene	75142
pLEX-MCS-ASC-GFP	Christian Stehlik, Addgene	73957

Software and algorithms		

AlphaFold2 v 2.1.2	DeepMind	https://github.com/deepmind/alphafold
TM-Align	Zhang Lab	https://zhanggroup.org/TM-align/
FATCAT	Godzik Lab	https://github.com/GodzikLab/FATCAT-dist
STRIDE	Frishman and Argos	https://github.com/MDAnalysis/stride
Flye	Mikhail Kolmogorov	https://github.com/fenderglass/Flye
SAMtools	Genome Research Limited	https://github.com/samtools/samtools
minimap2	Heng Li	https://github.com/lh3/minimap2
BLAST+ v 2.13.0	NCBI	https://ftp.ncbi.nlm.nih.gov/blast/executables/blast+/LATEST/
SSRL autoxds	A. Gonzalez	https://smb.slac.stanford.edu/facilities/software/xds/
Phenix Autosol v 1.19	Phenix	https://phenix-online.org/documentation/reference/autosol.html
Coot	Paul Emsley	https://github.com/pemsley/coot
R v 4.2.2	The R Foundation	r-project.org
R Studio v 2022.12.0+353	RStudio, Inc	Rstudio.com
ggplot2 R package	H Wickham	https://cran.r-project.org/web/packages/ggplot2/index.html
ggrepel R package	Kamil Slowikowski	https://cran.r-project.org/web/packages/ggrepel/
ggnewscale R package	Elio Campitelli	https://cran.r-project.org/web/packages/ggnewscale/index.html
Fiji	ImageJ	https://imagej.net/software/fiji/
Mega 11	Tamura, Stecher, and Kumar	Megasoftware.net
IQTREE	http://www.iqtree.org/	http://www.iqtree.org/
Figtree V1.4.4	Andrew Rambaut	http://tree.bio.ed.ac.uk/software/figtree/
PyMOLv 2.4.0	Schrödinger, LLC	N/A
GraphPad Prism 9	GraphPad Software	https://www.graphpad.com/scientific-software/prism/

Other		

Zeiss LSM980	Zeiss	N/A
BioTek Synergy HT	Agilent	N/A
BioTek H1	Agilent	N/A
Qsonica Q500	Qsonica	N/A
Azure 500Q	Azure Biosystems	N/A

## Highlights

- AlphaFold-based homology searches reveal vaccinia homologs of host proteins
- X-ray crystallography confirms that vaccinia A47 is a gasdermin homolog
- A47 interferes with inflammasome activity
- C1 is a unique, pro-inflammatory pyrin-Bcl-2 fusion protein
