# Supplementary material for: Structural homology screens reveal host-derived poxvirus protein families impacting inflammasome activity
Source: Cell Rep. Author manuscript; Available in PMC 2023 Dec 12. (PMC10715236; doi:10.1016/j.celrep.2023.112878)
Supplement: 1 [file NIHMS1928244-supplement-1.pdf]

Cell Reports, Volume *42*

Supplemental information

Structural homology screens reveal host-derived poxvirus protein families impacting inflammasome activity

Ian N. Boys, Alex G. Johnson, Meghan R. Quinlan, Philip J. Kranzusch, and Nels C. Elde

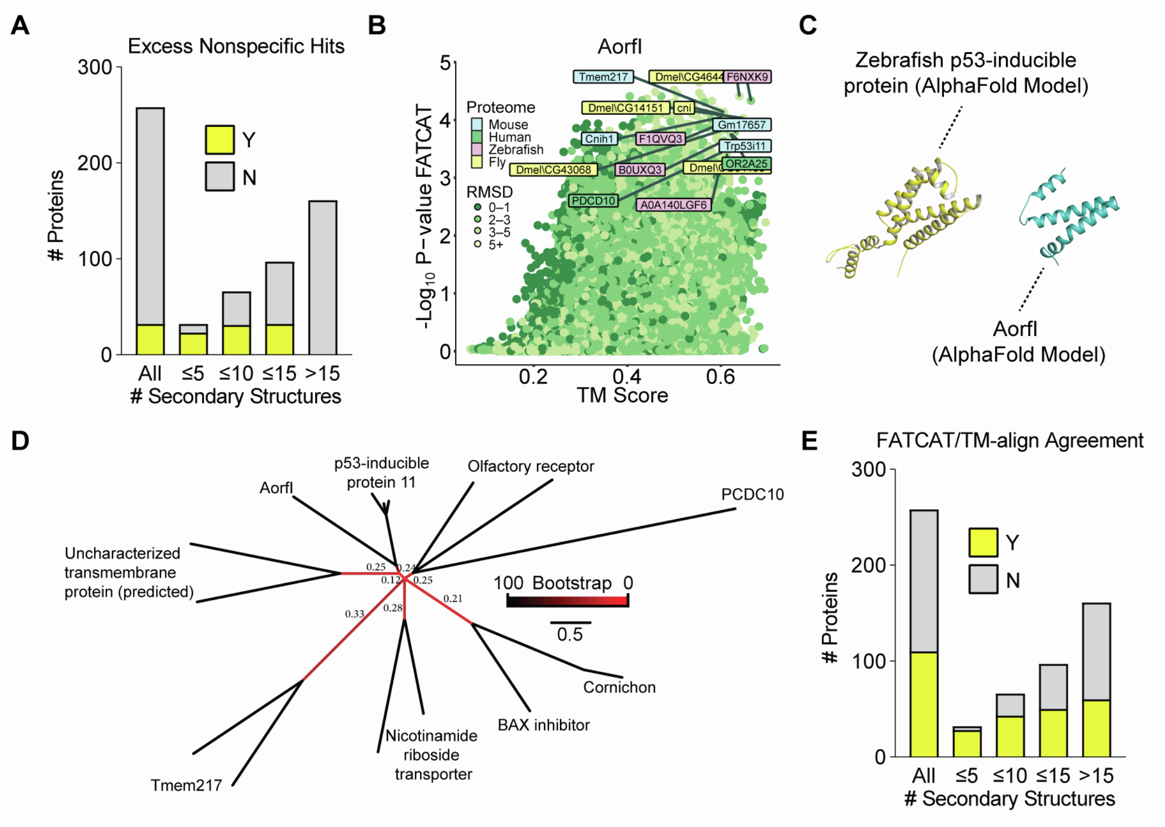

Figure S1. Structure-based homology screen optimization, Related to Figure 1

a. Tabulation of nonspecific hits by number of protein secondary structures as determined by STRIDE.
b. FATCAT and TM-align results for A orf I. Only a subset of the 100+ "hits" are indicated.
c. Comparison of AlphaFold models of A orf I and danio p53-inducible protein 11.
d. Maximum likelihood tree of the hits indicated in Figure S1A. Select bootstrap values from 100 replicates are indicated.
e. Tabulation of proteins for which TM-align and FATCAT converged by number of protein secondary structures determined by STRIDE.

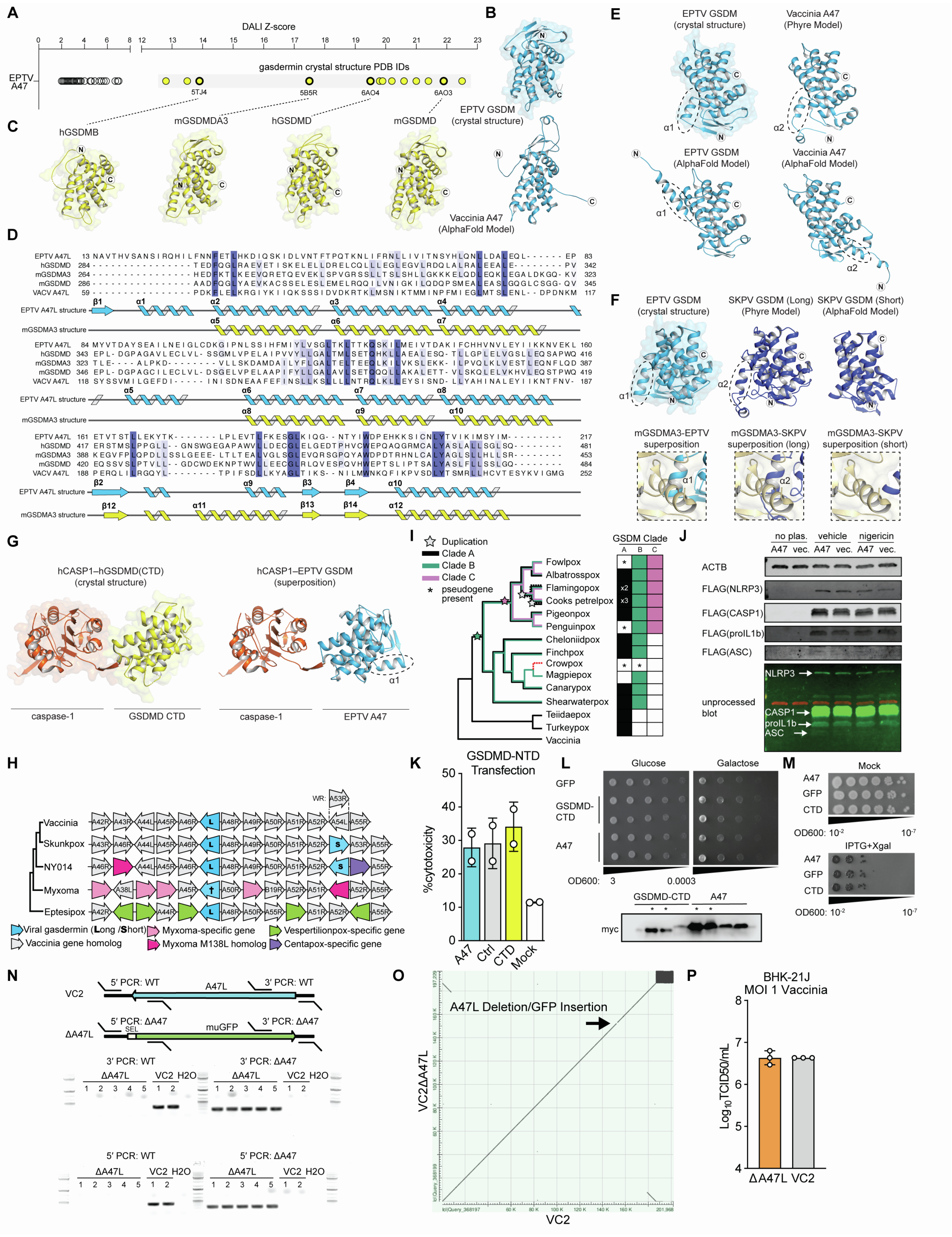
A
DALI Z-score
EPTV A47
gasdermin crystal structure PDB IDs
5TJ4
5B5R
6AO4
6AO3
B
C
hGSDMB
mGSDMDA3
hGSDMD
mGSDMD
EPTV GSDM (crystal structure)
Vaccinia A47 (AlphaFold Model)
D
EPTV A47L
hGSDMD
mGSDMA3
mGSDMD
VACV A47L
EPTV A47L structure
mGSDMA3 structure
E
EPTV GSDM (crystal structure)
Vaccinia A47 (Phyre Model)
EPTV GSDM (AlphaFold Model)
Vaccinia A47 (AlphaFold Model)
F
EPTV GSDM (crystal structure)
SKPV GSDM (Long) (Phyre Model)
SKPV GSDM (Short) (AlphaFold Model)
mGSDMA3-EPTV superposition
mGSDMA3-SKPV superposition (long)
mGSDMA3-SKPV superposition (short)
G
hCASP1–hGSDMD(CTD) (crystal structure)
hCASP1–EPTV GSDM (superposition)
caspase-1
GSDMD CTD
caspase-1
EPTV A47
H
WR: A53R
Vaccinia
Skunkpox
NY014
Myxoma
Eptesipox
Viral gasdermin (Long /Short)
Vaccinia gene homolog
Myxoma-specific gene
Myxoma M138L homolog
Vespertilionpox-specific gene
Centapox-specific gene
I
Duplication
Clade A
Clade B
Clade C
* pseudogene present
GSDM Clade
A B C
Fowlpox
Albatrosspox
Flamingopox
Cooks petrelpox
Pigeonpox
Penguinpox
Cheloniidpox
Finchpox
Crowpox
Magpiepox
Canarypox
Shearwaterpox
Teiidaepox
Turkeypox
Vaccinia
J
no plas.
vehicle
nigericin
A47 vec. A47 vec. A47 vec.
ACTB
FLAG(NLRP3)
FLAG(CASP1)
FLAG(proIL1b)
FLAG(ASC)
unprocessed blot
NLRP3
CASP1
proIL1b
ASC
K
GSDMD-NTD Transfection
%cytotoxicity
A47
Ctrl
CTD
Mock
L
Glucose
Galactose
GFP
GSDMD-CTD
A47
OD600: 3
0.0003
GSDMD-CTD
A47
myc
M
Mock
A47
GFP
CTD
IPTG+Xgal
N
5′ PCR: WT
3′ PCR: WT
A47L
VC2
5′ PCR: ΔA47
3′ PCR: ΔA47
SEL
muGFP
ΔA47L
3′ PCR: WT
3′ PCR: ΔA47
5′ PCR: WT
5′ PCR: ΔA47
ΔA47L
VC2
H2O
O
A47L Deletion/GFP Insertion
VC2ΔA47L
VC2
P
BHK-21J
MOI 1 Vaccinia
Log10TCID50/mL
ΔA47L
VC2

Figure S2. Properties of a poxvirus-encoded gasdermin homolog, Related to Figure 2

a. DALI Z-scores from searching EPTV gasdermin structure against the full Protein Data Bank (PDB). Results for multiple chains were manually removed. PDB entries that represent gasdermin crystal structures in the PDB were highlighted yellow, and select PDB entries and the structures of four select structures are show in panel c.
b. Experimental and predicted structures of poxvirus gasdermin proteins reveal gasdermin homology. Top, crystal structure of the Eptesipox virus (EPTV) gasdermin. Bottom, AlphaFold predicted structure of vaccinia virus (VACV) gasdermin. The N- and C-termini are indicated with the circled letters N and C.
c. Representative published crystal structures of human (h) or mouse (m) gasdermins from the top EPTV A47L DALI hits shown in panel a. Shown structures are of the hGSDMB CTD (PDB ID 5TJ4), mGSDMA3 (PDB ID 5B5R, residues 264–453), hGSDMD CTD (PDB ID 6AO4), and mGSDMD (PDB ID 6AO3). The N- and C-termini are indicated with the circled letters N and C.
d. Structure-based sequence alignment of the EPTV gasdermin crystal structure and structures from panels b and c. The hGSDMB structure was excluded due to large gaps in alignment, as were the first 58 residues of the VACV gasdermin AlphaFold predicted structure. The secondary structure of mGSDMA3 is based on the numbering scheme used previously [1].
e. Comparison of the EPTV and vaccinia AlphaFold models with the EPTV crystal structure and a homology-based Phyre2 [2] structural prediction of vaccinia A47, using EPTV structural data as a reference.
f. Comparison of EPTV gasdermin with AlphaFold and Phyre models of viral gasdermins from a poxvirus containing both “long” and “short” gasdermins, skunkpox virus.
g. Comparison of a crystal structure of hCASP1-hGSDMD(CTD) (PDB ID 6KN0, left) with a superposition of EPTV gasdermin on the hGSDMD(CTD) of the co-crystal structure (right). The hGSDMD(CTD) model is omitted from the superposition.
h. Synteny analysis of viral gasdermins. When applicable, genes are labeled based on their vaccinia homologs. †: The evolutionary history of leporipox gasdermin is ambiguous (see main text).
i. Reconstructed evolutionary history of viral gasdermins in avipoxviruses. Tree represented a cladogram derived from a maximum-likelihood tree constrtucted from an alignment of DNA polymerase from the indicated poxviruses. All nodes are supported by over 50% of 1000 bootstrap replicates. Gain/loss events were inferred based on this phylogeny.
j. Western blot of transfected NLRP3 inflammasome components in 293T cells expressing either A47 or a vector control. Representative western blot of n=3 biological replicates. Related to Figure 2E.
k. 293T cells were co-transfected with a 1:2 ratio of a plasmid expressing GSDMD-NTD and plasmids expressing either vaccinia A47, the GSDMD-CTD, or a vector control. Cytotoxicity was determined by CyTox-Glo (Promega) 24 hours post-transfection. n=2 biological replicates
l. Top: Yeast integrants expressing vaccinia A47, GSDMD-CTD, or eGFP as a control were transformed with a galactose-inducible plasmid encoding the GSDMD NTD. Cultures were spotted on plates containing glucose or galactose to assess NTD-mediated toxicity. Representative image of n=5 biological replicates. Bottom: western blot of GSDM expression in integrant strains. Integrants denoted with an (*) were selected for spot assays.
m. *E. coli* BL21-DE3 cells were transformed with bicistronic plasmids encoding the indicated proteins and the GSDMD-NTD under control of a T7 promoter. Colonies were grown overnight, diluted to the indicated ODs, and spotted in the presence or absence of galactose to induce protein expression. Representative image of n=5 biological replicates.
n. Top: schematic of A47 locus for VC2 and ΔA47L recombinant virus with PCR genotyping primers indicated. Bottom: PCR genotyping of recombinant ΔA47L virus. VC2 (wild-type) is included as a control. ΔA47L clonal stock 1 was used for experiments.
o. Megablast-based dot plot alignment of wild-type VC2 and VC2-ΔA47L vaccinia virus.
p. BHK-21J cells were infected with wild-type (VC2) or A47L-deficient vaccinia virus at an MOI of 1 and viral titer was quantified by TCID50 24 hours post-infection. n = 3 biological replicates.

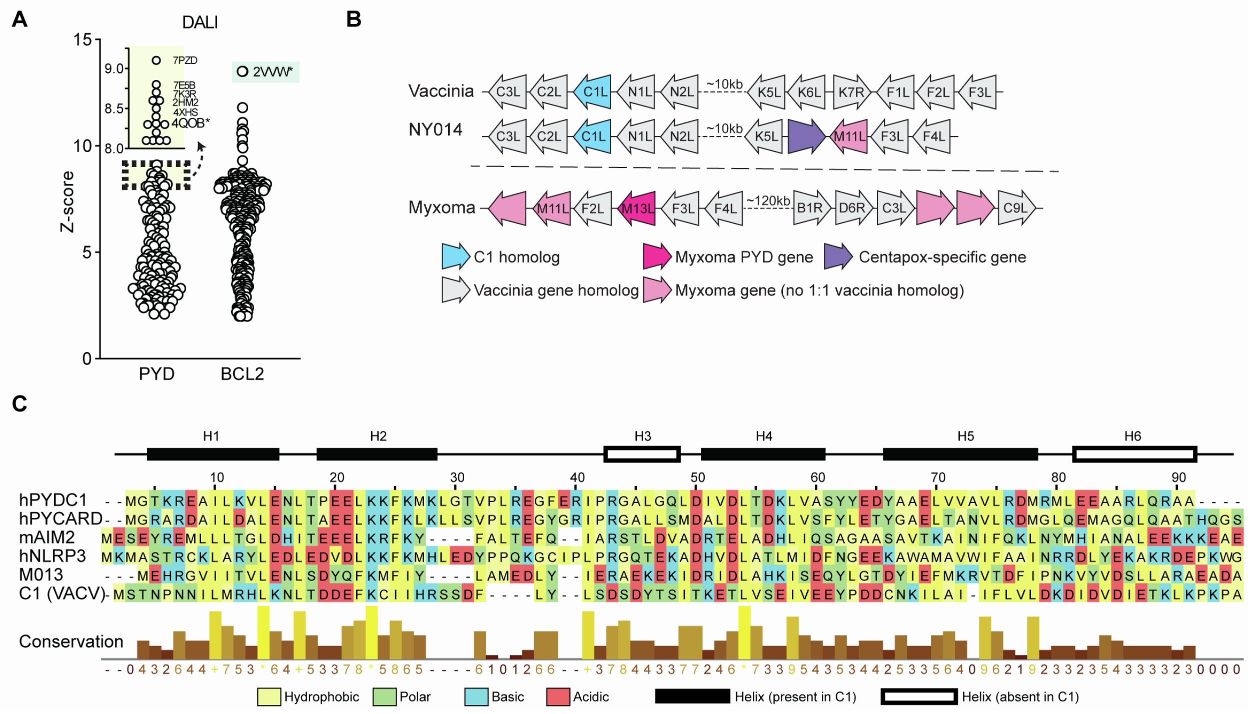

Figure S3. Properties of C1 protein, a poxvirus pyrin-Bcl2 fusion protein, Related to Figure 3

a. DALI Z-scores from searching C1 subdomain AlphaFold models against the full Protein Data Bank (PDB).
b. Synteny analysis of C1 and M013 families. When applicable, genes are labeled based on their vaccinia homologs.
c. Amino acid alignment of select pyrin domains. Helices denoted are those present in ASC [3].

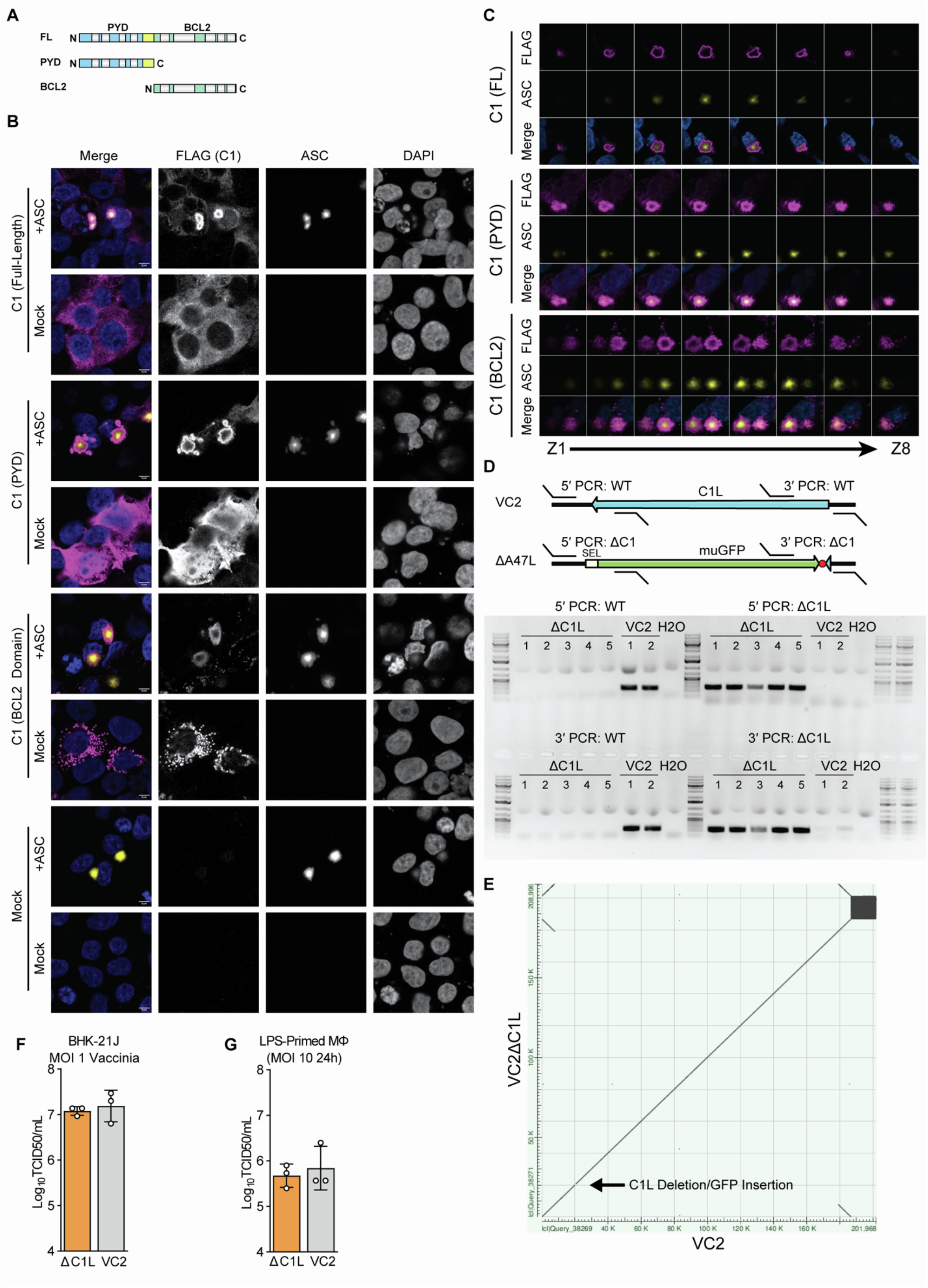

Figure S4. Characterization of C1 protein, Related to Figure 4

a. C1 truncations used in this study. Helices (grey boxes) and the PYD-Bcl-2 linker (yellow) are indicated.
b. 293T cells were co-transfected with the indicated plasmids and either ASC-GFP [4] or an empty vector. 24 hours post-transfection, cells were fixed and stained for FLAG (C1) and subsequently imaged. Representative image

from n=3 independent experiments. Images for full-length C1 protein are the same as in Figure 4B. Scalebar: 5µm.

c. Z stacks of ASC specks in 293T cells co-transfected with ASC-GFP and the indicated FLAG-tagged C1-expressing constructs. Z stacks are from the same experiment shown in Figure 4B.
d. Top: schematic of C1L locus for VC2 and ΔC1L recombinant virus with PCR genotyping primers indicated. Red octagon represents the stop codon introduced in-frame to preserve the coding sequence of N1L (see methods). Bottom: PCR genotyping of recombinant ΔC1L virus. VC2 (wild-type) is included as a control. ΔC1L clonal stock 2 was used for experiments.
e. Megablast-based dot plot alignment of wild-type VC2 and VC2-ΔC1L vaccinia virus.
f. BHK-21J cells were infected with wild-type (VC2) or C1L-deficient vaccinia virus at an MOI of 1 and viral titer was quantified by TCID50 24 hours post-infection. n = 3 biological replicates.
g. LPS-primed murine macrophages were infected with wild-type (VC2) or C1L-deficient vaccinia virus at an MOI of 10 and viral titer was quantified by TCID50 24 hours post-infection. n = 3 biological replicates. Samples are from the same experiment as Figure 6H.

Table S4. Crystallographic statistics, Related to Figure 2A and Figure S2A–D

	**EPTV A47L**	**EPTV A47L** (SeMet)
**Data Collection**		
Resolution (Å)[a]	48.65–1.46 (1.48–1.46)	48.39–1.54 (1.57–1.54)
Wavelength (Å)	0.9792	0.9792
Space group	C 2 2 2_1	C 2 2 2_1
Unit cell: a, b, c (Å)	64.46 74.14 95.60	63.51, 74.71, 95.29
Unit cell: α, β, γ (°)	90.00 90.00 90.00	90.00, 90.00, 90.00
Molecules per ASU	1	1
Total reflections	543256	920245
Unique reflections	40114	33934
Completeness (%)[a]	100.0 (99.6)	100.0 (99.9)
Multiplicity[a]	13.5 (13.2)	27.1 (23.4)
I/σI[a]	18.3 (1.6)	15.7 (2.1)
CC(1/2)[5] (%)[a]	99.9 (50.5)	99.9 (88.8)
Rpim[6] (%)[a]	1.9 (71.8)	3.0 (68.9)
Sites		5
**Refinement**		
Resolution (Å)	48.65–1.46	
Free reflections	40033	
R-factor / R-free	19.0 / 21.2	
Bond distance (RMS Å)	0.01	
Bond angles (RMS °)	1.10	
**Structure/Stereochemistry**		
No. atoms: protein	1658	
No. atoms: water	85	
Average B-factor: protein	32.8	
Average B-factor: water	43.1	
Ramachandran plot: favored	99.01%	
Ramachandran plot: allowed	0.99%	
Ramachandran plot: outliers	0.00%	
Rotamer outliers	0.52%	
MolProbity[7] score	1.05	
Protein Data Bank ID	8GBE	

[a] Highest resolution shell values in parentheses

Supplemental References

1. Ding, J., Wang, K., Liu, W., She, Y., Sun, Q., Shi, J., Sun, H., Wang, D.C., and Shao, F. (2016). Pore-forming activity and structural autoinhibition of the gasdermin family. Nature *535*, 111-116. 10.1038/nature18590.
2. Kelley, L.A., Mezulis, S., Yates, C.M., Wass, M.N., and Sternberg, M.J. (2015). The Phyre2 web portal for protein modeling, prediction and analysis. Nat Protoc *10*, 845-858. 10.1038/nprot.2015.053.
3. Liepinsh, E., Barbals, R., Dahl, E., Sharipo, A., Staub, E., and Otting, G. (2003). The death-domain fold of the ASC PYRIN domain, presenting a basis for PYRIN/PYRIN recognition. J Mol Biol *332*, 1155-1163. 10.1016/j.jmb.2003.07.007.
4. de Almeida, L., Khare, S., Misharin, A.V., Patel, R., Ratsimandresy, R.A., Wallin, M.C., Perlman, H., Greaves, D.R., Hoffman, H.M., Dorfleutner, A., and Stehlik, C. (2015). The PYRIN Domain-only Protein POP1 Inhibits Inflammasome Assembly and Ameliorates Inflammatory Disease. Immunity *43*, 264-276. 10.1016/j.immuni.2015.07.018.
5. Karplus, P.A., and Diederichs, K. (2012). Linking crystallographic model and data quality. Science 336, 1030-1033. 10.1126/science.1218231.
6. Weiss, M. (2001). Global indicators of X-ray data quality. Journal of Applied Crystallography 34, 130-135. doi:10.1107/S0021889800018227.
7. Chen, V.B., Arendall, W.B., 3rd, Headd, J.J., Keedy, D.A., Immormino, R.M., Kapral, G.J., Murray, L.W., Richardson, J.S., and Richardson, D.C. (2010). MolProbity: all-atom structure validation for macromolecular crystallography. Acta Crystallogr D Biol Crystallogr 66, 12-21. 10.1107/S0907444909042073.77.
