# Supplementary material for: Structural homology screens reveal host-derived poxvirus protein families impacting inflammasome activity
Source: Cell Rep. Author manuscript; Available in PMC 2023 Dec 12. (PMC10715236; doi:10.1016/j.celrep.2023.112878)
Supplement: 5 [file NIHMS1928244-supplement-5.zip › Data S1/S3File_Alignments_and_Trees.docx]

GSDM alignment

>MF001304.1:c165763-165224_Murmansk_poxvirus_strain_LEIV-11411_complete_genome

-------------------------------------------------------------------------------------------------------------MLSVPSQYKDLLQEYYDIITSVP-DEYRDIFVEYLIYLI-----------------------------EYDGRISNK-------------------RTRDIKKELINIV--GKVSDKTRLMNFISFITESICRLTRF-QSF-LL--ELASYHNVLDEVQEI---MGDIIEN----------------HCRME-IIGFTTYDI----------------------------------------------------------------------------------------------------NSIVLSIIYNMLKTSGMRVD-----------YDTSKAWWNPEYRDMFGS--LYMIVTT--LTILKNIX--------------------

>MF001305.1:c164808-164269_NY_014_poxvirus_strain_2013_complete_genome

-------------------------------------------------------------------------------------------------------------MLSVPSKYKDLLQEYYDIITSVP-DEYRDIFVQYLIYLI-----------------------------EYDGRISNK---------------------RTRDIKKELINIVGKVSDKTRLMNFISFITESICRLTRF-QSF-LL--ELASYHDVLDEVQEI---MGDIIKN----------------HCRMEIIGFTAYDIN-----------------------------------------------------------------------------------------------------SIVLSIIYNMLKTSGMRVD-----------YDTSKVWWNPEYSDMFGS--LYMIVTT--LTILKNIX--------------------

>KU749311.1:173861-174445_Volepox_virus_strain_CA_complete_genome

----------------------------------------------------------------------------------------------------------------MLNDYIGQIYIRCRAILSIP-DDYKHKFIKSLTHLI-------------------------RYNGRTYNRCTRD----------------------AITELSDIAN----NIGNNDVKIITTFIISSIYKITRF-QSF-LV--ELAMYHKVLDKIQHI---MEYIIEN-----HSHND------TNGFT-PYSTDDTNF----------------------------------------------------------------------------------------------------NQVIACIIYNLLKSSGMEIN-----------YSECKAWWNPRHDYVDTFGLLYIIVTI--LLIIDGDQYNFYNKLEKNKX--------

>KU749309.1:150174-150722_Raccoonpox_virus_strain_85A_complete_genome

--------MRDSYICQ--------------------------------------------------------------------------------------------------------IYTRCHSILSMP-DDYKYKFIKSLNHLI-------------------------RYNGRTYNRCTRD----------------------AITELSDIAN----SIDDTDIKIITTFIISSIYKITRF-QSF-LV--ELAMHHKVLDKVQHI---MEYIIDN-----HSHSN------TNGFT-PYSIDDPDF----------------------------------------------------------------------------------------------------NQVVACIIYNLLRSSGMDIN-----------YSKCKAWWNPRHDYVDTFGLLYVIFTI--LSIIGDKX--------------------

>KU749310.1:172437-172985_Skunkpox_virus_strain_WA_complete_genome

--------MQNSYIGQ--------------------------------------------------------------------------------------------------------IYTRCHAILLIP-DEYKHKFIKSLTHLI-------------------------GYNGRTYNRCTRD----------------------AITELTDIAN----EIDNTDVKLITTFIISSIYKITRF-QSF-LV--ELAMYHKVLDKIQHI---MEYIIDN-----HSRID------NNGFT-PYSDNDPDF----------------------------------------------------------------------------------------------------NQVIACIIYNLLRSSGMEIN-----------YSKYKAWWNPRHDYVDTFGLLYVIFTI--LSIIDDNX--------------------

>NP_051846.1_m132L_Myxoma_virus

--------MGNLLYCC-------------------------------------------------------------------------------------------FTVEKTVPSIQRYVNGQVNLDNFLA-RHRKRSILLYLLKVI--RDDKLLNE---------------VCRVKKKYSVYLY-------------------------GCKNPNA---------EVVNFINYILVYLNALSCT-QKR-LL--YIIIAENMISSFIGY---INTVFT-----------------SNAVK--------------------------------------------------------------------------------------------------------------FKDSERCIEELFIETGLYIS--------------PQGSYKKHTEVMEELIYLYILARL--FKQLL-----------------------

>NP_052021.1_gp132L_Rabbit_fibroma_virus

--------MGNLLYCC-------------------------------------------------------------------------------------------FVVDVTTPSIQRYVNEQVNLDKFLSRRKKKSILLYLLKVIR-------------------------NDKLLTEVYCSKK--------------------YSELYGCKNPNT---------EVVNFINYILIYLNALSYT-QKR-LL--YIIIAENMITSFIEY---INAVFTS----------------SSTVK--------------------------------------------------------------------------------------------------------------FKDSERCIEELFIETGLYIS-------------SSQGYNKQHTEVMEELIYLYILAKL--FKQLL-----------------------

>YP_009408097.1_Immunoprevalent_protein_Eptesipox_virus

--------MGNSTSVS--------------------------------------------------------------------------KSCNNAVTHVSANSIRQHILFNNFETLHKDIQSKIDLVNTFT-PQTKNLIFRNLLIVI--TNSYHLQNL--------------LDALEQLEPMYVT-----------------DAYSEAILNEIGLCD---KGIPNLSSIHFMIYLVSGLTKLTTK-QSKILM--EIVTDAKIFCHHVNV---LEYIIKK----------------NVEKL-ETVTSTLLE----------------------------------------------------------------------------------------------------KYTKLPLEVTLFKESGLKIQ-------------GNTYIWDPEHKKSICN--LYTVIKI--MSYIM-----------------------

>MN240300.1:c166555-165827_Alaskapox_virus_complete_genome

--------MGNKNGKP--------------------------------------------FTENIQFLKDKRDSFKRGTWATSSFREKSHATIQRFSSLRRENVKVDQHD--KYMELKREIYAIIQKSSSID-INKRTKLLSNIKMMM--INPFMIEGLMTS-----------LENLDPDNKMSYS--------------------SVMILGEFDIIN----ISDNETAFEFINNLLKSLLLLNNR-QLK-LL--EYSISNDLLYAHINA---LEYIIKN----------------TFNVP-ERQL--ILR----------------------------------------------------------------------------------------------------GQYLTPIFSDLLKYAGITIK-------------SNILMWNKKFIKPVSD--LYTSMRL--LHCVTVX---------------------

>AY009089.1:c154402-154034_Camelpox_virus_CMS_complete_genome

--------------------------------------------------------------------------------------------------------------------------MMINPF-----------MIEGLMTSL--------------------------ESLDPDNKMSYS--------------------SMMILGEFDIIN----ISDNEVAFEFIHSLLKSLLLLNTS-QLK-LL--EYSISNDLLYTHINA---LEYIIKN----------------TFNVP-ERQL--ILR----------------------------------------------------------------------------------------------------GQYLTPIFSDLLKYAGLTIK-------------------------------------X------------------------------

>MK910851.1:c154204-153836_Camelpox_virus_strain_Negev2016_complete_genome

--------------------------------------------------------------------------------------------------------------------------MMINPF-----------IIEGLMTSL--------------------------ESLDPDNKMSYS--------------------SMMILGEFDIIN----ISDNEVAFEFIHSLLKSLLLLNTS-QLK-LL--EYSISNDLLYTHINA---LEYIIKN----------------TFNVP-ERQL--ILR----------------------------------------------------------------------------------------------------GQYLTPIFSDLLKYAGLTIK-------------------------------------X------------------------------

>DQ437594.1:c153307-152573_Taterapox_virus_strain_Dahomey_1968_complete_genome

--------MGNKNIKP------------------------------------------SKENRLSILYKDRMDSFKRGSWVTSSFREKSHATIQRFSSLRREHVKVDHPD--KFLELKREIYAIIQKSSSID-VDKRTKLMSNIKTMM--INPFMIEGLMTS-----------LESLDPDNKMSYS--------------------SVMILGEFDIIN----ISDNEAAFEFINSLLKSLLLLNTS-QLK-LL--EYSISNNLLYTHINA---LEYIIKN----------------TFNVP-ERQL--ILR----------------------------------------------------------------------------------------------------GQYLTPFFSDLLKYAGLTIK-------------SNILMWNKKFIKPVSD--LYTSMRL--LHCVTVX---------------------

>KY358055.1:c145385-144651_Variola_virus_strain_VD21_17th_century_complete_genome

--------MGNKNIKP------------------------------------------SKENRLSILYKDRMDSFKRGSWATSSFREKSHATIQRFSSLRREHVKVDHPD--KFLELKREIYAIIQKSSSID-VDKRTKLMSNIKTMM--INPFMIEGLMTS-----------LESLDPDNKMSYS--------------------SVMILGEFDIIN----ISDNKAAFEFINSLLKSLLLLNTS-QLK-LL--EYSISNDLLYTHINA---LEYIIKN----------------TFNVP-ERQL--ILR----------------------------------------------------------------------------------------------------CQYLTPIFSDLLKYAGLTIK-------------SNILMWNKKFIKPVSD--LYTSMRL--LHCVTVX---------------------

>DQ441432.1:c146026-145292_Variola_virus_strain_Korea_1947_(Lee_Masterseed)_complete_genome

--------MGNKNIKP------------------------------------------SKENRLSILYKDRMDSFKRGSWATSSFREKSHATIQRFSSLRREHVKVDHPD--KFLELKREIYAIIQKSSSID-VDKRTKLMSNIKTMM--INPFMIEGLMTS-----------LESLDPDNKMSYS--------------------SVMILGEFDIIN----ISDNKAAFEFINSVLKSLLLLNTS-QLK-LL--EYSISNDLLYTHINA---LEYIIKN----------------TFNVP-ERQL--ILR----------------------------------------------------------------------------------------------------CQYLTPIFSDLLKYAGLTIK-------------SNILMWNKKFIKPVSD--LYTSMQL--LHCVTVX---------------------

>DQ441437.1:c146904-146170_Variola_virus_strain_Sierra_Leone_1969_(V68-258)_complete_genome

--------MGNKNIKP------------------------------------------SKENRLSILYKDRMDSFKRGSWATSSFREKSHATIQRFSSLRREHVKVDHPD--KFLELKREIYAIIQKSSSID-VDKRTKLMSNIKTMM--INPFMIEGLMTS-----------LESLDPDNKMSYS--------------------SVMILGEFDIIN----ISDNKAAFEFINSLLKSLLLLNTS-QLK-LL--EYSISNDLLYTHINA---LEYIIKN----------------TFNVP-ERQL--ILR----------------------------------------------------------------------------------------------------CQYLTPIFSDLLKYAGLTIK-------------SNILMWNKKFIKPVSD--LYTSMQL--LHCVTVX---------------------

>MH607143.1:c167386-166652_Akhmeta_virus_isolate_Vani_2010_complete_genome

--------MGNKHIKP------------------------------------------SKENRLSILYKDRMDSFKRGSWATSSFREKSHATIQRFSSLRREHVKVDHPD--KFLELKREIYVIIQKSASID-VDKRTKLMSNIKTMM--INPFMIEGLMSS-----------LENLDPDNKMSYS--------------------SVMILGEFDIIN----ISDNEKAFEFINSLLKSLLLLNTR-QLK-LL--EYSISNDLLYAHINA---LEYIIKN----------------TFNVP-ERQL--ILR----------------------------------------------------------------------------------------------------GQYLTPIFSDLLNYAGLTIK-------------SNILMWNKKFIKPVSD--LYTSMRL--LHCVTVX---------------------

>MN912466.1:c156293-155559_Ectromelia_virus_WH_complete_genome

--------MGNKNIKP------------------------------------------SKENRLSILSKDKMDSFKRGSCATSSFREKSHATIQRFSSLRREHIKVDHPD--KFLVLKREIYEIIQKSSSID-VDKRTKLMSNIKTMM--INPFMIEGLMTS-----------LENLDPDNKMSYS--------------------SAMILGEFDIIN----ISDNEATFEFINSLLKSLLLLNTR-QLK-LL--EYSISNDLLYANINA---LEYIIKN----------------TFNVP-ERQL--ILR----------------------------------------------------------------------------------------------------GQYLTPIFSDLLKYAGLTIK-------------SNILMWNKKFIKPVSD--LYTSMRL--LHCVTVX---------------------

>KC813500.1:c153312-152578_Cowpox_virus_strain_MonKre08/4_complete_genome

--------MGNKNIKP------------------------------------------SKENRLSILSKDKMDSFKRGSWATSSFREKSHATIQRFSSLRREHIKVDHPD--KFLELKREIYEIIQKSSSID-VDKRTKLMSNIKTMM--INPFMIEGLMTS-----------LENLDPDNKMSYS--------------------SVMILGEFDIIN----ISDNEAAFEFINSLLKSLLLLNTR-QLK-LL--EYSISNDLLYAHINA---LEYIIKN----------------TFNVP-ERQL--ILR----------------------------------------------------------------------------------------------------GQYLTPIFSDLLKYAGLTIK-------------SNILMWNKKFIKPVSD--LYTSMRL--LHCVTVX---------------------

>NC_055231.1:c163765-163031_Orthopoxvirus_Abatino_complete_genome

--------MGNKHIKP------------------------------------------SKENRLSILSKDKMDSFKRGSWATSSFREKSHATIQRFSSLRREHVKVDHPD--KFLELKREIYEIIQKSSSID-VDKRTKLMSNIKTMM--INPFMIESLMTS-----------LENLDPDNKMSYS--------------------SVMILGEFDIIN----ISDNEAAFEFINSLLKSLLLLNTR-QLK-LL--EYSISNDLLYAHINA---LEYIIKN----------------TFNVP-ERQL--ILR----------------------------------------------------------------------------------------------------GQYLTPIFSDLLKYAGLTIK-------------SNILMWNKKFIKPVSD--LYTSMRL--LHCVTVX---------------------

>AY243312.1:c154675-153917_Vaccinia_virus_WR_complete_genome

--------MGNKNIKP------------------------------------------SKENRLSILSKDKMDSFKRGSWATSSFREKSRATIQRFSSLRREHIKVDHPD--KFLELKRGIYKIIQKSSSID-VDKRTKLMSNIKTMM--INPFMIEGLMTS-----------LENLDPDNKMSYS--------------------SVMILGEFDIIN----ISDNEAAFEFINSLLKSLLLLNTR-QLK-LL--EYSISNDLLYAHINA---LEYIIKN----------------TFNVP-ERQL--ILR----------------------------------------------------------------------------------------------------GQYLTPIFSDLLKYAGLTIK-------------SNILMWNKQFIKPVSD--LYTSMRL--LHCVTESYKVIGMGX-------------

>MG599038.1:c157906-157148_Buffalopox_virus_isolate_Karachi_2005_complete_genome

--------MGNKNIKP------------------------------------------SKENRLSILSKDKMDSFKRGSWATSSFREKSRATIQRFSSLRREHIKVDHPD--KFLELKRGIYEIIQKSSSID-VDKRTKLMSNIKTMM--INPFMIEGLMTS-----------LENLDPDNKMSYS--------------------SVMILGEFDIIN----ISDNEAAFEFINSLLKSLLLLNTR-QLK-LL--EYSISNDLLYAHINA---LEYIIKN----------------TFNVP-ERQL--ILR----------------------------------------------------------------------------------------------------GQYLTPIFSDLLKYAGLTIK-------------SNILMWNKQFIKPVSD--LYTSMRL--LHCVTESYKVIGMGX-------------

>KY349117.1:c166272-165538_Horsepox_virus_strain_MNR_complete_genome

--------MGNKNIKP------------------------------------------SKENRLSILSKDKMDSFKRGSWATSSFREKSRATIQRFSSLRREHIKVDHPD--KFLELKRGIYEIIQKSSSID-VDKRTKLMSNIKTMM--INPFMIEGLMTS-----------LENLDPDNKISYS--------------------SVMILGEFDIIN----ISDNEAAFEFINSLLKSLLLLNTR-QLK-LL--EYSISNDLLYAHINA---LEYIIKN----------------TFNVP-ERQL--ILR----------------------------------------------------------------------------------------------------GQYLTPIFSDLLKYAGLTIK-------------SNILMWNKQFIKPVSD--LYTSIRL--LHCVTVX---------------------

>M35027.1:c153690-152956_Vaccinia_virus_Copenhagen_complete_genome

--------MGNKNIKP------------------------------------------SKENRLSILSKDKMDSFKRGSWATSSFREKSRATIQRFSSLRREHIKVDHPD--KFLELKRGIYEIIQKSSSID-VDKRTKLMSNIKTMM--INPFMIEGLMTS-----------LENLDPDNKMSYS--------------------SVMILGEFDIIN----ISDNEAAFEFINSLLKSLLLLNTR-QLK-LL--EYSISNDLLYAHINA---LEYIIKN----------------TFNVP-ERQL--ILR----------------------------------------------------------------------------------------------------GQYLTPIFSDLLKYAGLTIK-------------SNILMWNKQFIKPVSD--LYTSIRL--LYCVTVX---------------------

>AY484669.1:c159516-158782_Rabbitpox_virus_complete_genome

--------MGNKNIKP------------------------------------------SKENRLSILSKDKMDSFKRGSWATSSFREKSRATIQRFSSLRREHIKVDHPD--KFLELKRGIYEIIQKSSSID-VDKRTKLMSNIKTMM--INPFMIEGLMTS-----------LENLDPDNKMSYS--------------------SVMILGEFDIIN----ISDNEAAFEFINSLLKSLLLLNTR-QLK-LL--EYSISNDLLYAHINA---LEYIIKN----------------TFNVP-ERQL--ILR----------------------------------------------------------------------------------------------------GQYLTPIFSDLLKYAGLTIK-------------SNILMWNKQFIKPVSD--LYTSIRL--LHCVTVX---------------------

>KU749309.1:c145369-144644_Raccoonpox_virus_strain_85A_complete_genome

--------MGNNIKSS-------------------------------------------KENRQSGLFKDRMDSFKRGSWATSSFREKSVPTIHKFSSLRRERVTVQPDS--KFIELKREIYAIINKSSSID-IDKRIILISNIKKMM--VNPFMIEGLMNS-----------LEKMDPEDMISYS--------------------SVMILGEFDINM------NDAPSFQFIISLLKSLHALNNK-QLK-LL--EYSIGNDLLYSQVTA---LEYVIKN----------------TFDVP-ERQL--ILR----------------------------------------------------------------------------------------------------GKYLTPIFNDLLKYSGLTIK-------------SNILMWNKKFIKPVSD--LYIAIRL--LYCMTIX---------------------

>KU749310.1:c167631-166873_Skunkpox_virus_strain_WA_complete_genome

--------MGNNIKPS-----------------------------------KEDKQLSSKGNRQSILFKDRMDSFKRGSWATSSFREKSVTTIQKFSSLRREHTKIEQPDS-KFIELKREIYEIINKSSSID-IDKRLMLIYNIKKMM--VQPFMLEGLMNS-----------LEKMDPEDMISYS--------------------SVMILGEFDIVN----IYDNKSTFQFINGLLKSLHALNSK-QLK-LL--EYSISNDLLYNQVTA---LEYVIKN----------------TFDVP-ERQL--ILR----------------------------------------------------------------------------------------------------GQYLTPIFNDLLKYSGLTIK-------------SNILMWNKKFIKPVSD--LYTAIRL--LYCVTIX---------------------

>KU749311.1:c169057-168281_Volepox_virus_strain_CA_complete_genome

--------MGNNIKPS----------------------------KENRQSLSKENRQSLKKNRQSFLFNDGMDTFKRGSWATSSFREKSASTIHKFSSLRREHTKIDQPDS-KFIELKREIYEIINKSSSID-IDKRIMLVYNIKKMM--VNPFMIEGLMNS-----------LEKMDPEDVISYS--------------------SVMIFGEFDMVN-----IHNKSAFQFINSLLKSLHALNNK-QLK-LL--EYSISNDLLYNQVTA---LEYVIKN----------------TFDVP-ERQL--ILR----------------------------------------------------------------------------------------------------GQYLTPIFNDLLKYSGLTIK-------------SNILMWNKKFIKPISD--LYTAIRL--LYCVTVX---------------------

>HQ849551.1:c134784-134035_Yoka_poxvirus_strain_DakArB_4268_complete_genome

--------MGNKNSDP----------------------------------------VFTSTSCNNTTSLKRGQSLHLHRDLDKLSLNENTLFNRNFSFRRVNSMRIKPSNYSKLIELKKEIFETIKKANDMD-IDKRIKLLHNIKSII--TNPFVLKGLIES-----------LETFESVDGIKYS--------------------SVMILGEFNILD----QYTGTSTFQFVIDLLKSLYVLNNK-QQK-II--EYAINNDMLYEQIEM---IEYIMTN----------------VLDID---NYHFILK----------------------------------------------------------------------------------------------------GKYVTPLFVELLKNTGINVI------------SNNIIIWNKKYTKNISN--LYKAIRL--LHSITVX---------------------

>MF001305.1:c159640-158771_NY_014_poxvirus_strain_2013_complete_genome

--------MGNKHSEPVFSESTISYTKRSMRVPCNEKPSTELKTGFNRLSLREKTVSSLQRGFNRLSLNEKTFSLSRGFNNRLSLNEKTFSLQRGFS-LRRFNSVRGESSNSKLMELKKEIYETIQQANDMD-IDKRIKLLYNIKSMM--GNSFMLKGLIDS-----------LETLEPTDGISYS--------------------SVMILGEFSILD--QYTDGGKSTFKFVINLLKSLDSLNHH-QQK-LI--EYAVNNDILYDQITM---IEYIMKN----------------NLDME-NRQF--ILK----------------------------------------------------------------------------------------------------GKYTTPLFIDLLKHTGINVI-------------SNVLIWNKKYTKSISN--LYTAMRL--LYSITVX---------------------

>MF001304.1:c160603-159728_Murmansk_poxvirus_strain_LEIV-11411_complete_genome

-----MGNKHSEPVFSASTISYTKRSSIRRVPCNENPSTELKTGEFNRLSLREKTVSSLQRGFNRLSLNEKTFSLHRGFNNRLSLNEKTFSLQRGFSLRRFNSVRGESSS--KLMELKKEIHETIQQANNMD-IDKRIKLLYNIKSMM--GNSFMLKGLIDS-----------LETLEPTDGISYS--------------------SVMILGEFSILD--QYTDGGKSTFKFVINLLKSLDSLNHH-QQK-LI--EYAVNNDMLYDQITM---LEYIMKN----------------NLDME-NRQF--ILK----------------------------------------------------------------------------------------------------GKYTTPLFIDLLKHTGINVI-------------SNVLMWNKKYTKSISN--LYTAMRL--LYSITVX---------------------

>ref|XP_032903802.1|_location_chromosome_chromosome_2_completeness_complete_organism_Amblyraja_radiata_isolate_CabotCenter1_gcode_1_chromosome_2_sex_male_tissue_type_testis_liver_country_USA:_Gulf_ofa

---PLSCVDGAHKCQL----------------------------------------------------------------------------------SALEMDVPSETSLIILKPAIEEANQQFQPFHELA-EEKCLQLFRLYCEFL--YHEEVISFLENALDE--------LSSTEQPNLLGLQ--ELDSTQAQRVKELLQILGYSCPNEQGSLKK----DITTPEILTAAFYLVSALAGMSEE-SLA-VL--GICCETQILPTLHYL---INNVPDD----------------GIVPV-DNPNMLPLQ----------------------------------------------------------------------------------------------------EEDNFYIAHRLFALSNICLE-----------VRETAIKVITDDQPGMAPVLLCIVLQG--FAILSGIKRNTSDPKTQT----------

>ref|XP_031796450.1|_location_chromosome_chromosome_5_completeness_complete_organism_Sarcophilus_harrisii_gcode_1_chromosome_5_cell_line_91H_gasdermin-E_Sarcophilus_harrisii

-----DSVDGKSEAVK---------------------------------------------------------------------------------------LTPVEGPLSALKHGMLVLERNFRPFVELS-GHHRAALCEILWEVL--LDDELVVALDTVLSDV-------LAGGRPRLWALPG-----LSPGQQQQLAAFLGLAGLHVQEDSRVR--RDARAPRELLSTAHFLVSALAEMPDD-TVA-LM--GVCCQLHLIPTLCHL---PQVTSAD----------------GVSEL-GDPSLAPLS----------------------------------------------------------------------------------------------------DAGSFEVVQKLFALSHISLE-----------RREAHVEAVIMKRPQYLPLVLYITLHG--LCALEGKAE-------------------

>ref|NP_061239.1|_chromosome_6_organism_Mus_musculus_strain_C57BL/6_gcode_1_chromosome_6_map_6_gasdermin-E_precursor_Mus_musculus

--------DGGQGISS------------------------------------------------------------------------------------------QDGPLRVVKQATLHLERSFHPFAVLP-AQQQRALFCVLQKIL--FDEELLRALEQVCDDVA------GGLWSSQAVLAME--ELTDSQQQDLTAFLQLVGYRIQGEHPGPQD----EVSNQKLFATAYFLVSALAEMPDN-ATV-FL--GTCCKLHVISSLCCL---LHALSDD----------------SVCDF-HNPTLAPLR----------------------------------------------------------------------------------------------------DTERFGIVQRLFASADIALE-----------RMQFSAKATILKDSCIFPLILHITLSG--LSTLSKEHEEELCQSGHATGQD------

>ref|XP_021046698.1|_location_chromosome_chromosome_2_completeness_complete_organism_Mus_pahari_gcode_1_chromosome_2_sex_female_tissue_type_tail_gasdermin-E_Mus_pahari

--------DGGQGISS------------------------------------------------------------------------------------------QDGPLRVVKQATLHLERSFHPFAVLP-AQQQRALFCVLQKIL--FDEELLRALEQVCDDVA------GGLWSSQAISAME--ELTDSQQQDLTAFLQLVGYRIQGEHPGPQD----AVSNQKLFATAYFLVSALAEMPDN-ATV-FL--GTCCKLHVISSLCCL---LHALSDD----------------SVCDF-QDPALAPLR----------------------------------------------------------------------------------------------------DTERFGIVQRLFASADIALE-----------RMQFSAKATILKDSCIFPLILHITLSG--LSTLSKEHEEQLCQSEGHATGQD-----

>ref|NP_001180041.1|_chromosome_4_organism_Bos_taurus_breed_Hereford_gcode_1_chromosome_4_map_4_gasdermin-E_Bos_taurus

--------DAGQGLPA------------------------------------------------------------------------------------------QDKPLSVLKQATLLLERNFHPFMELP-EQQQIALNDVLQAVL--LDEELLVVLEQVCDDI-------VSSLSPSLVALGE---LKPSQKHNLTTFLRLVGCSVQGERLGSQD----VVDNQKLFSTAFFLVSALAEMPDN-AAA-LL--GTCCKLQIIPLLYHL---LRALSHD----------------GVSDL-EDPALAPLK----------------------------------------------------------------------------------------------------DREKFGIVQRLFAATDINLE-----------RMQSSVKAASREDPNVLPLILYISLSG--LCALGRAQ--------------------

>ref|XP_024418892.1|_chromosome_Unknown_completeness_complete_organism_Desmodus_rotundus_isolate_DRU21DN04_gcode_1_chromosome_Unknown_sex_male_tissue_type_muscle/skin_sample_dev_stage_adult_country_Us

--------DAGQGPSA------------------------------------------------------------------------------------------PDEPLSVLKQATLPLERAFHPFGQLP-EQEQTALSGVLQVVL--FDEELLVVLEQVCDDM-------VGGLPPSLAVLGE---LKPPQKQNLMAFLQLVGCSMQGECLGPQG----VVSNQKLFSTAYFLVSALAEMPDN-AAA-LL--GTCCKLQIIPALCHL---LRALSAG----------------GEPDL-EDTALAPLR----------------------------------------------------------------------------------------------------DTERFGIVQCLLATASLDLE-----------RRQSSVKAVTCQVPNVSPLVLYVSLSG--LHALGRAHQ-------------------

>ref|XP_006912063.1|_chromosome_Unknown_completeness_complete_organism_Pteropus_alecto_gcode_1_chromosome_Unknown_sex_male_tissue_type_kidney_country_Australia:_Anglican_Church_Grammar_School_East_Bro

--------DAWQGPPA------------------------------------------------------------------------------------------SDKPLSVLKEVTLPLEKSFRPFAELP-EQQQTALSGILQAVL--FDDELLLALEQVCDDV-------FSDLSPPRVIPGE---LKPSQQQDLMAFLHLVGYSMQSGCPGPED----AVSNQKLFSTAYFLVSALAEMPDN-AAA-LL--GTCCKLQIIPTLCHL---LHVLSAD----------------GESDL-EDAALAPLR----------------------------------------------------------------------------------------------------DTERFEIVQRLLAAADINLE-----------RQQSSVKAVTCQVPNVSPLVLYISLNG--LWALGRMHQ-------------------

>ref|XP_016015046.2|_chromosome_Unknown_completeness_complete_organism_Rousettus_aegyptiacus_isolate_mRouAeg1_gcode_1_chromosome_Unknown_sex_male_tissue_type_muscle_dev_stage_adult_country_USA:_Berkes

--------DAWQRPSA------------------------------------------------------------------------------------------SDKPLSVLKQVSLPLERSFRPFAELP-QQQQTALSGILQAVL--FDEELLLALEQVCDDV-------FSGLSPPRAMPGE---LKPSQQQDLAAFLRLVGYSVQSGCPGPED----AVSNQKLFSTAYFLVSALAEMPDN-AAA-LL--GTCCKLQMIPTLCHL---LRALSAD----------------GESDL-EDAALAPLR----------------------------------------------------------------------------------------------------DTERFEIVQRLLAAADIRLE-----------RRQSSVKAVTYPVPDVSQLVLYVSLNG--LWALARMHQ-------------------

>ref|XP_004626528.1|_chromosome_Unknown_completeness_complete_organism_Octodon_degus_isolate_3935_gcode_1_chromosome_Unknown_sex_female_gasdermin-E_Octodon_degus

--------DAKHGMLS------------------------------------------------------------------------------------------KDGLLSVLKQATLPLERNFHPFVELP-AQQQQALHNLLQAVL--LDEELLVVLEQVCDDMA------GSVWSPQTALAMR--ALKAPQQQDLTAFLQLVGCSIQGECPSPED----EVSNQKLFATAYFLVSALAEMPDN-AAA-LL--GICCKLQIIPTLYHM---LRALSDD----------------GVCDL-EDPTLAPLR----------------------------------------------------------------------------------------------------DTERFGIVQRLFVATDINLE-----------QEQASVKAVILKDPHIFPLILYITLNG--LCALGREH--------------------

>ref|XP_001096213.2|_location_chromosome_chromosome_3_completeness_complete_organism_Macaca_mulatta_isolate_AG07107_bio_material_Coriell:AG07107_gcode_1_chromosome_3_sex_female_tissue_type_fibroblasta

-----DAAHGISSQDG------------------------------------------------------------------------------PLSVLKQVTPLLERNFHPFVELPEPQQTALSDIFQAVLFDDELLMVLEPVCDDLVSGLSPPMAVLGEL-----------KLQQQQDLEAFLQ-----------------LVGCSLHGGCPGPED-----TGSKQLFMTAYFLVSALSEMPDN-AAA-LL--GTCCKLQIIPTLCHL---LRVLSDD----------------GVSDL-EDPTLAPLK----------------------------------------------------------------------------------------------------DTERFGIVQRLFASADISLE-----------RLKSSVKAVILKDSKVFP-LLCIALNG--LCALGREHS-------------------

>ref|NP_001120925.1|_chromosome_7_organism_Homo_sapiens_gcode_1_chromosome_7_map_7p15.3_gasdermin-E_isoform_a_Homo_sapiens

-----DAAHGISSQDG------------------------------------------------------------------------------PLSVLKQATLLLERNFHPFAELPEPQQTALSDIFQAVLFDDELLMVLEPVCDDLVSGLSPTVAVLGEL-----------KPRQQQDLVAFLQ-----------------LVGCSLQGGCPGPED-----AGSKQLFMTAYFLVSALAEMPDS-AAA-LL--GTCCKLQIIPTLCHL---LRALSDD----------------GVSDL-EDPTLTPLK----------------------------------------------------------------------------------------------------DTERFGIVQRLFASADISLE-----------RLKSSVKAVILKDSKVFPLLLCITLNG--LCALGREHS-------------------

>ref|XP_001159685.3|_location_chromosome_chromosome_7_completeness_complete_organism_Pan_troglodytes_isolate_Yerkes_chimp_pedigree_*C0471_(Clint)_gcode_1_chromosome_7_sex_male_tissue_type_blood_dev_ss

-----DAAHGISSQDG------------------------------------------------------------------------------PLSVLKQATLLLERNFHPFAELPEPQQTALSDIFQAVLFDDELLMVLEPVCDDLVSGLSPTVAVLGEL-----------KPRQQQDLVAFLQ-----------------LVGCSLQGGCPGPED-----AGSKQLFMTAYFLVSALAEMPDN-AAA-LL--GTCCKLQIIPTLCHL---LRALSDD----------------GVSDL-EDPILTPLK----------------------------------------------------------------------------------------------------DAERFGIVQRLFASADISLE-----------RLKSSVKAVILKDSKVFPLLLCITLNG--LCALGREHS-------------------

>ref|XP_029444983.1|_location_chromosome_chromosome_2_completeness_complete_organism_Rhinatrema_bivittatum_gcode_1_chromosome_2_gasdermin-E_isoform_X1_Rhinatrema_bivittatum

-YSWDVVDSGDRSMMN-------------------------------------------------------------------------------------RNVVPSDAPLSLLKQDVLQQTKIFQSFLELP-EEKRSVLNKLLCEIL--SHGETLTLLEDV-----------LDDICTEHKPNLTALNELKVEQQQNIKDLWPLLGLGLENELLLQP---EKLQEKELLLATHVLVSALEEMGDS-PLA-IL--RACCELHLMPVLCHL---LNSLSDD----------------GTSMR--DSLLSIFN----------------------------------------------------------------------------------------------------DKGRFQIAQKLFSLSNISLE-----------LTEDNIRAVTKSEPGFLPLILSIILSG--FHVLAGTF--------------------

>ref|XP_027689547.1|_location_chromosome_chromosome_2_completeness_complete_organism_Chelonia_mydas_isolate_rCheMyd1_gcode_1_chromosome_2_sex_male_tissue_type_blood_dev_stage_adult_country_Israel:_Mes

---FLYQPDAVDSDRR----------------------------------------------------------------------------------SGDRNIVPSDASLSVLKQDLLLLKMQFQPFVKLP-EDKQGALNKIFYELL--HHEEIVIALEEVLDS--------IFTGEEPDFTDLK--ELKPPQQQNITDFLQLVGYSLQNELLQKDQ--LH---YTGLFSAAHILISAVAELSDY-TLA-LL--RACCDIQIVPILCYL---PNVASAD-----------G----TLTLR-DPALA-PLT----------------------------------------------------------------------------------------------------DTGRFHIVQRLFALSNINLE-----------MMESSMKAVTMKEPSFFPFILYIALYG--FYALGGTI--------------------

>ref|XP_023962559.1|_location_chromosome_chromosome_2_completeness_complete_organism_Chrysemys_picta_bellii_isolate_RCT428_sub_species_bellii_gcode_1_chromosome_2_sex_female_country_USA:_Grant_Co._...

---FLYQPDAVDSDRH----------------------------------------------------------------------------------SGDRNIIPSGASLSVLKQDLLLLKMQFQPFVKLP-EDKQGALNKIFYEFL--HHEEMVTALEEVLDS--------ICTGEEPDFTDLK---ELKPPQQQNITDFLQLVGYSLQNELLLQN---DQLHYKGLFSAAHILISAVAELSDY-TLA-LL--RACCDIQIIPALCCL---PNVASAD----------------GTLTL-RDPALAPLT----------------------------------------------------------------------------------------------------DTGRFDIVQRLFALSNINLE-----------MMESSVKAVTMKEPSFFPFILYIALYG--FYALGGTI--------------------

>ref|NP_001006361.1|_chromosome_2_organism_Gallus_gallus_breed_Leghorn_gcode_1_chromosome_2_map_2_gasdermin-E_Gallus_gallus

---FLFHPDAVDNGMY----------------------------------------------------------------------------------SGAENPVPSHASLSVLKKDLSQLKAQFQPFVKLP-EDKQRALYKSLCELL--LHEETLTALEDVLDALCTGDKPDLKELKPAQQQDLA---------------DFLQLVGCRVQDELLLQ--NYHLQDEELLSAAHLLISAISELSDY-TLV-LL--RACSDLQVVPALCCL---PYIASAD-----------G----TLALS-SPSVA-TLT----------------------------------------------------------------------------------------------------DRRRFDVAQRLFASANINLE-----------KTETSVKAVTMREPRFFPLVLYVALCG--LHSLGGTVEELF----------------

>ref|XP_009324004.1|_chromosome_Unknown_completeness_complete_organism_Pygoscelis_adeliae_isolate_BGI_AS28_gcode_1_sex_male_country_Antarctica_chromosome_Unknown_PREDICTED:_non-syndromic_hearing_impae

---FLYQPDAVDNEMY----------------------------------------------------------------------------------SGAKNRIPSDASLSVLKQDLSWLKTQFQPFVKLP-EDKQRALYKTLCELL--LHEEMVAALEDLLDDICTGDKPDLKELKPAQQQDLL--------------GFLQLLGCSLQSELLLQK---YQPQDEELLSAAHLLISAISELPDY-TLV-LL--RACCDLQVVPALCCL---PNIASAD----------------GTLAL-SSPLVATLT----------------------------------------------------------------------------------------------------DRGRFDVVQRLFASSNITLE-----------MTVSSVKAVTMKEPRFFPLVLYVALYG--FYALGGNV--------------------

>ref|XP_002933445.2|_location_chromosome_chromosome_6_completeness_complete_organism_Xenopus_tropicalis_strain_Nigerian_gcode_1_chromosome_6_sex_female_tissue_type_liver_and_blood_dev_stage_adult_nots

VLFDWDVVDGSKEAFV-------------------------------------------------------------------------------------RKCILRSAPLCLLREGIAAHEKHFSAWKQLP-DVQCLELYTLLCQIL--YDGQALSKLHAVVED--------LCSKRKPTQAAFD---------ELMPSQRIIAENILYLSGYDMPNGKFLHAANRELLVALHILTSALNELSDS-ALA-VL--GTCCELQLLPVFSAL---MNMSSDE----------------GLCST-TEPALMDFL----------------------------------------------------------------------------------------------------DQERFYVSQKLFALFNIELE-----------IKEDFIYAATAEDPGFLPLILFIVITG--LQLLKRD---------------------

>ref|XP_040208817.1|_location_chromosome_chromosome_5_completeness_complete_organism_Rana_temporaria_gcode_1_chromosome_5_gasdermin-E_Rana_temporaria

----FDWDMVDLSRNI----------------------------------------------------------------------------------SVDGKAVPSNYSLSAIKSDILEITKPFSVLQDLP-EAHRGEIYNGLFEIL--HDGQTVTQLQAVVEE--------ICLEKRPGLTFLS-------ELKSPERAWVQKILHLIGYDMLNQK--LIEPFKKDLLSAVHLLTSALDEMPDT-ALA-IL--GACCKLHLLPALWAL---TNITSDE----------------GLCSR-TEPALTDLI----------------------------------------------------------------------------------------------------HQGRFQIAQSLFSFFNMRLE-----------LNEKNIAVTSEDPSLLPH-ILYIAISG--FHALKNNLKI------------------

>XP_032891479.1_gasdermin-A_Amblyraja_radiata

----------------------------------------------------------------------------------------------------------SLFCQDTANVNGDEVFEICCELQCLD-KGLKQRLLGLVCQII--EDSELLSILSDVLCEACAGTDYTLSELEKLDKKRRE----------------CGENLLAIWSEDQLKN-----AVEDNLLKAVSILFAALEDLPPT-TLP-VL--IQSLKMQILPQQLTL---VTGILKD----------------LDHSQVDQALKVETESF--------------------------------------------------------------------------------------------------TEDAFGITAEMLTDVGLHLE---------------RDSVQKTREPSLHE--LSVALYC--LNALSSN---------------------

>ref|XP_029428328.1|_location_chromosome_chromosome_12_completeness_complete_organism_Rhinatrema_bivittatum_gcode_1_chromosome_12_gasdermin-A_Rhinatrema_bivittatum

-----------------------------------------------------------------------------------------------------DYLRILENFQEGFPAVMAQIEKECVEFTLLS-PEVAEQFLAAFLLLI--KDPKTSADVESRLEQS-------LYCSARESKAHPE------------------PSIARLLGYLGLSS----RPVVIPLVRAVLFFLDAVSELDED-GTSLLV--ESVERQDTGQQLKRV---QKVLEEE----------------LFSVR-QGSLA--------------------------------------------------------------------------------------------------------GDEAAG---------GVSPE-----------------GWSPAVQSCAMA--LYAALYA--LHVLSSC---------------------

>ref|XP_015154998.2|_location_chromosome_chromosome_27_completeness_complete_organism_Gallus_gallus_isolate_bGalGal1_gcode_1_chromosome_27_sex_female_tissue_type_blood_country_USA:_Fayetteville_lat_ls

--------DGFAHTEV--------------------------------------------------------------------------------------------------EALEEEVKEKCGILCQLA-GELNAIVLKTIKAVM--RDRNLLEELSGKM----------EAVLDDPDNCELT---------------TQSPDLEVLLSTLKGSP----RCDLLSLTEAISYTLDAFSELTED-QLL-LL--LESLEVEIVPQQLKL---VRSILEQ----------------SAELGAAAGLLSSFS----------------------------------------------------------------------------------------------------EQQQQQLTTAVLELSGVELQ------------GSGAAVCTEESFPSLSA--LYAALHA--LHCLVGAGGRSARAD-------------

>ref|NP_081236.1|_chromosome_15_organism_Mus_musculus_strain_C57BL/6_gcode_1_chromosome_15_map_15_gasdermin-D_Mus_musculus

--------DGIDEEEL--------------------------------------------------------------------------------------------IEAADFQGLYAEVKACSSELESLE-MELRQQILVNIGKIL--QDQPSMEALEASLGQG-------LCSGGQVEPLDGP--------------------AGCILECLVLDS----GELVPELAAPIFYLLGALAVLSET-QQQ-LL--AKALETTVLSKQLEL---VKHVLEQ--STPWQEQS------SVSLP-TVLLGD-------------------------------------------------------------------------------------------------------CWDEKNPTWVLLEECGLRLQ-----------VESPQVHWEPTSLIPTSA--LYASLFL--LSSLGQKPC-------------------

>ref|XP_023104116.1|_location_chromosome_chromosome_F2_completeness_complete_organism_Felis_catus_isolate_Cinnamon_breed_Abyssinian_gcode_1_chromosome_F2_sex_female_gasdermin-D_Felis_catus

--------DGDIEDQM--------------------------------------------------------------------------------------------PVTEDFQGLKVEVSVHADGLKGLS-GELCGQILAGLMKVL--REEPALESLQEELEQGL------CCGWVASPDAPGG----------------------AILECLVQSS----GKVEEELARPILYLVQALTELNET-QRV-LL--AEALETGDLSGQSRL---VQSVLEQ--SSPWKEHR------AVSLP-QELLGSSWDSEAPAWVLLEECGLELRVDVPQVHWQPDAQGRTSALYACLVLLPHLSQDQLNETQRVLLAEALETGDLSGQSRLVQSVLEQSSPWKEHRAVSLPQELLGSSWDSEAPAWVLLEECGLELR-----------VDVPQVHWQPDAQGRTSA--LYACLVL--LPHLSQDRA-------------------

>ref|XP_015001615.2|_location_chromosome_chromosome_8_completeness_complete_organism_Macaca_mulatta_isolate_AG07107_bio_material_Coriell:AG07107_gcode_1_chromosome_8_sex_female_tissue_type_fibroblasta

-----------------------------------------------------------------------------------------------------DGVPAEGTFTEDFQGLREEVEAISKELELLD-RELCQLLLEGLEGVL--RDQLALRALEEALEQ--------GLSLGPVESLDGP--------------------AGAVLECLVLES----RMLVPELAVPVVYLLGALTMLSET-QHK-LL--AEALESQTLLGPLEL---VGSLLEQ--SAPWQERS------TVSLP-PGLLG---S----------------------------------------------------------------------------------------------------SWGEGAPAWVLLEECGLELG-----------EDTPHVCWEPQAQGRMCA--LYASLAL--LSGLSQEPH-------------------

>ref|XP_009454389.2|_location_chromosome_chromosome_8_completeness_complete_organism_Pan_troglodytes_isolate_Yerkes_chimp_pedigree_*C0471_(Clint)_gcode_1_chromosome_8_sex_male_tissue_type_blood_dev_ss

-----------------------------------------------------------------------------------------------------DGVPAEGAFTEDFQGLRVEVETISKELERLD-RELCQLLLKGLEGVL--RDQLALRALEEALEQ--------GQSLGPVEPLDGP--------------------AGAVLECLVFSS----GVLVLELAIPVVYLLGALTMLSET-QHK-LL--AEALESQTLLGPLEL---VGSLLEQ--SAPWQERS------TMSLP-PGLLG---S----------------------------------------------------------------------------------------------------SWGEGAPAWVLLDECGLELG-----------EDTPHVCWEPQAQGRMCA--LYASLAL--LSGLSQEPH-------------------

>ref|NP_079012.3|_chromosome_8_organism_Homo_sapiens_gcode_1_chromosome_8_map_8q24.3_gasdermin-D_Homo_sapiens

--------DG---------------------------------------------------------------------------------------------VPAEGAFTEDFQGLRAEVETISKELELLD-RELCQLLLEGLEGVL--RDQLALRALEEALEQ--------GQSLGPVEPLDGP--------------------AGAVLECLVLSS----GMLVPELAIPVVYLLGALTMLSET-QHK-LL--AEALESQTLLGPLEL---VGSLLEQ--SAPWQERS------TMSLP-PGLLGN-------------------------------------------------------------------------------------------------------SWGEGAPAWVLLDECGLELG-----------EDTPHVCWEPQAQGRMCA--LYASLAL--LSGLSQEPH-------------------

>ref|NP_001039625.1|_chromosome_14_organism_Bos_taurus_breed_Hereford_gcode_1_chromosome_14_map_14_gasdermin-D_Bos_taurus

--------DGSTEHWL--------------------------------------------------------------------------------------------ATTDDYQGLRAEVKAWAMGLEGLS-KGLCGQLLGGLGQVL--RDEPALQALEDSLEQG-------LCSGPVEPRDGPV---------------------GAILESLVLPS----GQLETELAGPVFYLLQALAVLSEA-QHV-LL--AEVLEMGALSGTFWL---VESLVEQ--SSPWQEHR------AVSLP-PERLG---D----------------------------------------------------------------------------------------------------SWGSEAPGWALLEACGLEPQ-----------VGTPQVCWEPGARGCACA--LYACLAL--LLRLSQLC--------------------

>ref|XP_024428237.1|_chromosome_Unknown_completeness_complete_organism_Desmodus_rotundus_isolate_DRU21DN04_gcode_1_chromosome_Unknown_sex_male_tissue_type_muscle/skin_sample_dev_stage_adult_country_Us

--------DGLDEDQV--------------------------------------------------------------------------------------------VPTEDFQGLQAEVQVWAGPLESLS-KTLCEQLLRGLAQVL--RDELALQTLEGSLEQG-------LCCGRAEPLQGPA---------------------GHVLECLVLPC----RTLVNELAGPVTYLLGALAALSEA-QRM-LL--AEVLEASALPEPFKL---VEKLLEQ--SSPWQECR------ALSLP-PGLLG---G----------------------------------------------------------------------------------------------------SWGPEVPAWILLEACGLELQ-----------VDAPQVSWKPEAQGCTCA--LYACLAL--LSKLSQLC--------------------

>ref|XP_006913014.1|_chromosome_Unknown_completeness_complete_organism_Pteropus_alecto_gcode_1_chromosome_Unknown_sex_male_tissue_type_kidney_country_Australia:_Anglican_Church_Grammar_School_East_Bro

--------DGPTENCL--------------------------------------------------------------------------------------------VPTEDFQGLQVEVKAWASALENGS-ATLCGQLLGGLGQVL--RDEPALQALEESLEQG-------LCSGRAEPLHGPA---------------------GAVLECLVLHC----RTLVEELARPVFYLLEALAALSET-QHM-LL--AEVLETGALPESVEL---VETLLEQ--STPWQERE------AISLS-PRLLG---S----------------------------------------------------------------------------------------------------NWGSEAPAWVLLEECGLKLQ-----------VGAPQVCWEPEAQGPTCA--LYACLAL--LLKLSQLR--------------------

>ref|XP_036089037.1|_chromosome_Unknown_completeness_complete_organism_Rousettus_aegyptiacus_isolate_mRouAeg1_gcode_1_chromosome_Unknown_sex_male_tissue_type_muscle_dev_stage_adult_country_USA:_Berkes

--------DGPAEHWL--------------------------------------------------------------------------------------------VPTEDFQGLQMEVRAWARALEDAS-ATLCGQLLGALGQVL--WDEPALQALEESLEQG-------LCSGRAEPLHGPA---------------------GAVLECLVLRC----GTLVEELAGPVFYLLGALAALSET-QHV-LL--AKALETGALPEWVKL---VETLLEQ--STPWQERE------AISLP-SELLES-------------------------------------------------------------------------------------------------------SWGSEAPAWVLLEECGLKLQ-----------VGAPQVCWEPEAQGPTCA--LYACLAL--LLRLSQLR--------------------

>ref|XP_031803207.1|_location_chromosome_chromosome_1_completeness_complete_organism_Sarcophilus_harrisii_gcode_1_chromosome_1_cell_line_91H_gasdermin-C_Sarcophilus_harrisii

-----HTEIGLGSMRG-------------------------------------------------------MEEKLEWKDYPKLSLFLETIEETQQGFGWKQQDRNNCQRVRDFQRLLEEVTFQKKDLVRMP-GKQRTTLLCALHDLL--GNRTALQDLEDKL----------LHVLDSGIWGKME------------------GPGNTILSNLQDSS----DSPVNGYITGFLYLLEALAALSDN-QHD-LL--AQSLEKKILSQQLKL---VKSILET----NFNQFHS-----TFILP-PEILSLIDE-------------------------------------------------------------------------------------------------------ELNLSLELVEDCGLQLQ-----------RTEQMLILDPEGKNPLCA--LYGALSC--LLSLSED---------------------

>ref|NP_001039469.1|_chromosome_14_organism_Bos_taurus_breed_L1_Hereford_gcode_1_chromosome_14_map_14_gasdermin-C_Bos_taurus

-----ILSISDDNKRK----------------------------------------------------------------AFFVGKVQRQSAMGTKRRPILPIGRIQEPISRDFKSLQNEVSREIEAVAELP-RDIRDALFHTILAKL--KDQGALQDLTDMLD---------GNLWDHTGSFLSE-----------------------------MRE--DSRNVWLESRPCVIYLLEALLVLSDI-QHE-LL--AWSMEKRILPQQREL---VESILEP--NFRYPWNI------PFTLD-PKLLASL------------------------------------------------------------------------------------------------------QDEGLAVTFGLLQECGLRVA-----------PDNPKGTWDLEAKKPLSA--LYGSLSV--LQQLAEA---------------------

>ref|XP_021073018.1|_location_chromosome_chromosome_17_completeness_complete_organism_Mus_pahari_gcode_1_chromosome_17_sex_female_tissue_type_tail_gasdermin-C_Mus_pahari

---RPMYLSGHFEILR---------------------------------------------------------------------HHEEIIRIGSRIEYISPIGTIEEPTHLDFQCLQNEVSQKTMLLAELS-KDVQEVVFSSFLHMLCEGDSEVLYDLMK------------MLELNQLEHMDGP--------------------GGKILEELQKDR----SPSWIDLKDLILNLLQALMVLSNT-QLS-LL--AQSVEMRLLLQQQEL---VKSILQP--NYKYPWHI------PFTLQ-PQLLA-PLQ----------------------------------------------------------------------------------------------------GEGLAITYE-LLEECGLKME-----------LNNPRSTWHLEVKMPLSA--LYASLSL--LQQLSEA---------------------

>ref|XP_019678683.1|_location_chromosome_chromosome_F2_completeness_complete_organism_Felis_catus_isolate_Cinnamon_breed_Abyssinian_gcode_1_chromosome_F2_sex_female_gasdermin-C_Felis_catus

-----AVNNGPLYTMS------------------------------------------------------------------------VSDVRKPLDLVLHSPGRIQRVLWQDFKQLHEEIFQEMEALAQLS-KDTQDSIFHTILNML--GNREALQDL--------------TDTLDGSPLDLLD------------------DFGGTILNEMQPDT----RDLWIQARFHIIYLLEVIMVLSDT-QHD-LL--AQSMEKRILLQQQKL---VRSILEP-----NFKYCWDI---PFTLK-PELLA-PLQ----------------------------------------------------------------------------------------------------GEDLAITY-GLLEECGLKME-----------LNSPRSTWDLETKQPLSA--LYGTLSL--LQQLTGSPERPGPTPVYAESVPDELGGV

>ref|XP_028708741.1|_location_chromosome_chromosome_8_completeness_complete_organism_Macaca_mulatta_isolate_AG07107_bio_material_Coriell:AG07107_gcode_1_chromosome_8_sex_female_tissue_type_fibroblasta

------WLPSFHTISP-------------------------------------------------TLFNASPKDMKLKPELFLKPKFLRGHLPKYEQVCILPVGRIEEPFWQNFKHLQEEVFQKIKTLAQLS-KDVQDVVFYSILAML--GDRGALQDLMD------------MLELDSSGHLDGP--------------------GGAILKKLQQYS----NRAWFNPKDPILYLLEAIMVLNDI-QHD-LL--ACSVEMRILLQQQEL---VRSILEP--NFRYPWSI------PFTLK-PELL--APL----------------------------------------------------------------------------------------------------QSEGLAITYGLLEECGLRME-----------LDNPRSTWDLEAKIPLSA--LYGTLSL--LQQLAEA---------------------

>ref|NP_113603.1|_chromosome_8_organism_Homo_sapiens_gcode_1_chromosome_8_map_8q24.21_gasdermin-C_Homo_sapiens

------LLPSFHTISP-------------------------------------------------TLFNASSNDMKLKPELFLTQQFLSGHLPKYEQVHILPVGRIEEPFWQNFKHLQEEVFQKIKTLAQLS-KDVQDVMFYSILAML--RDRGALQDLMN------------MLELDSSGHLDGP--------------------GGAILKKLQQDS----NHAWFNPKDPILYLLEAIMVLSDF-QHD-LL--ACSMEKRILLQQQEL---VRSILEP--NFRYPWSI------PFTLK-PELL--APL----------------------------------------------------------------------------------------------------QSEGLAITYGLLEECGLRME-----------LDNPRSTWDVEAKMPLSA--LYGTLSL--LQQLAEA---------------------

>ref|XP_001153860.1|_location_chromosome_chromosome_8_completeness_complete_organism_Pan_troglodytes_isolate_Yerkes_chimp_pedigree_*C0471_(Clint)_gcode_1_chromosome_8_sex_male_tissue_type_blood_dev_ss

------LLPSFHTISP-------------------------------------------------TLFNASSNDMKLKPELFLTQQFLSGHLPKYEQVHILPVGRIEERFWQNFKHLQEEVFQKIKTLAQLS-KDVQNVVFYSILAML--RDRGALQDLMN------------MLELDSSGHLDGP--------------------GGAILKKLQQDS----NHAWFNPKDPILYLLEAIMVLSDF-QHD-LL--ACSVEKRILLQQQEL---VRSILEPNFRYPWSI--------PFTLK-PELLAPL------------------------------------------------------------------------------------------------------QSEGLAITYGLLEECGLRME-----------LDNPRSTWDVEAKMPLSA--LYGTLSL--LQQLAEA---------------------

>ref|XP_036088919.1|_chromosome_Unknown_completeness_complete_organism_Rousettus_aegyptiacus_isolate_mRouAeg1_gcode_1_chromosome_Unknown_sex_male_tissue_type_muscle_dev_stage_adult_country_USA:_Berkes

FRLQLQSFTGGREIQP----------------------------------------------------------------------------KSGECEIQILSGRAQRSCWLDFGRLQEEVSWYVKKAQLL--EDMGAVVFSSILAML--GDWEALEGLED------------KLEQKPLGHLDGP--------------------GGVILNALRESS----SYQWFNSEDSILYLLEAINGLSDL-QHD-LL--AWSMEKKVLLQQRDL---VRSILEP--NFKYPWNI------PFTMK-PELLA-PLR----------------------------------------------------------------------------------------------------G-EALAITYGLLEECGLTME-----------PNSPRSTWDLEAKKPLSA--LYAALSV--LQQLAEA---------------------

>ref|XP_024427836.1|_chromosome_Unknown_completeness_complete_organism_Desmodus_rotundus_isolate_DRU21DN04_gcode_1_chromosome_Unknown_sex_male_tissue_type_muscle/skin_sample_dev_stage_adult_country_Us

----------------------------------------------------------------------------------------------------------------DFKRLHEEVSKRVIAAAELS-KDIKGVVFSNILAML--GDRGALQDLVH---M--------LEQEPLKGHLNGP------------------GG--TILNELQKDS----SFAWNGSQYLILYLLEAMMVLSDV-QLC-LL--APSMEKKILSQQRDL---VRSILEP-NFK-YPWSI------PFTLK-PELLA-PLQ----------------------------------------------------------------------------------------------------G-EGLAITYGLLDECGLKME-----------LHSPRSTWDLEAKKPLSA--LYGTLSV--LQQLAEA---------------------

>ref|XP_035935879.1|_chromosome_Unknown_completeness_complete_organism_Halichoerus_grypus_isolate_241Hg_ecotype_Northwest_Atlantic_gcode_1_chromosome_Unknown_sex_male_tissue_type_blood_dev_stage_molts

-----------------------------------------------------------------------------------------------------------KEEHGDFKTLKEEVQREIQEVKKLS-RVGQSSLLTSLSKLL--GKKKELQDLELML----------EGALDKGHEVTPE----------------------ALPKDVLVSK---------EAMGGILYFLGALIELSEA-QQK-LL--IKSVEKKILPMQLKL---VESTMEQ--SFLQDKDG------VFPLQ-PDLL--SSL----------------------------------------------------------------------------------------------------GEEELTLTEALVGLSGLEVQ-----------RSGPQYTWDPDTLPRLCA--LYAGLSL--LQLLTKAS--------------------

>ref|XP_004633937.1|_chromosome_Unknown_completeness_complete_organism_Octodon_degus_isolate_3935_gcode_1_chromosome_Unknown_sex_female_gasdermin-A_Octodon_degus

-----------------------------------------------------------------------------------------------------------GEVHEDFKTLKEEVRRETQEVDKLS-RAGQGSLLSSLSNLL--GKKKELQDLELTL----------EGALDKGHEVTLE----------------------ALPKDVLLSK---------ESMGAILYFLGALTVLSEA-QQK-LL--VKSVEKKLLPVQLKL---VESTLEQ--NFLQDKEG------VFPLR-PDLL--SSL----------------------------------------------------------------------------------------------------GEEELTLTEALVGLSGLEVQ-----------RSGPQYTWDPDTLPRLCA--LYASLSL--LQLLARSP--------------------

>sp|Q32M21|GSDA2_MOUSE_Gasdermin-A2_OS_Mus_musculus_OX_10090_GN_Gsdma2_PE_2_SV_1

-----------------------------------------------------------------------------------------------------------GEMHEDFKTLKKEVQQETQEVEKLS-PVGRSSLLTSLSHLL--GKKKELQDLEQML----------EGALDKGHEVTLE----------------------ALPKDVLLLK---------DAMDAILYFLGALTELSEE-QLK-IL--VKSLENKVLPVQLKL---VESILEQ-----NFLQDKED---VFPLR-PDLL--SSL----------------------------------------------------------------------------------------------------GEEDQILTEALVGLSGLEVQ-----------RSGPQYTWNPDTCHNLCA--LYAGLSL--LHLLSRDS--------------------

>sp|Q5Y4Y6|GSDA3_MOUSE_Gasdermin-A3_OS_Mus_musculus_OX_10090_GN_Gsdma3_PE_1_SV_1

-----------------------------------------------------------------------------------------------------------GEMHEDFKTLKEEVQRETQEVEKLS-PVGRSSLLTSLSHLL--GKKKELQDLEQKL----------EGALDKGQKVTLE----------------------ALPKDVLLSK---------DAMDAILYFLGALTELTEE-QLK-IL--VKSLEKKILPVQLKL---VESTLEQ--NFLQDKEG------VFPLQ-PDLL--SSL----------------------------------------------------------------------------------------------------GEEELTLTEALVGLSGLEVQ-----------RSGPQYAWDPDTRHNLCA--LYAGLSL--LHLLSRKSNALTYCALS-----------

>sp|Q9EST1|GSDMA_MOUSE_Gasdermin-A_OS_Mus_musculus_OX_10090_GN_Gsdma_PE_2_SV_1

-----------------------------------------------------------------------------------------------------------GEMHEDFKTLKEEVQRETQEVEKLS-PVGRSSLLTSLSHLL--GKKKELQDLEQTL----------EGALDKGHEVTLE----------------------ALPKDVLLSK---------DAMDAILYFLGALTVLSEA-QQK-LL--VKSLEKKILPVQLKL---VESTMEK--NFLQDKEG------VFPLQ-PDLLSSL------------------------------------------------------------------------------------------------------GEEELILTEALVGLSGLEVQ-----------RSGPQYTWDPDTLPHLCA--LYAGLSL--LQLLSKNS--------------------

>ref|XP_024836266.1|_location_chromosome_chromosome_19_completeness_complete_organism_Bos_taurus_isolate_L1_Dominette_01449_registration_number_42190680_breed_Hereford_gcode_1_chromosome_19_sex_fem...

-----------------------------------------------------------------------------------------------------------GAVHEDFRTLKEEVQRETQEVEKLS-PEGKSSLLSSLSKLL--GKKKELQDLELTL----------EEALGKGHEETLE----------------------ALPKNVLLSK---------GAMDAILYFLGALAELSEA-QQK-LL--VKSMEKKILPVQLKL---VESTMEQ--NFLQDKEG------VFPLR-PELLSSL------------------------------------------------------------------------------------------------------GEEELTLTEALVGLSGLEVQ-----------RSGPQYTWDPDTLPRLCA--LYAGLSL--LRLLTKAS--------------------

>ref|XP_014975037.1|_location_chromosome_chromosome_16_completeness_complete_organism_Macaca_mulatta_isolate_AG07107_bio_material_Coriell:AG07107_gcode_1_chromosome_16_sex_female_tissue_type_fibroblaa

-----------------------------------------------------------------------------------------------------------GDVHEGFGTLKEEVQRETQQVERLS-QAGQSSLLSSLSKLL--GKKKELQDLELAL----------EGALDKGHEVTLE----------------------ALPKDVLLSK---------EAVGAILYFVGALTELSEV-QQK-LL--VKSMEKKILPVQLKL---VESTMEQ--NFLQDKEG------VFPLH-PELL--SSL----------------------------------------------------------------------------------------------------GEEELTLTEALVGLSGLEVQ-----------RSGPQYMWDPDTLPRLCA--LYAGLSL--LQQLTKAS--------------------

>ref|NP_835465.2|_chromosome_17_organism_Homo_sapiens_gcode_1_chromosome_17_map_17q21.1_gasdermin-A_Homo_sapiens

-----------------------------------------------------------------------------------------------------------GDVHEGFRTLKEEVQRETQQVEKLS-RVGQSSLLSSLSKLL--GKKKELQDLELAL----------EGALDKGHEVTLE----------------------ALPKDVLLSK---------EAVGAILYFVGALTELSEA-QQK-LL--VKSMEKKILPVQLKL---VESTMEQ--NFLLDKEG------VFPLQ-PELL--SSL----------------------------------------------------------------------------------------------------GDEELTLTEALVGLSGLEVQ-----------RSGPQYMWDPDTLPRLCA--LYAGLSL--LQQLTKAS--------------------

>ref|XP_001171222.1|_location_chromosome_chromosome_17_completeness_complete_organism_Pan_troglodytes_isolate_Yerkes_chimp_pedigree_*C0471_(Clint)_gcode_1_chromosome_17_sex_male_tissue_type_blood_devs

-----------------------------------------------------------------------------------------------------------GDVHEGFRTLKEEVQRETQQVEKLS-RVGQSSLLSSLSKLL--GKKKELQDLELAL----------EGALDKGHEVTLE----------------------ALPKDVLLSK---------EAMGAILYFVGALTELSEA-QQK-LL--VKSMEKKILPVQLKL---VESTMEQ--NFLLDKEG------VFPLQ-PELLSSL------------------------------------------------------------------------------------------------------GDEELTLTEALVGLSGLEVQ-----------RSGPQYMWDPDTLPRLCA--LYAGLSL--LQQLTKAS--------------------

>ref|XP_024429158.1|_chromosome_Unknown_completeness_complete_organism_Desmodus_rotundus_isolate_DRU21DN04_gcode_1_chromosome_Unknown_sex_male_tissue_type_muscle/skin_sample_dev_stage_adult_country_Us

-----------------------------------------------------------------------------------------------------------GEVHQDFRMLKEEIQRETQEVEKLS-RMGQSSLLTSLSKLL--GKKKELQDLELTL----------EGALDKGHEVALE----------------------ALPKDVLLSK---------EVVGAILYFLGALTELSEA-QQK-LL--VKSMEKKILPVQLKL---VESIMEQ--NFLLDKEG------VFPLQ-PNLLSCL------------------------------------------------------------------------------------------------------GEEELTFTEALVGLSGLEVQ-----------RSGPQYMWDPDTLPDLCA--LYAGLSF--LQLLTKAS--------------------

>ref|XP_016020091.2|_chromosome_Unknown_completeness_complete_organism_Rousettus_aegyptiacus_isolate_mRouAeg1_gcode_1_chromosome_Unknown_sex_male_tissue_type_muscle_dev_stage_adult_country_USA:_Berkes

-----------------------------------------------------------------------------------------------------------GEVHEDFRTLKEEIQRETQEVEKLS-HLGQSSLLTSLSKLL--GNRKELQDLELTL----------EGALGKGHEVTLE----------------------ALPKDVLLSK---------EALGAILYFLGALTELSEV-QQK-LL--VKSMEKKILPVQLKL---VESTMEQ--NFLQDKEG------VFPLQ-PDLLSSL------------------------------------------------------------------------------------------------------GEEDLTLTEALVGLSGLEVQ-----------RSGPQYTWDPDTLPHLCA--LYAGLSF--LQLLTKAS--------------------

>ref|XP_006924750.1|_chromosome_Unknown_completeness_complete_organism_Pteropus_alecto_gcode_1_chromosome_Unknown_sex_male_tissue_type_kidney_country_Australia:_Anglican_Church_Grammar_School_East_Bro

-----------------------------------------------------------------------------------------------------------GEVHEDFRTLKEEIQRETQEVEKLS-HVGRSSLLTSLSKLL--GNRKELQDLELTL----------EGALGKGHEVTLE----------------------ALPKDVLLSK---------EALGAILYFLGALTELSEV-QQK-LL--VKSMEKKILPVQLKL---VESTMEQ--NFLQDKES------VFPLQ-PDLLSSL------------------------------------------------------------------------------------------------------GEEDLTLTEALVGLSGLEVQ-----------RSGPQYTWDPDTLPCLCA--LYAGLSL--LQLLTKAS--------------------

>ref|XP_023358160.1|_location_chromosome_chromosome_4_completeness_complete_organism_Sarcophilus_harrisii_gcode_1_chromosome_4_cell_line_91H_gasdermin-B_Sarcophilus_harrisii

--------------------------------------------------------------------------------------------------------------------------AVLDLLTRLLQDEALGEMEGKVAMAL-------------------------FTEELSDPENPLL----------------------SVLFDASGSS-------MQNQAEGILDFLEALTELKEE-QQK-IL--PKVVEKGLLSETMKL---VEDLLRQ----------------SETF---------------------------------------------------------------------------------------------------------------SLKADSITHTLVGICDPEGK------------LEQPVTWDSETCHSLCA--LYTVLSV--LMLLAQGSVSVP----------------

>ref|NP_001231146.1|_chromosome_19_organism_Bos_taurus_gcode_1_chromosome_19_map_19_gasdermin-B_Bos_taurus

--------EALNCFTK-------------------------------------------------------------------------------------------------CLSSEEELQDLEQRVSEVQ-CSGELQMNSPANSLI-------------------------SSLFNAAGALIEA------------------------------------------RAETIWDVLDALMELSEY-RQ--FV--AEVLDKGELRPVKDK---VESILEE----------------NRCEG-PLDVSCDPE----------------------------------------------------------------------------------------------------ARTLCALYVVVSILLQLSEK--------------------------------PTSVSS------------------------------

>ref|XP_035935913.1|_chromosome_Unknown_completeness_complete_organism_Halichoerus_grypus_isolate_241Hg_ecotype_Northwest_Atlantic_gcode_1_chromosome_Unknown_sex_male_tissue_type_blood_dev_stage_molts

---------------------------------------------------------------------------------------------------------------DVLSCLRGQLPLSAKVFEVLI-SGSRHLEMEGLVGLL-------------------------ISSLFNAAGFLVK--------------------------------------------AHTESILDVLDALITS-EEQHLV--AEALEKGTLPLLKDQ---VRSVLEQ----------------NWSEQ-PHDVGWDPD----------------------------------------------------------------------------------------------------AXLLCALYVALSLLLQLGEK--------------------------------PTSVPS------------------------------

>ref|XP_014975035.1|_location_chromosome_chromosome_16_completeness_complete_organism_Macaca_mulatta_isolate_AG07107_bio_material_Coriell:AG07107_gcode_1_chromosome_16_sex_female_tissue_type_fibroblaa

-----------------------------------------------------------------------------------------------------------------------------DVLNFLTKCLGREELWQDLEQKI--SEVLIFGE---------------LQMEDPVNPLLSS-----------------------------LFN--AAGILVGARAEAILDFLDALLELSEE-QH--LV--AEALEKGTLPLLKDQ---VKPIMEQ----------------NWDEL-ASSPHDMDC----------------------------------------------------------------------------------------------------DPEARIPCALYVVVSVLLEL--------------------AEGTTSVSS---------------------------------------

>ref|NP_001159430.1|_chromosome_17_organism_Homo_sapiens_gcode_1_chromosome_17_map_17q21.1_gasdermin-B_isoform_3_Homo_sapiens

-------------------------------------------------------------------------------------------------------------------------DVLNSLAKCLGKEDIRQDLEQRVSEVLISGE---------------------LHMEDPDKPLLSS-----------------------LFNAAGVLV--------EARAKAILDFLDALLELSEEQQFV-----AEALEKGTLPLLKDQ---VKSVMEQ----------------NWDEL-ASSPPDMDY----------------------------------------------------------------------------------------------------DPEARILCALYVVVSILLEL--------------------AEGPTSVSS---------------------------------------

>ref|XP_009430521.1|_location_chromosome_chromosome_17_completeness_complete_organism_Pan_troglodytes_isolate_Yerkes_chimp_pedigree_*C0471_(Clint)_gcode_1_chromosome_17_sex_male_tissue_type_blood_devs

-------------------------------------------------------------------------------------------------------------------------DVLNSLAKCLGKEEIRQDLEQRVSEVLISRE---------------------LHMEDPDKPLLSS-----------------------LFNAAGVLV--------EVRAKAILDFLDALLELSEE-QQ--FV--AEALEKGTLPLLKDQ---VKSVMEQ----------------NWDEL-ASSPPDMDC----------------------------------------------------------------------------------------------------DPEARILCALYVVVSVLLEL--------------------AEGPTSVSS---------------------------------------

>ref|XP_024429114.1|_chromosome_Unknown_completeness_complete_organism_Desmodus_rotundus_isolate_DRU21DN04_gcode_1_chromosome_Unknown_sex_male_tissue_type_muscle/skin_sample_dev_stage_adult_country_Us

-------------------------------------------------------------------------------------------------------------------------NVLSCLTKCLT-SDGELQALEERVSEVLVSEE--------------------LQVEGPADPLISS-----------------------------LFN--AAGILVEACIEAILDFLDALKELSEE-KE--LV--AETLEKGTLPLLKDQSGGLKSVLEQ----------------EWGEQ-PRGVGRDPE----------------------------------------------------------------------------------------------------AQTVCTLYLVVSILLQLSEK-------------------------PAPA-----------SSC-------------------------

>ref|XP_024897515.1|_chromosome_Unknown_completeness_complete_organism_Pteropus_alecto_gcode_1_chromosome_Unknown_sex_male_tissue_type_kidney_country_Australia:_Anglican_Church_Grammar_School_East_Bro

-------------------------------------------------------------------------------------------------------------------------DALSCLTKCLTKDEQLQDLEQRMSEVLISGE---------------------LQMDSPAGPLISS-----------------------LFNAAGILV--------ETRTEAIMDFLDAVMELFEE-KE--LV--GEALEKGTLPLLKEQ---VESVLDQ----------------NGGEQ-PECVGCDPE----------------------------------------------------------------------------------------------------AQILHALYVAVSILLQLSEK--------------------------------PASASS------------------------------

>ref|XP_036078993.1|_chromosome_Unknown_completeness_complete_organism_Rousettus_aegyptiacus_isolate_mRouAeg1_gcode_1_chromosome_Unknown_sex_male_tissue_type_muscle_dev_stage_adult_country_USA:_Berkes

-------------------------------------------------------------------------------------------------------------------------DALSCLSKCLTKDEQLQDLEQRVSEIL----------------------------ISGELQMDSP--------------------AGLLINSLFNAS----GILVETRTEAIMDFLDALMELSEE-KE--LV--AEALEKGTLPLLKEQ--------------------------NCGEQ-PEYVGWDPE----------------------------------------------------------------------------------------------------AQVLRTIYAAVSILLLLSEK--------------------------------PASVSC------------------------------

>WCB86927.1_CPPV116_hypothetical_protein_[Cooks_petrelpox_virus]

--------------------------------------------------------------------------------------------------------------------------MYINIFDVLS-RDGKISMLDDETISI-------------------------VVDKDNNYSLIKS-----------------------LYDRRRLDK-----ELTERYENNIDTLYKYFFDISEN-SKYYSI---PEIILKAYKTESDINMKLKEIKNL----------------EPNEQ-HTYVYNLVN----------------------------------------------------------------------------------------------------EFRNTGSYYMYRLIDKINTI-----------IKSNNTILTDKIDIMFKEAILFRR-NI------------------------------

>ARF02807.1_SWPV1-246_[Shearwaterpox_virus]

--------MGNITGNV-------------------------------------------------------------------------------------NDNISEEYKEKLFSAINNSNERYVKCFDCLC-DDGKILFLDGMVIGA-------------------------AKHNHYEPHLFIR-----------------------TLKDTGRLN--------SELVKRCDNNFSLLREYFDK---------NVISTPDIIIKDTEM---HKNIEDK----------------IKELKLLDYIGKYVR----------------------------------------------------------------------------------------------------DFIEFGNYNSYALIYRLRLE---------------------DNIRFDVDKMILERLKV--IK--------------------------

>UOX38602.1_hypothetical_protein_[Finch_poxvirus]

--------MGNNIQLQ----------------------------------------------------------------------------------------PITDGIIRLSGAINDQNTLLVNIFDVLD-YDGKKLMIDSISKSI-------------------------LENEDFEPYLFIK--------------------SLQDLGKLDSEL--------------SERLDKNISIINEY-HRK-----SGSTRPEVLVNIDKI---QDSVNAK--LAVLNEIS------DILEK-ENYINKIIY----------------------------------------------------------------------------------------------------KFVYTGSFENYMLLRYIYYN-----------YSQVECKLWHKVQNILEDTVAKRKLYISSHEA-------------------------

>NP_955035.1_hypothetical_protein_CNPV012_[Canarypox_virus]

--------MGNNIQSQ----------------------------------------------------------------------------------------FMPNNIIRLSGAISDQNTLLVNVFDALN-YEGKKLMIDSMSEPI-------------------------SESKDFEPYLFIK--------------------SLHDFGKLDSEL--------------SERLEKNISVINEY-RCK-----SESIRPEILVNLDKI---QDSVNTR--------LAVLNGINDIVEK-ENYINKIID----------------------------------------------------------------------------------------------------EFVYTGGFQNYMLLRSIYYN-----------YSQIEGNLGYKAQNILDDAISKRKLYI-SSHKA------------------------

>WCB86827.1_CPPV016_hypothetical_protein_[Cooks_petrelpox_virus]

--------MGDIIIVG----------------------------------------------------------------------------------DDSIADIPKEYIDRLTQAIKNSNNRQVEVFDCLC-REGKKYMINDVTIAI-------------------------SKYNDYEPHLFLK--------------------TLYTSGKLDAEL----VEMYDKNSRTCHEYFSALKNLRDT-D-------DGLTKPEIYIQILDM---YKAAESK-----IKDKLVL----GYNDK-KSYIDNVID----------------------------------------------------------------------------------------------------KFARTGSYWSYVIIEKIRQY-----------FSKCGGELGRKAQNVVEDAILARKAYI-TLNA-------------------------

>YP_009448176.1_hypothetical_protein_C1178_gp283_[Flamingopox_virus_FGPVKD09]

--------MGDIIIVG----------------------------------------------------------------------------------DDSIADIPKEYIDRLTQAIKNSNNRQVEVFDCLC-REGKKSMINDVTIAI-------------------------SKYNDYEPHLFLK--------------------TLYTSGKLDAEL----VEMYDKNSRTCHEYFSALKNLRDT-D-------DGLTKPEIYIQILDM---YKAAESK-----IKD-KLVL---GYNDK-KSYIDNVIY----------------------------------------------------------------------------------------------------KFARTGSYWSYVIIEKIRQY-----------FSKCGGELGRKAQNVVEDAILARKAYI-TLNT-------------------------

>YP_009046354.1_hypothetical_protein_HM89_gp126_[Pigeonpox_virus]

---MEYYNPSDASLIG----------------------------------------------------------------------------------------VCKEYVDLLTHVVVESNKKPIDVFDCLS-REGKKLMIASIIISI-------------------------SRCHEFEPRLFLQ--------------------VLHNSGKLDDEL-----LEIINMEKECTEYFSKIKELENH-NAYDAI--AKLQKPELYIQISSI---DEVIENK-----FKDKLLL----NYDEK-KNYIDEMIQ----------------------------------------------------------------------------------------------------QFACTGSYHAYSTLGHININ-----------FSEYEGELSRKARSVLDDALIARKLYL--VNH-------------------------

>WCB86986.1_CPPV175_hypothetical_protein_[Cooks_petrelpox_virus]

--------MEYSNPIN--------------------------------------------------------------------------------------APFIDEYVDLLTSVVVEGNQRPLDVFNCLS-REGKKLMIGAITISI-------------------------SRFHEFEPRLFLQ--------------------LLHKYGKLDGEL-----LEIINKENECREYFSKIKELENR-NAYDEI--TKLPKPELYTQISSI---DEVMEDA-----FKDKLLL----NYDDK-KEYIDEMIQ----------------------------------------------------------------------------------------------------QFACTGSYHTYITLIYINHN-----------FSKYDGELSKKARSVLDDALIARKLYL--VNS-------------------------

>YP_009448040.1_hypothetical_protein_C1178_gp125_[Flamingopox_virus_FGPVKD09]

--------------------------------------------------------------------------------------------------------------------MLEGNQRPLEVFNCLS-REGKKLMIGAITISI-------------------------SRFHEFEPQLLHK------------------------SGKLDSEL-----LEIINMENECREYFSKIKELENC-NAHDEI--TKLPKPELYTQISSI---DEVIEDK-----FKDKLLL----NYDDK-KKYIDEMIQ----------------------------------------------------------------------------------------------------KFACTGSYHTYVTLIYTNNN-----------FSEYNGELSKKARSVLDDALIARKSYL--VNS-------------------------

>AXY05184.1_hypothetical_protein_[Fowlpox_virus]

--------MGNNNTXG----------------------------------------------------------------------------------SKKNRLSMIYSKKRITSFIRKHHNYLMRVFDYLS-DNGKVVMIDCMGHWL-------------------------YNKWDYEPNMFLK----------------------CISERSDIES------IYRELAKKTLEDAKNYHNIFPR-TVR-----DNLSISNKLKTVMDN---NDILIKKYNSA------------NYYIK-KEIIDTIVN----------------------------------------------------------------------------------------------------EFKIKRTYEPYLFLQTLERQ----------LRGSNDYFYND----------ILQAITI------------------------------

>AXY04662.1_hypothetical_protein_[Fowlpox_virus]

--------MGNNNTRG----------------------------------------------------------------------------------SKKNRLSMIYSKKRITSFIRKHHNYLMRVFDYLS-DNGKVVMIDCMGHWL-------------------------YNKWDYEPNMFLK----------------------CISERSDIES------IYRELAKKTLEDAKNYHNIFPR-TVR-----DNLSISNKLKTVMDN---NDILIKKYNSA------------NYYIK-KEIIDTIVN----------------------------------------------------------------------------------------------------EFKIKRTYEPYLFLQTLERQ----------LRGSNDYFYND----------ILQAITI------------------------------

>NP_039184.1_hypothetical_protein_FPV221_[Fowlpox_virus]

--------MGNNNTHG----------------------------------------------------------------------------------SKKNRLSMIYSKKRITSFIRKHHNYLMRVFDYLS-DNGKVVMIDCMGHWL-------------------------YNKWDYEPNMFLK----------------------CISERSDIES------IYRELAKKTLEDAKNYHNIFPR-TVR-----DNFSISNKLKTVMDN---NDILIKKYNSV------------NYYIK-KEIIDTIVN----------------------------------------------------------------------------------------------------EFKIRRTYEPYLFLQTLERQ----------LRGSNDYFYND----------ILQAITI------------------------------

>ART91654.1_vaccinia_A47L-like_protein_[Fowlpox_virus]

--------MGNNNTHG----------------------------------------------------------------------------------SKKNRLSMIYSKKRITSFIRKHHNYLMRVFDYLS-DNGKVVMIDCMGHWL-------------------------YNKWDYEPNMFLK----------------------CISERSDIES------IYRELAKKTLEDAKNYHNIFPR-TVR-----DNLSISNKLKTVMDN---NDILIKKYNSA------------NYYIK-KEIIDTIVN----------------------------------------------------------------------------------------------------EFKIRRTYEPYLFLQTLERQ----------LRGSNDYFYND----------ILQAITI------------------------------

>AYO89814.1_hypothetical_protein_FPV221_[Fowlpox_virus]

--------MGNNNTHG----------------------------------------------------------------------------------SKKNRLSMIYSKKRITSFIRKHHNYLMRVFDYLS-DNGKVVMIDCMGHWL-------------------------YNKWDYEPNMFLK----------------------CISERSDIES------IYRELAKKTLEDAKNYHNIFPR-TVR-----DNFSISNKLKTVMDN---NDILIKKYNSA------------NYYIK-KEIIDTIVN----------------------------------------------------------------------------------------------------EFKIRRTYEPYLFLQTLERQ----------LRGSNDYFYND----------ILQAITI------------------------------

>YP_009046447.1_A47L-like_protein_[Pigeonpox_virus]

--------MGNNNTYG----------------------------------------------------------------------------------SKKNRLRIISSKKRITSFIRKHHNYLMRVFDYLS-DDGKVVMIDCMGHWL-------------------------YNKWDYEPNMFLK----------------------CISERSDIES------IYRELAKKTLEDAKNYHDIFSR-AVR-----DNLGISNKLRIVMDN---NDILIKKYNST------------NHYMK-KEIIDNKVN----------------------------------------------------------------------------------------------------EFQTKRTYEPYLFLQTLERQ----------LRGSNDYFYND----------ILQAITL------------------------------

>YP_009448146.1_hypothetical_protein_C1178_gp240_[Flamingopox_virus_FGPVKD09]

--------MGNNNTRG----------------------------------------------------------------------------------SKKNRLSMISSKKRITSFIRKHHNYLMKVFDYLS-DDGKVVMIDCMGHWL-------------------------YNKWDYEPNMFLK----------------------CISERSDIES------IYRELAKKTLEDAKNYHDIFSR-AVR-----DNLGISNKLRTVMDN---NDILIKKYNST------------NYYMK-KEIIDSKVN----------------------------------------------------------------------------------------------------EFQTKRTYEPYLFLQTLERQ----------LRGSNDYFYND----------ILQAITL------------------------------

>YP_009046216.1_A47L-like_protein_[Penguinpox_virus]

--------MGNNNTRG----------------------------------------------------------------------------------SKKNRLSMISSKKRITSFIRKHHNYLMKVFDYLS-DDGKVVMIDCMGHWL-------------------------YNKWDYEPNMFLK----------------------CISERSDIEA------IYRELAKKTLEDAKNYHDIFSR-SVP-----NNLGISNKLRTVMDN---NDILIKKYNST------------NYYMK-KEIIDSKVN----------------------------------------------------------------------------------------------------EFQTKRTYEPYLFLQTLERQ----------LRGSNDYFYND----------ILQAITL------------------------------

>URH27575.1_hypothetical_protein_99866_00235_[Fowlpox_virus]

--------MGNASSMI----------------------------------------------------------------------------------HTVNNNP-------YHKLNSSSDNTLLDVFSVMS-DDGKIDMIEAAAVHL-------------------------LKSWNYELQLFLS-----------------------SLQDIDMTK------------RVKDRLVDTTKIVYEN-RNKEID--EYPVILDGFKNIQDK---ISIVEKE-----ITDFC------NNEIQ-EDKLEYKAN----------------------------------------------------------------------------------------------------QFKKKGDYRSYLFLKMLAEQ-------------DSDISLREKTKSLLKEIISYRKKIY--DKIYDKLHTIL-----------------

>AXY04933.1_hypothetical_protein_[Fowlpox_virus]

--------MGNASSMI----------------------------------------------------------------------------------HTVNNNP-------YHKLNSSSDNTLLDVFSVMS-DDGKIDMIDAAAVHL-------------------------LKSWNYELQLFLS-----------------------SLQDIDMTK------------RVKDRLVDTTKIVYEN-RNKEID--EYPVILDGFKNIQDK---ISIVEKE-----ITDFC------NNEIQ-EDKLEYKAN----------------------------------------------------------------------------------------------------QFKKKGDYRSYLFLKMLAEQ-------------DXDISLREKTKSLLKEIISYRKENI------------------------------

>ART91662.1_hypothetical_protein_[Fowlpox_virus]

--------MGNASSMI----------------------------------------------------------------------------------HTVNNNP-------YHKLNSSSDNTLLDVFSVMS-DDGKIDMIDAAAVHL-------------------------LKSWNYELQLFLS-----------------------SLQDIDMTK------------RVKDRLVDTTKIVYEN-RNKEID--EYPVILDGFKNIQDK---ISIVEKE-----ITDFC------NNEIQ-EDKLEYKAN----------------------------------------------------------------------------------------------------QFKKKGDYRSYLFLKMLAEQ-------------DSDISLREKTKSLLKEIISYRKKNI------------------------------

>AYO89822.1_hypothetical_protein_FPV229_[Fowlpox_virus]

--------MGNASSMI----------------------------------------------------------------------------------HTVNNNP-------YHKLNSSSDNTLLDVFSVMS-DDGKIDMIDAAAVHL-------------------------LKSWNYELQLFLS-----------------------SLQDIDMTK------------RVKDRLVDTTKIVYEN-INKEID--EYPVILDGFKNIQDK---ISIVEKE-----ITDFC------NNEIQ-EDKLEYKAN----------------------------------------------------------------------------------------------------QFKKKGDYRSYLFLKMLAEQ-------------DSDISLREKTKSLLKEIISYRKENI------------------------------

>NP_039192.1_hypothetical_protein_FPV229_[Fowlpox_virus]

--------MGNASSMI----------------------------------------------------------------------------------HTVNNNP-------YHKLNSSSDNTLLDVFSVMS-DDGKIDMIDAAAVHL-------------------------LKSWNYELQLFLS-----------------------SLQDIDMTK------------RVKDRLVDTTKIVYEN-RNKEID--EYPVILDGFKNIQDK---ISIVEKE-----ITDFC------NNEIQ-EDKLEYKAN----------------------------------------------------------------------------------------------------QFKKKGDYRSYLFLKMLAEQ-------------DSDISLREKTKSLLKEIISYRKENI------------------------------

>URH28352.1_hypothetical_protein_V_cmp_00231_[Fowlpox_virus]

--------MGNASSMI----------------------------------------------------------------------------------HTVNNNP-------YHKLNSSSDNTLLDVFSVMS-DDGKIDMIDAAAVHL-------------------------LKSWNYELQLFLS-----------------------SLQDIDMTK------------RVKDRLVDTTKIVYEN-RNKEID--EYPVILDGFKNIKDK---ISIVEKE-----ITDFC------NNEIQ-EDKLEYKAN----------------------------------------------------------------------------------------------------QFKKKR----------------------------------------------------------------------------------

>UNS14472.1_ALPV-308_[Albatrosspox_virus]

--------MGNASSMI----------------------------------------------------------------------------------HTVNNNP-------YHKLNSSSDNTLLDVFSVMS-DDGKIDMIDAAAVHL-------------------------LKSWNYELQLFLS-----------------------SLQDIDMTK------------RVKDRLVDTTKIVYEN-RNKEID--EYPVILDGFKNIQDK---ISIVEKE-----ITDFC------NNEIQ-EDKLEYKAN----------------------------------------------------------------------------------------------------QFKKKR----------------------------------------------------------------------------------

>YP_009046221.1_putative_A47L-like_protein_[Penguinpox_virus]

--------MGNASSMI----------------------------------------------------------------------------------YTVNNNPLQDYK------FYDNDNMLLDAFSVMS-DDGKIDMIDATANHL-------------------------LKSWNYELQLFLS-----------------------SLQDIDMDR------------RVKDRLIDNTKIVYEN-RNKEIS--EYSVIVDGFKNIRDK---ISAVEKE-----IADFY------NNESQ-RDKLEYKAN----------------------------------------------------------------------------------------------------QFKKKGDYESYLFLKMLLDQ-------------EHDVSLSDKTNSLLKETISYRKENT----C-------------------------

>YP_009046452.1_putative_A47L-like_protein_[Pigeonpox_virus]

--------MGNASSMI----------------------------------------------------------------------------------YTVNNNPLQDY---KFYYNSSYDNTLLDAFSVMS-DDGKIDMIDATANHL-------------------------LKSWNYELQLFLS-----------------------SLQNTDMDK------------RVKDRLIDNTKIVYEN-RNKEIS--ESSVIVDGFKNIRDK---ISVVEKE-----IADFY------NNESQ-RDKLEYKAN----------------------------------------------------------------------------------------------------QFKKKGDYESYLFLKMLSEQ-------------EHDVSLRDKTKSLLKETISYRKENT----C-------------------------

>YP_009448152.1_putative_A47L-like_protein_[Flamingopox_virus_FGPVKD09]

--------MGNASSMI----------------------------------------------------------------------------------YTVNNNPLQDH---KFYYNSSYDNTLLDAFSVMS-DDGKIDMIDATANHL-------------------------LKSWNYELQLFLS-----------------------SLQNIDMDK------------RVKDRLIDNTKIVYEN-RNKEIS--ESSVIVDGFKNIRDK---ISAVEKE-----IADFY------NNESQ-RDKLEYKVN----------------------------------------------------------------------------------------------------QFKKKGDYESYLFLKMLLEQ-------------EHDVSLRDKTKSLLKETISYRKENT----C-------------------------

>WCB87127.1_CPPV316_putative_A47L-like_protein_[Cooks_petrelpox_virus]

--------MGNASSMI----------------------------------------------------------------------------------YTVNNNPLQDH---KFYYNSSYDNTLLDAFSVMS-DDGKIDMIDATANHL-------------------------LKSWNYELQLFLS-----------------------SLQNIDMDK------------RVKDRLIDNTKIVYEN-RNKEIS--ESSVIVDGFKNIRDK---ISAVEKE-----IADFY------NNESQ-RDKLEYKAN----------------------------------------------------------------------------------------------------QFKKKGDYESYLFLKMLLEQ-------------EHDVSLRDKTKSLLKETISYRKENT----C-------------------------

>ARF02840.1_SWPV1-281_[Shearwaterpox_virus]

--------MGNASAMI-----------------------------------------------------------------------------------------YQDNNDSTYKSYYKTNEKLLDLFSILP-DKDKNIMIDYITNSL-------------------------YNVWNKDYYLFLV----------------------SLQQNIYISD------------SIKNNLITNLDIIKEN-KNT--------NTCEKFKYIHNM---ILSINKE-----VSDFCLL----DYNKR-DTIINLKVE----------------------------------------------------------------------------------------------------SFTKNGDFETYLLLDKLKYK----------------DNMFQKISTSIDQAIKRRNYYA--I---------------------------

>AYP74072.1_hypothetical_protein_[Fowlpox_virus]

--------MGNASSMV----------------------------------------------------------------------------------YQENNNSLYRVHYDRYKY--EENKKLLDIFSILS-DKDKNIMIDYIASSL-------------------------CNSWNSEFNLFLI-----------------------SLQDTNIND------------IIKDNLRINLGIVNEG---------KNTSTLEKFKYIENI---ISSVDKE-----ILEFYSL----DYDEK-DNIINEKVE----------------------------------------------------------------------------------------------------SFVENGDFKTYLFLNTLLKN------------ADNMFQRVSNSLEKAISSRKYRLTNN------------------------------

>QRI43019.1_hypothetical_protein_ChPV301_[Cheloniid_poxvirus_1]

--------MGNASSMM----------------------------------------------------------------------------------YKGNNNSYTAHCD-NYKCEENENKKLLDIFYLLS-DKDKNIMIDYIASSL-------------------------CDSWSSEFDLFLT-----------------------SLQDKNINN------------IVKDNLRINLGIVNEG-KNS----------SKKLKYIEKM---ISSIDEE-----ISEFYSV----DYNEK-NNMINDKID----------------------------------------------------------------------------------------------------IFSKNGDFKAYLFLNKLLKS-SLNSTNQVKTIVDNSDDMFRRISASIEEAVNYRKSAN-N----------------------------

>QGM48949.1_conserved_hypothetical_protein_[Magpiepox_virus]

--------MGNASSMI----------------------------------------------------------------------------------YKGNNNP---SCSNNYKLVDGEKVKLMDLFTVLS-DTDKNVMLDYITNSL-------------------------FSCWNQEFHLFL-----------------------MLLRDTEVDG------------ISKDNLITNLDIINED-KQS----------TEKLKYIEKM---ISSIDEE-----ISEFYSV----DYNEK-NNTINKKID----------------------------------------------------------------------------------------------------IFSKNGDFKAYLFLNKLLKS-SLNSTNQVKTIVDNSDDMFRRIPASIEEAVNYRKSAN-N----------------------------

>UOX38782.1_hypothetical_protein_[Finch_poxvirus]

--------MGNASSMI----------------------------------------------------------------------------------YKGNNNSCITCYS-NYKLVDGEKIKLMDLFTVLS-NTDKNVMLDYITNSL-------------------------FSCWNQEFHLFLM-----------------------LLRDTEVDG------------IIKDNLVTNLNIINED-KQS----------TEKLKYIEKM---ILSIDEE-----ISEFYSI----DCNEK-NNMINDKID----------------------------------------------------------------------------------------------------IFSKNGDFKAYLFLNRLLKS-------------SLNSTTNQAKTIFCNSDDMFRRISASIEEAVNYRKSTNN----------------

>NP_955325.1_hypothetical_protein_CNPV302_[Canarypox_virus]

--------MGNASSMM----------------------------------------------------------------------------------YKGNNNSCI-TCSSNYKLVDGEKVKLMDLFTVLS-NTDKNVMLDYITNSL-------------------------FSRWNQEFHLFLM-----------------------LLMDTEVDG------------IIKDNLVTNLNIINED-KQS----------TEKLKYIEKM---ISSIDEE-----ISEFYSV----DYNEK-NNMINDKID----------------------------------------------------------------------------------------------------IFSKNGDFKAYLFLNKLLKSSLNSTNQVKTIVDNSDDMFRRISASIEEAVNYRKSANN------------------------------

>ARF02766.1_SWPV1-192_[Shearwaterpox_virus]

--------------------------------------------------------------------------------------------------------------------MSRSEKLLKKAFEVLN-RDGKKLMLDDLCISI-------------------------AKNLDVKSCKFIE----------------------LLQRKDKLDD------------ELILRVYENINTINNY-IKF-----SHSNCPDIINSINNIDSDNERLLNE-----INKVE------DVGSK-IRMIESVMK----------------------------------------------------------------------------------------------------EFISKGSYESYLLLRIIQSN----------YYNTNDKRLNKITSDIVRRSINTRYKLL------------------------------

>QRY18970.1_ORF-100_[Teiidae_poxvirus_1]

--------MGDFQSAV---------------------------------------------------------------------------------------------------------------FVALN-REGKKHLLDDLCVTF-------------------------VENADYSSLLLIY---------------------SLVKMDMELID-----QVYQTLYTVSSYIYSPDKHISDE-YRPEALKKIDQIENEVNERFLEI-------DRS----------------ENHIK-QEKIDEITS----------------------------------------------------------------------------------------------------EFILNGSYISYLLLKDIRLN------------YCSDNWLKDKIEHLVEYSVEARNLYV--ENVPNLHRR-------------------

>YP_009177139.1_hypothetical_protein_ASN15_gp118_[Turkeypox_virus]

----------------------------------------------------------------------------------------------------------------MYKMKDCESNTACSIFDILD-DEGKNYMIEDICISI-------------------------AEDDDASDKTLLE---------------------------FLRST----RRITENTYKKIMSSIHTIRCYIST-PHSEIQ--DVPYMLKHYNEVESI---VNNRFLN-----IDKID------SFIER-EKVIDSIVE----------------------------------------------------------------------------------------------------EFVNTGNYLSYLTLKDIKFN-----------YGMDRGRLGNKISFILEDALLARYRRV--NSV-------------------------

PYD Alignment

>sp|Q8WXC3|PYDC1 HUMAN Pyrin domain-containing protein 1 OS=Homo sapiens OX=9606 GN=PYDC1 PE=1 SV=1

--------------------MGTKREAIL-KVLENLTPEELKKFKMKLGTVPLREGFE----RIPRGALGQLDIV-DLTDKLVAS--YYEDYAAELVVAVLRDMR-----------MLEEAAR-LQRAA-----------

>NP 660183.1:1-88 apoptosis-associated speck-like protein containing a CARD isoform b Homo sapiens

--------------------MGRARDAIL-DALENLTAEELKKFKLKLLSVPLREGYG----RIPRGALLSMDAL-DLTDKLVSF--YLETYGAE-LTANVLRDM-----------GLQEMAG-QLQAA-----------

>NP 001104231.1 uncharacterized protein LOC796649 Danio rerio

--------------------MASTKTEIL-DVLDELRERE---FKD---FKWRLSNKETLENSIPRGKLENADRQ-DVVDYMEQH--FGTSEAGK-VAVRL--LH-----------SMNQNNL-AEQLKKTLAVVSKV--

>q919n6 danio asc

-------------------MAESFKEHLQ-EAFEDLGADN---LRK---FKSKLGDRRQEP-RVTKSAIEKLKDEIDLADLMVGV--FTSKDAVS-VTVEI--LR-----------AIKCNAV-ADDLLRNTGQS-----

>q56p42 pydc2 human

-----------------MASSAELDFNLQ-ALLEQLSQDELSKFKS---LIRTISLGK----ELQTVPQTEVDKA-N-GKQLVEI--FTSHSCS--YWAGMAAIQ-----------VFEKMNQ-THLSGRADEHCVM---

>q91vj1 pycard mouse

-------------------MESEYREMLLLTGLDHITEEE---LKR---FKYFALTEF----QIARSTLDVADRT-ELADHLIQS--AGAASAVTKAINIFQKLN-----------YMHIANA-LEEKKKE---------

>o35368 ifi203 mouse

--------------------MAEYKNIVLLKGLENMEDYQ---FRT---VKSLLRKEL----KLTKKMQEDYDRI-QLADWMEDK--FPK---DA-GLDKL--IK-----------VCEHIKD-LKDLAKKLKTEKAK--

>q504j1 caspb danio

---------------------------LS-DVLEDLVESELKQFTR---QLWIGVKPGVE--PIPRGKLENKDRQ-DVVDSMVQQ--YSED-AGTITVQTL--RK-----------IKQNERA-----------------

>q919l7 caspa danio

--------------------------HLQ-DALSNIGADN---LRR---FQSRLGDRKQEP-RVRKSTIEKLKDEIDLVDLLVNT--FTSD-AVS-VTVDI--LR-----------GIKCNAV-AE--------------

>tr|B2CWB9|B2CWB9 9POXV M013 OS=Myxoma virus OX=10273 GN=m013L PE=4 SV=1

---------------------MEHRGVII-TVLENLSDYQ---FKM---FIYLAMEDL----YIERAEKEKIDRI-DLAHKISEQ--YLGTDYIE-FMKRV--TD-----------FIPNKVY-VDSLLARAEADA----

>NP 051902.1 Hypothetical protein SFV s013L Rabbit fibroma virus

---------------------MEHRGVII-TVLENLTDYQ---FKM---FLYLVTEDL----RINPVEKEKIDRI-DLAYKISEL--YPGHSYIE-FMKQV--TG-----------YIPNKVY-VDSLLKNAEENT----

>YP 227401.1 hypothetical protein DpV83gp024 Deerpox virus W-848-83

---------------------MELRSAII-AVLENINRYQ---FKM---VIFIVQDEL----YIEEEEKLTMDRI-DLAEKLIKK--YKD---IR-SLYFL--LN-----------IFKEMPD-TEFVKKQLSDYIKRF-

>NP 073403.1 18L protein Yaba-like disease virus

-----------------MLYTMRIKSAII-FSLEDVTHYQ---FKI---LIFLTKDEL----NISDEEKQILDRV-DFAEKLFQT--YPG---IK-SLYFL--EK-----------AISMVPN-AKYARSNINRLISDL-

>ABQ43490.1 pyrin domain Tanapox virus

-----------------MLYNMRIKSAII-FSLEDVTHYQ---FKI---LIFLTKDEL----NINDEEKQILDRV-DFAEKLFQA--YPG---IK-SLYFL--EK-----------AISMVPN-AKYARSNINRLISDL-

>NP 570174.1 Hypothetical protein SWPVgp014 Swinepox virus

---------------------MELRYTII-SVLERLTPYQ---FKT---LLFLIQDDI----NISNDDINVLDRV-DLAIKIMNK--YNN---YR-AIYFL--YK-----------VILRIHN-TEYISGTLQRSIQNI-

>Vaccinia

-----MVKNNKISNSCRMIMSTNPNNILM-RHLKNLTDDE---FKC---IIHRSSDFL----YLSDSDYTSITKE-TLVSEIVEE--YPDD-CNK-ILAII--FL-----------VLDKDID-VDIET-----------

>Buffalopox

-----MVKNNKISNSCLMIMSTNPNNILM-RHLKNLTDDE---FKC---IIHRSSDFL----YLSDSDYTSITKE-TLVSEIVEE--YPDD-CNK-ILAII--FL-----------VLDKDID-VDIET-----------

>Cowpox

MVKSNKIQKNKISNSCRMIMSTNPNNILM-RHLKNLTDDE---FKC---IIHRSSDFL----YLSDRDYTSITKE-TLVSEIVEE--YPDD-CNK-ILAII--FL-----------VLDKDID-VDIET-----------

>Rabbitpox

-----MVKNNKISNSCLMIMSTDPNNILM-RHLKNLTDDE---FKC---IIHRSSDFL----YLSDRDYTSITKE-TLVSEIVEE--YPDD-CNK-ILAII--FL-----------VLDKDID-VDIET-----------

>Horsepox

------MVKNKISNSCLMIMSTDPNNILM-RHLKNLTDDE---FKC---IIHRSSDFL----YLSNRDYTSITKE-TLVSEIVEE--YPDD-CNK-ILAII--FL-----------VLDKDID-VDIKT-----------

>Orthopox abatino

MVKNNKIQKIKTSNSYRDVIMTNPNNILM-RHLKNLTDDE---FKC---IVNRSSDFI----YLSDRDYTSITKE-TLVSEIVEE--YPDD-CNK-ILAII--FL-----------VLGKDVD-IETET-----------

>Akhmeta virus

MVKSNKIQKNKISNSCRYMIMNTNTNILM-RHLKNLTDDE---FKC---IIHRSSDFL----YLSDRDYTSITKE-TLVSEIVEE--YQDD-CNK-ILAII--FL-----------VLDKDID-VDIET-----------

>Variola Virus

-----------------MIMSTDPNNILM-RYLKNLTDDK---FKC---IIHQSSDFL----YLSDRDYTSITKE-TLVSEIVEE--YPDD-CNK-ILAII--FL-----------VLDKDID-VDIET-----------

>Monkeypox

-----------------MIMRTDTNNILM-RHLKNLTDDE---FKC---IIHRSSDFL----YLSDRDYTSITKE-TLVSEIVEE--YPDD-CNK-ILAII--FL-----------VLDKDID-VDIET-----------

>Alaskapox

-----------------------MNTILM-RHFKNLTDDE---FKC---IIHRSSDFI----YLSDRDYTSITKE-TLVSEIVEE--YPDD-CDK-ILAII--FL-----------VLGKDIE-VDVKT-----------

>Volepox

---------------------MANKNRLV-SYLKNLTDDE---FKC---IIYRSSDFI----YLSDSDHDTITKE-KLVKEIIEE--YPDD-CNK-ILAII--FL-----------VLDKDID-IDIET-----------

>Skunkpox

----------------------MNKNRLV-KYLKNLTDDE---FKC---IIYRSSDFI----YLSDSDHDNITRE-TLVKEIMEE--YPDD-CNK-ILAII--FL-----------VLDKDID-LDIET-----------

>Raccoonpox

---------------------MDVKNRIV-KYLKNLTDDE---FRY---IIYRSSDFI----YLSDGDHNTITKE-TLVNEIIEE--YPDD-CNK-IFAII--FL-----------VLDIDID-LDTET-----------

>NY 014 poxvirus

------------------------MDKIM-CYLNNITDEE---FET---VIDRSSNFL----YLSDDDHTNITRE-SLALEIVEE--YPND-CER-ILAII--LL-----------SLDKYIS-FESTI-----------

>Murmansk poxvirus

------------------------MDKIT-HYLNNITDGE---FET---VIDRSSNFL----YLSDDDHTNITRE-SLALEIVEE--YPND-CER-ILAIL--LL-----------SLDKYIS-FESTI-----------

>Yoka poxvirus

------------------------MNKIL-NYFNSITDDE---FKT---IISISSDFL----YLSDNDNINITKE-SLALEILEQYPYPND-YEK-ILSML--LL-----------ALGKYVF-FETII-----------

>QDJ94995.1 IFN-inducible protein Hypsugopox virus

-------------------MDSYTKGSIL-YALKNLTFQQ---LKE---FMFISSEQL----YLSNKDRLNFDVV-DLADRIIIV--CHDN-ALK-AFKLI--KE-----------ILTMMES-VDFVINYMNKCIENTC

>QHG62576.1 hypothetical protein Cetacean poxvirus 1

-----------MIDSKSLNVEIEVKIALM-RALDELSSYQ---LKV---LAFLTSNRV----CVIHRSMSNVDRV-DIAKRINTT--LP---CVC-SLTYLRDLLKNVKHTESVRDILCEDINYIESIMRNNNEIKRFTC

>YP 009407969.1 IFN-inducible protein Eptesipox virus

-------------------MDAFTKGSTL-YALKNLTFNQ---LKE---FMFISSDGL----YLTHKNRLEFDVV-DLADRIIIV--NNNN-PLK-VFKHI--KE-----------TLCLMDD-VEFVLKYIDKCIENCA

Poxvirus DNA Polymerase Alignment

>MT712273.1:55688-58654 Teiidae poxvirus 1 isolate 1642/19, complete genome

ATGGATATCAGGTGCGTGAACTGGTTTGAGAACAAGGGCGACGTTAGATACATATATCTTAAAGCTCTGAAAAAAGATTCCGGAGTAATCTTTATCAGATTTAATCATTACTACTATTACGTATACGACGACTCT------AAAGAACTCGAGTATACGCCTATAGAAACTATACCTTTAGGCGACTTCGAAGTGATAAATATTAACGAAACAATATGCGAAGAAGTAGAAGACATAAGGCAAACTGAATCGTGTAAAAAAAGTTTATGTCTCGTTAAGGAT---AATAAACGAAATAGAGAAGCGGGTTATCTTCACGAGTATATGGACATATCTTGGTTTTATCTGATTAATAATATATCACCCGACGGATGCTACCGGATAGACCCTGAACAGCTAATCGCTGTAAGAAAAGATTGCTACCATTGCAATAATATTAAACAAACTTTTCTCGAAGAAATTCCTATATTCGAAGTACAACTTACTTATTTATTCTTTGACATAGAGTGTCAGTTTAACAAAAAGTTTCCCTCCGTCTTCGTGAATCCGATATCGCATATCAGTTGTTGTATTATAGAT---ACGGTAAAGGAATATAAGTTTAGTTTAATCAATACCGATTTATTGGAGAGGCAAGAGAATACC---------------------------GTAAATAATTGTAAAGATTTTTCACCCGACGTAGCTATAACTTTTTGCGAAGAAATTGTTCTTCTTCATATAATGAAAAGAATTCTCGAACATCGATTTGACTTCGTCGTTACTTTTAACGGGAATAACTTCGATATCAGATATATTTCTGGAAGACTAGAGATACTGGATAAAACTTTTATATATTTTACGTTACCCGATAATACCGAAAGCGTTAAACTCAGAATATTCGAACGCTATCAGGCCGGA------------GGTGCATTCGTTAATAAGACTTATCATATTAATAATAACAACGGCGTATTCTTTTTTGACCTATACACGTTTATACAACGAACTGAGCGTATGGACTCTTATAAGTTAGACAGTATTTCTAAAAATATTTTTAACTGCAACGGCGTGATTACGATGGAAGAAAATAATGAAGTATCTATTACCGCTACAGCGAAAGAAAACAACAAAGATAAACTCTCCATCTTCTGCAACGTCTTGGAAACAGGAAACTACATAACTATAGGAAATAACGACGTAGTTAAAATCATAAACAAAGATATCAGATCGGACGGATTTACCGTTCGTGTAATTACG---AGTAGATCGTATACGGACGGGGAAGTACAACTCATAAGTTTTGGCAAGGATGACGTAGATCTAAAGTCTATGTACAAAAACTATAATAGCGATATAGCTTTAGACATGGAAAAATATTGCATGCACGACGCTTGTCTTTGTAAACACATATGGAACTATTATAGAATACCGAGTAAGATAAACGCGGCTTCGTCTACTTATTTGTTACCGCAAAGTTTAGCTTTAGAATACAGAGCTAGTACACTTATAAAAGGTCCTCTACTGAAACTTATGTTAACAGAGAATATAATATATAATCGTGTAGGAGCAAAAATAAAATATCCTTATATCGGTGGAAAAGTATTTCTTCCTTCACAAAAGACTTTTGAGAACAACATTATGGTATTTGATTATAACAGTTTGTACCCTAACGTATGCATTTACGCCAATTTATCTCCCGAGAAATTAGTGTGCGTGATATTAAATCGAAATAAATTAGACGCGGAAATAAATCTCAGAAAATTAAAACGTAAATATCCATACCCGGATTATGTAAGCGTCACTTGCGATCCGAGAATCGTAGGATACTACAATGAAATAATAGTGTACGATCGTAGAGAAAAAGGGATAATCCCTAAACTTCTGGAGCTTTTCATAGAAAAGAGAAAGAAGTATAAATCTCTTCTGCGTGACGCGGAGAATGCCTTAGAGATTACTCTCTACGATTCTCTACAATATATATATAAAATAATAGCAAACTCCGTTTACGGTTTAATGGGATTCAGTAACAGTACGCTATATTCTTATTCTTCTGCTAAAACATGTACTACTATAGGTAGAAACATGATCACCTACTTAGATTCTATTATGAACGGAGCCGTCTGGAAAAACGATAAACTTATATTCGCAGAATTTCCTAATAACATATTTTCTTGCGAGGTTATATATAACAAAGAAATACAAGTGCAGCAGATGGACGATACT---------------------TTTCGCTTTAGAAACGTATACGGTGATACAGATTCCATATTCTTAGAAATATCAAGTAAAGATATACTAAAGACGATAACTATAGCGAAGATTTTGGAACACGTTATTAACACTAAGGTATTGTACGCGAATTTTAGAATAGAGTTTGAAGCGGTATATACTCAACTCATTCTGCAGTCGAAAAAGAAGTACACGACGATAAAATACTTAGCGGATTATAAGCCGGGTAATAAACCTATTAGGGTAAACAAAGGAACGAGCGAAACGCGCAGAGACGTCGCGTTGTTTCATAAACAGATGATACAAAAATACAAAGAGTTACTGATGAAATTGTTATCAGAGAGCGAA---TGTCAGCAAGAGATAACGAAACAAATACTTCAGCGTTTAGAGGTGGATCTGTTAACGGAGTTT---AAACAGAATACGGAATTTGATAAATACTTGTTGAGTAGGAAACACCACAATAATTATAAGTCGGCTACTCACTCTAATTTTGAGCTGGTTAAAGCTTACAATTTAGAGAATACTGAGAAAATAGAAATAGGCGAAAGATATTTCTACCTCTACATGTGCGACGCAAAAATGGTTTGGCAAAAAAAAATCTGCAACATACTCTCGTACGAGGCAATCGTAGACAGTAAATTTAAATTACCCGAAGACAAAAGAATTTTCTACGAAGTATACTTTAAGAGAATAGCGTCCGAGATGGTTAACCTGCTTACGGATAAAACTCAGTGTACTCTTTTCTTTCAGCGGTTATTCGGTGTAAGACCCATATACGTATCATAC

>NC_028238.1:70062-73028 Turkeypox virus strain TKPV-HU1124/2011, complete genome

ATGGATATCAGATGCGTTAACTGGTTCGAGAACAAAGGTGATATAAAATACATATATTTAAAAGCTATAACTAAATCATCTACGGTAATATTTATTAGATTTGATTATAACTATCATTATGTATATGACACCGAT------ACAGAATTAGATTTTACGCCTATAGATTCGTCTGAACTGGGACAATTCAATATTATTGATATTGACGAAATTGTTGATAAAGATATACGCGATGTTGTTGATAGAAAGACGTACACTAAACATTTACGGCTTGTAAAGGAT---AACAGAAAGAATAGACAAAAAGGATACTTGAGTGAATATTTAGATATTACGTGGTTTTATTTGTTAAATTCTATAAAACCAGATGGTTGTTATGAAATAAATATGGAAAAGCTTTCAGCAATAAGCAGAGATTGTTATCATTGCAAAGAACCTAATAAATTATTTACAAAAGAAATACCGTTATTTGATATAAAATATACATATTTATGTTTCGATATCGAATGCCAATTTGATAAGAAGTTTCCTTCTGTATTCGTTAATCCTATATCACATATTAGTTGTTTGATAATAGAT---ACAAAACGCGAATATAAATTTAGCCTTATCAATACGGATCTGTTAGACGACAAATCACCTACT---------------------------ATTAATCACCATAATGATTTCTCTCCTGCTACCGGTTTAACATTTTGTACTGAGATTGTAATGTTAAATATAATGAAGAGAATATTAGAGCATAGGTTTGATTTTATAATAACTTTCAATGGTAATAATTTCGATATTAGATATATTACTGGAAGATTAGAAATATTAGAAAAAAAATTCATATATTTTAGCCTTCCTGATAAATCGGAAACTATAAAGTTGAAAATATTTGAAAGATTCCAATCCGGT------------GGTACTTTTACAAACAAGACGTATCATATCAACAATAATAATGGCGCGATTTTCTTTGACTTATATGCTTTTATACAAAAAACTGAGCGATTAGAGTCATATAAATTAGATAATATTTCTAAGAATATCTTTAATTGTATAGGTACCATTAAAGATATGTCTGGTAATATATTAACCATAGAGGCAAATACTATAGAAAACTCTAAAGATAAACTAGACATATTTATTACAGTATTATCTACAGGTAACTATATAACAATAGATAACTTAGAAATAACGGAGATATTGGACAAGAACATACAACCCGATAAATTTGTAATAAAAGTATATAGT---AATAAACAATATGATATAAATACATGTCACTTAATAAGTTTCGGTAAGGACGATGTAGATTTAAAGCAGATGTATAACAATTATAACTTAGAAACGGCTATTAAAATGGAAAAATATTGTATTCACGATGCGTGTTTATGTAAATATATATGGGATTATTATAGAGTACCTAGCAAAATAAACGCAGCTTCCTCTACTTATTTGTTACCACAATGTCTGGCTCTAGAATATAGAGCTAGTACCCTTATAAAAGGTCCTTTGTTAAAGTTGTTACTAGATGAACGTATAGTATACCAACGTGTTAATTCGAAGGTTAAATACCCCTATATAGGCGGAAAAGTATTCATGCCTTCTCAGAAAACTTTTGAGAATAATGTAATGATATTCGACTATAATAGCTTATATCCAAATGTATGTGTTTATGCAAATCTTTCTCCAGAAACATTAGTATGTGTTGTTCTAAGTTCTAATAAACTTGAATCTGAGATAAACATTAAAACTATAAAGGCAAAATATCCATATCCTGATTATATATATGTTATATGCGAATCAAGATTAAAAGGATATTATAATGAAATAGTAGTTTACGATAGAAGGAAGGAAGGTATTATTCCTAAATTGCTCAATATATTCATGCTAAAAAGAAAAACGTATAAAAAATTGCTAAAAGACGCGACTACTACTATAGAGACAGCGTTATACGATTCTCTTCAATATATTTACAAGATAATAGCTAATTCAGTTTATGGACTAATGGGGTTTAACAATAGTATATTATATTCTTATTCATCTGCAAAAGCATGTACAACAATAGGTAGAAATATGATAATGTACCTAGATTCTGTAATGAACGGTGCAGTATGGGAAAATGATAAACTTGTATTAGCAGACTTTCCGAGAAATATATTTTCAGGAGAAGTGATATTCTCTAAAGAAATACCGGTAACGCAAGTAGATGGGACT---------------------TTTAAATTCAGAAGTGTATACGGTGACACAGATTCTATATTTTCAGAAATATCTAGTAAAGATGTAGAAAAGACACTTGCTATAGCAAGGATACTGGAACAAGTAATAAATACAAAGGTATTATATGGAAACTTTAGGATAGAATTCGAAGCTATATATACACAGCTAATATTACAATCAAAGAAAAAATATACTACTATAAAATATTCCGCCGCTTACAAACCCGGTGACAAACCTATACGGATAAATAAAGGAACTAGCGAAACCAGAAGAGATGTTGCCCTATTCCATAAACACATGATACAGAAATATAAAGATCTTTTAATGAAAGCACTTATGGAAAGTGAT---ACAAAAAATGATATTACAAGAACAATACTACAACACTTAGAAACCGATATGGTTAAAGAATTC---TCGTACAATACAGACTTTGAAAAATACCTATTAAGCAGGAAACATCACAATAATTATAAATGCATAACCCATTCTAATTTTGAATTAGTGAAAAAATATAACATTGAAAATACAGAAAAGATAGAGATAGGAGAACGATATTTCTACATATATATATGTGACGCGTCGTTACCATGGCAAAAAAAACTATGTAACATTCAGTCATACGAAACTATAGCTGATAGCAAATTCAGTCTACCGTATAATAAAAGAATATTTTATGAAGTATATTTTAAAAGAATAGCTGCAGAAGTAGTAAATTTACTTCCTGATAAAACTATTTGTACGTTATTCTTCACAAGACTATTCGCTACTAAACCAACGTTTGCGTTAGAC

>OK348853.1:91721-94687 Albatrosspox virus strain TST/1997, complete genome

ATGGATATAAGGTGCGTAAACTGGTTTGAGAATAAAGGAGAAACAAAATATATTTACTTAAAAGCTATTAACCGAGAATCGAATGTTATATTTATAAGATTCAATTATTACTATCACTATGTATACGATGCTTCC------AAAGAACTAGAATATAAACCTAATGAGTGTATAGATTTAGGACCGTTCAAAATTATTAATATAGACGAAAAGCTAAGTACCGATATAAGGTATGTCGAACCTCGAAATTACTATACTTCGGAATTGGTACTCGTAAAGGAT---CTAAAAAGAAATAGGGAAAAACAATATCTGCAAGAATATTTAGATATAACTTGGTTTTATCTACTTAATAATATAACACCGGACGGGTGTTATAAAATAGATATAGAACATCTAACTCCTATAAAAAAAGATTGTTACCATTGTGATGATGTTAGCAAAGTATTCATTCAAGAAATACCTATCTTCGAAGTTAAATTTACTTACTTACTGTTTGACATAGAATGTCAATTTGATAAAAAGTTTCCTTCTGTATTTGTAAACCCTATTTCACATATCAGTTGTTGGATTATAGAC---AAGGTCACCGAATATAAGTTTACTTTAATTAATACAGATATCTTACCCGATAAAGAACCTAGT---------------------------ATATTACATCACAAAGACTTCTCTCCAAAAGATAGGATAACCTATTGTACAGAAATTGTGATGTTGCTTATAATGAAAAAAATTCTAGAACATAGATTCGATTTTGTAATAACTTTTAACGGAAATAATTTTGATATCAGGTATATATCTGGAAGGCTAGAAATTCTCGAGAAATCTTTTATATATTTCTCTCTTCCTGATGCGACGGAAACAGTTAAACTTAAAATATTTGAAAGATTCGTTACAGGA------------GGAACATTCACTAATAAAACATACCACATAAACAATAATAATGGTGTTATGTTTTTTGATTTGTATGCGTTCATACAAAAAACAGAACGATTAGATTCTTACAAACTAGATAGCATATCAAAAAATATATTTAATTGTAACGTTGCTATAAAAGAAATAGATGATACAATTTTAACATTGGAAGCCACGGTAAAAGATAATTCTAAAGATAAATTATCTATATTTTCTAGAGTATTAGAAACCGGTAATTATATCACTATAGGAGATAACAATGTAAGCAAAATAGTATACAAAGATATAAACCAAGATAGTTTCATAATTAAAGTCATATCT---AACAGGGATTACGAAATAGGATCGGTACATAATATAAGTTTTGGAAAGGACGATGTAGACTTAAAAGACATGTATAAAAACTATAATCTGGAAATAGCGTTAGATATGGAAAGATATTGTATTCACGACGCTTGTCTCTGTAAATATATATGGGATTATTACAGGGTGCCCAGTAAGATTAACGCCGCATCATCTACTTATCTTTTACCACAAAGCTTAGCGCTAGAATATAGGGCCAGTACTCTTATTAAAGGACCATTACTGAAGTTACTATTAGAAGAACGAGTAATCTATACTAGAAAAATCACAAAAGTAAGATATCCGTATATAGGTGGGAAGGTATTTCTTCCTTCTCAGAAAACTTTCGAGAATAATGTAATGATATTTGATTATAATAGTCTGTATCCAAATGTATGCATCTACGGTAATCTATCACCAGAAAAACTAGTATGTATATTATTAAATAGTAATAAGCTAGAATCAGAAATAAATATGAGAACTATCAAAAGTAAGTATCCATATCCTGAATATGTTTGTGTTTCTTGTGAATCTAGACTTTCAGATTATTATAGCGAAATTATTGTTTACGATAGAAGAGAAAAAGGTATAATACCTAAACTTTTGGAGATGTTTATAGGGAAGAGAAAAGAATATAAAAACCTTTTAAAGACAGCATCGACGACTATAGAAAGTACTTTGTATGACTCTTTGCAATATATCTATAAGATAATAGCAAACTCTGTTTACGGTTTAATGGGATTCAGTAATAGTACTCTATATTCTTATTCGTCAGCAAAGACGTGTACTACTATAGGTAGAAATATGATTACCTATCTAGATTCTATAATGAATGGCGCTGTGTGGGAAAACGATAAGCTTATTCTAGCAGATTTTCCTAGAAACATATTTTCAGGAGAAACAATGTTCAACAAAGAACTAGAAGTTCCTAACATGAATGAATCT---------------------TTTAAGTTTAGGAGCGTATACGGCGATACAGATTCTATATTTTCAGAGATATCTACCAAAGATATAGAGAAAACAGCCAAGATAGCAAAACACCTAGAACATATAATAAACACAAAAATATTACACGCTAACTTTAAAATAGAATTTGAAGCAATTTATACGCAATTGATATTACAGTCAAAGAAGAAATATACTACAATAAAGTATTTAGCGAACTACAAACCAGGGGACAAACCTATAAGAGTAAACAAAGGAACCAGCGAAACACGTAGAGACGTGGCATTGTTCCATAAACACATGATACAAAGATATAAAGATATGTTAATGAAGCTGTTAATGGAAAGCAAA---GGACAGCAAGAGATAACCAGATTAATTCTTCAAAGTTTAGAAACAGATATGATATCCGAATTT---ACACACAACAGAGAATTTGAAAAGTATTTGTTGAGTAGGAAACATCACAATAATTACAAATCAGCGACTCACTCAAATTTTGAACTTGTTAAAAGATACAATTTAGAGAATACAGAAAAAATAGAAATAGGAGAAAGATACTATTATATCTATATATGTGATATTAGTTTGCCATGGCAAAAAAAGCTATGCAATATATTATCCTATGAAGTAATCGCCGATAGCAAGTTTTATCTGCCTAAAGACAAAAGAATATTCTACGAAATATACTTTAAAAGAATAGCATCTGAAGTAGTAAATCTGCTAACGGATAAAACACAGTGTATGTTATTTTTCAGCAGACTTTTCGGTACTAAGCCTGTATTTTCATCAGAC

>NC_002188.1:93460-96426 Fowlpox virus, complete genome

ATGGATATAAGGTGCGTAAACTGGTTTGAGAATAAAGGAGAAACAAAATATATTTACTTAAAAGCTATTAACCGAGAATCGAATGTTATATTTATAAGATTCAATTATTACTATCACTATGTATACGATGCTTCC------AAAGAACTAGAATATAAACCTAATGAGTGTATAGATTTAGGACCGTTCAAAATTATTAATATAGACGAAAAGCTAAGTACCGATATAAGGTATGTCGAACCTCGAAATTACTATACTTCGGAATTGGTACTCGTAAAGGAT---CTAAAAAGAAATAGGGAAAAACAATATCTGCAAGAATATTTAGATATAACTTGGTTTTATCTACTTAATAATATAACACCGGACGGGTGTTATAAAATAGATATAGAACATCTAACTCCTATAAAAAAAGATTGTTACCATTGTGATGATGTTAGCAAAGTATTCATTCAAGAAATACCTATCTTCGAAGTTAAATTTACTTACTTACTGTTTGACATAGAATGTCAATTTGATAAAAAGTTTCCTTCTGTATTTGTAAACCCTATTTCACATATCAGTTGTTGGATTATAGAC---AAGGTCACCGAATATAAGTTTACTTTAATTAATACAGATATCTTACCCGATAAAGAACCTAGT---------------------------ATATTACATCACAAAGACTTCTCTCCAAAAGATAGGATAACCTATTGTACAGAAATTGTGATGTTGCTTATAATGAAAAAAATTCTAGAACATAGATTCGATTTTGTAATAACTTTTAACGGAAATAATTTTGATATCAGGTATATATCTGGAAGGCTAGAAATTCTCGAGAAATCTTTTATATATTTCTCTCTTCCTGATGCGACGGAAACAGTTAAACTTAAAATATTTGAAAGATTCGTTACAGGA------------GGAACATTCACTAATAAAACATACCACATAAACAATAATAATGGTGTTATGTTTTTTGATTTGTATGCGTTCATACAAAAAACAGAACGATTAGATTCTTACAAACTAGATAGCATATCAAAAAATATATTTAATTGTAACGTTGCTATAAAAGAAATAGATGATACAATTTTAACATTGGAAGCCACGGTAAAAGATAATTCTAAAGATAAATTATCTATATTTTCTAGAGTATTAGAAACCGGTAATTATATCACTATAGGAGATAACAATGTAAGCAAAATAGTATACAAAGATATAAACCAAGATAGTTTCATAATTAAAGTCATATCT---AACAGGGATTACGAAATAGGATCGGTACATAATATAAGTTTTGGAAAGGACGATGTAGACTTAAAAGACATGTATAAAAACTATAATCTGGAAATAGCGTTAGATATGGAAAGATATTGTATTCACGACGCTTGTCTCTGTAAATATATATGGGATTATTACAGGGTGCCCAGTAAGATTAACGCCGCATCATCTACTTATCTTTTACCACAAAGCTTAGCGCTAGAATATAGGGCCAGTACTCTTATTAAAGGACCATTACTGAAGTTACTATTAGAAGAACGAGTAATCTATACTAGAAAAATCACAAAAGTAAGATATCCGTATATAGGTGGGAAGGTATTTCTTCCTTCTCAGAAAACTTTCGAGAATAATGTAATGATATTTGATTATAATAGTCTGTATCCAAATGTATGCATCTACGGTAATCTATCACCAGAAAAACTAGTATGTATATTATTAAATAGTAATAAGCTAGAATCAGAAATAAATATGAGAACTATCAAAAGTAAGTATCCATATCCTGAATATGTTTGTGTTTCTTGTGAATCTAGACTTTCAGATTATTATAGCGAAATTATTGTTTACGATAGAAGAGAAAAAGGTATAATACCTAAACTTTTGGAGATGTTTATAGGGAAGAGAAAAGAATATAAAAACCTTTTAAAGACAGCATCGACGACTATAGAAAGTACTTTGTATGACTCTTTGCAATATATCTATAAGATAATAGCAAACTCTGTTTACGGTTTAATGGGATTCAGTAATAGTACTCTATATTCTTATTCGTCAGCAAAGACGTGTACTACTATAGGTAGAAATATGATTACCTATCTAGATTCTATAATGAATGGCGCTGTGTGGGAAAACGATAAGCTTATTCTAGCAGATTTTCCTAGAAACATATTTTCAGGAGAAACAATGTTCAACAAAGAACTAGAAGTTCCTAACATGAATGAATCT---------------------TTTAAGTTTAGGAGCGTATACGGCGATACAGATTCTATATTTTCAGAGATATCTACCAAAGATATAGAGAAAACAGCCAAGATAGCAAAACACCTAGAACATATAATAAACACAAAAATATTACACGCTAACTTTAAAATAGAATTTGAAGCAATTTATACGCAATTGATATTACAGTCAAAGAAGAAATATACTACAATAAAGTATTTAGCGAACTACAAACCAGGGGACAAACCTATAAGAGTAAACAAAGGAACCAGCGAAACACGTAGAGACGTGGCATTGTTCCATAAACACATGATACAAAGATATAAAGATATGTTAATGAAGCTGTTAATGGAAAGCAAA---GGACAGCAAGAGATAACCAGATTAATTCTTCAAAGTTTAGAAACAGATATGATATCCGAATTT---ACACACAACAGAGAATTTGAAAAGTATTTGTTGAGTAGGAAACATCACAATAATTACAAATCAGCGACTCACTCAAATTTTGAACTTGTTAAAAGATACAATTTAGAGAATACAGAAAAAATAGAAATAGGAGAAAGATACTATTATATCTATATATGTGATATTAGTTTGCCATGGCAAAAAAAGCTATGCAATATATTATCCTATGAAGTAATCGCCGATAGCAAGTTTTATCTGCCTAAAGACAAAAGAATATTCTACGAAATATACTTTAAAAGAATAGCATCTGAAGTAGTAAATCTGCTAACGGATAAAACACAGTGTATGTTATTTTTCAGCAGACTTTTCGGTACTAAGCCTGTATTTTCATCAGAC

>NC_036582.1:95291-98260 Flamingopox virus FGPVKD09, complete genome

ATGGATATAAGGTGCGTAAATTGGTTTGAGAACAAAGGAGAAATAAAATATATTTACTTAAAAGCTATTAACAGAGAATCGAATGTTGTATTTATAAGGTTCAATTATTACTATCACTATGTATACGATGCTTCA------AAAGAACTAGAATATAAACCTAAAGAACGTATAGATTTAGGAAAGTTCAAAATCATTAATATAGACGAAAAACTAAATACCGATATAAGATATGTTGAACAACGAGATTATTATACTTCAGAATTAGTACTCGTAAAGGAT---CTAAAAAGAAATAGAGAAAAACAATATCTACAGGAATATTTAGATATAACCTGGTTTTATCTACTTAATAATATAACACCAGACGGGTGTTATAAAATAGATATAGAACATCTAACTCCTATAAAAAAAGATTGTTATCATTGTGATGATGTTAGCAAAGTATTCATTCAAGAAATACCGATATTTGAAGTTAAATTTACTTACTTACTGTTTGACATAGAATGTCAATTTGATAAAAAGTTTCCGTCTGTATTTGTAAACCCTATTTCACACATCAGTTGTTGGATTATAGAT---AAAGTTACTGAATATAAATTTACTTTAATCAATACAGATCTATTACCGGATAAAGAGCCTAGT---------------------------ATATTACATCACAAAGATTTCTCTCCAAAAGACAGAATAACTTACTGTACAGAAGTTGTGATGCTACTTATAATGAAAAAAATTCTAGAACATAGATTCGACTTTGTAATAACTTTTAATGGAAATAATTTTGATATCAGGTATATATCTGGAAGGTTAGAAATTCTAGAGAAATCTTTTATATATTTCTCACTTCCTGATGCTACAGAAACAGTTAAACTTAAAATATTTGAAAGATTTGTTACCGGT------------GGAACATTCACTAATAAAACATATCACATAAACAATAATAATGGTGTCATATTTTTTGATTTGTATGCTTTCATACAAAAAACAGAACGATTAGATTCTTACAAACTAGATAGTATATCTAAAAATATATTTAATTGTAACATTACTATAAAAGAAATAGATGACACAGTTTTAACATTGGTAGCTACGGTAAAAGATAATTCTAACGATAAATTATCTATATTTTCTAGAGTGTTAGAAACCGGTAATTATATCACTATAGGAAATAACGACGTAAGCAAAATAATATATAAAGATATAAACCAAGATAGTTTTATAATTAAAGTTATATCTAATAACAGAGATTATGAAGTAGGGTCGTTACATAATATAAGTTTTGGAAAGGATGATGTTGACTTAAAAGATATGTACAAAAACTACAATCTGGAAATAGCGTTAGATATGGAAAGGTATTGTATTCATGATGCATGTCTCTGTAAATACATATGGGATTATTACAGAGTACCCAGTAAGATTAACGCCGCATCTTCTACTTATCTTTTACCGCAAAGCTTAGCATTAGAATATAGGGCCAGTACTCTTATTAAAGGACCATTACTGAAGTTACTATTGGAAGAACGAGTAATTTATACTAGAAAAATCACAAAGGTAAGATATCCGTATATAGGTGGAAAGGTATTTCTTCCTTCTCAGAAAACTTTCGAAAATAATGTAATGATATTCGATTATAATAGTCTGTATCCAAATGTATGCATCTATGCTAATCTATCACCAGAGAAACTAGTGTGTATAGTGTTAAATACTAATAAACTGGAAGCAGAAATAAATATGAGATCAATCAAAAGTAAGTTCCCATATCCTGATTATGTTTGTATCTCGTGTGAATCTAGACTATCTGATTATTATAGCGAGATTATAGTTTACGATAGAAGAGAGAAAGGTATAATACCTAAGCTTTTAGAGATGTTCATAGGAAAAAGAAAAGAGTATAAAAACCTTTTAAAGACAGCATCGACAACTATAGAAAGTACTTTGTACGATTCCTTGCAATATATCTATAAGATAATAGCAAACTCTGTTTACGGTTTAATGGGATTTAGTAACAGTACTTTATATTCCTATTCTTCCGCAAAGACGTGTACTACTATAGGTAGAAACATGATTACCTATCTAGATTCTATAATGAATGGTGCTGTGTGGGAAAACGATAAGCTTATCCTAGCAGATTTTCCTAGAAATATATTTTCAGGCGAAACAATGTTCAACAAAGAATTGTCAGTTCCTCAGATGAATGAATCT---------------------TTTAAGTTCAGGAGCGTATACGGAGATACAGATTCTATATTTTCGGAGATATCTACCAAAGATATAGAGAAAACAGCCAAGATAGCAAAACACCTAGAACATATAATAAATACAAAAATATTACACGCTAACTTTAAAATAGAATTCGAAGCAATTTATACACAATTGATATTACAATCAAAGAAGAAATATACTACAATAAAGTATTTAGCGAACTACAAACCAGGTGACAAACCTATAAGAGTAAACAAAGGAACCAGCGAAACACGTAGAGACGTAGCGTTGTTTCACAAACACATGATACAAAGATATAAAGATATGTTAATGAAGCTGCTAATGGAAAGCAAA---GGACAGCAAGAAATAACTAGACTAATTCTTCAAAGTTTAGAAACAGATATGGTAACCGAGTTT---ACTCATAATAGGGAATTTGACAAATATTTATTGAGTAGGAAACATCACAATAATTATAAATCAGCGACTCACTCAAATTTTGAACTAGTTAAAAGATACAATCTAGAGAATACAGAAAAGATAGAAATAGGAGAAAGATACTTTTATATCTATATATGTGATATTAGTTTACCATGGCAAAAAAAACTATGTAATATATTGTCCTATGAAGTAATCGCTGATAGTAAGTTTTCTCTACCTAAAGACAAAAGAATATTCTATGAAATATATTTTAAAAGAATAGCGTCTGAAGTAGTAAATCTACTAACGGATAAAACACAATGTACATTATTTTTCAGCAGACTTTTCGGTACTAAGCCTGTATTTTCATCAGAC

>OP292971.1:108285-111254 Cooks petrelpox virus strain Petrel/Aus/2022, complete genome

ATGGATATAATGTGTGTAAACTGGTTTGAGAACAAAGGAGAAATAAAATATATTTACTTAAAAGCTATTAACAGAGAATCGAATGTTGTATTTATAAGGTTCAATTATTACTATCACTACGTATACGATGCTTCA------AAAGAACTAGAATATAAACCTAAAGAACGTATAGATTTAGGAAAGTTCAAAATCATTAATATAGACGAAAAACTAAATACCGATATAAGATATGTTGAACAACGAGATTATTATACTTCAGAATTAGTACTCGTAAAGGAT---CTAAAAAGAAATAGAGAAAAACAATATCTACAGGAATATTTAGATATAACTTGGTTTTATCTACTTAATAATATAACACCAGACGGGTGTTATAAAATAGATATAGAACATCTAACTCCTATAAAAAAAGATTGTTATCATTGTGATGATGTTAGCAAAGTATTCATTCAAGAAATACCGATATTTGAAGTTAAATTTACTTACTTACTGTTTGATATAGAATGTCAGTTTGATAAAAAGTTTCCGTCTGTATTTGTAAACCCTATTTCACACATCAGTTGTTGGATTATAGAT---AAAGTTACTGAATATAAATTTACTTTAATCAATACAGATCTATTACCGGATAAAGAGCCTAGT---------------------------ATATTACATCACAAAGATTTCTCTCCAAAAGACAGAATAACTTACTGTACAGAAGTTGTGATGCTACTTATAATGAAAAAAATTCTAGAACATAGATTCGACTTTGTAATAACTTTTAATGGAAATAATTTTGATATCAGGTATATATCTGGAAGGTTAGAAATTCTAGAGAAATCTTTTATATATTTCTCACTTCCTGATGCTACAGAAACAGTTAAACTTAAAATATTTGAAAGATTTGTTACCGGT------------GGAACATTCACTAATAAAACATATCACATAAACAATAATAATGGTGTCATATTTTTTGATTTGTATGCTTTCATACAAAAAACAGAACGATTAGATTCTTACAAACTAGATAGTATATCTAAAAATATATTTAATTGTAACATTACTATAAAAGAAATAGATGACACAGTTTTAACATTGGAAGCTACGGTAAAAGATAATTCTAACGATAAATTATCTATATTTTCTAGAGTGTTAGAAACCGGTAATTATATCACTATAGGAAATAACGACGTAAGCAAAATAATATATAAAGATATAAACCAAGATAGTTTTATAATTAAAGTTATATCTAGTAACAGAGATTATGAAGTAGGGTCGTTACATAATATAAGTTTTGGAAAGGATGATGTTGACTTAAAAGATATGTACAAAAACTACAATCTGGAAATAGCGTTAGATATGGAAAGGTATTGTATTCATGATGCATGTCTCTGTAAATACATATGGGATTATTACAGAGTACCCAGTAAGATTAACGCCGCATCTTCTACTTATCTTTTACCGCAAAGCTTAGCATTAGAATATAGGGCCAGTACTCTTATTAAAGGACCATTACTGAAGTTACTATTGGAAGAACGAGTAATTTATACTAGAAAAATCACAAAGGTAAGATATCCGTATATAGGTGGAAAGGTATTTCTTCCTTCTCAGAAAACTTTCGAAAATAATGTAATGATATTCGATTATAATAGTCTGTATCCAAATGTATGCATCTATGCTAATCTATCACCAGAGAAACTAGTGTGTATAGTGTTAAATACTAATAAACTGGAAGCAGAAATAAATATGAGATCAATCAAAAGTAAGTTCCCATATCCTGATTATGTTTGTATCTCGTGTGAATCTAGACTATCTGATTATTATAGCGAGATTATAGTTTACGATAGAAGAGAGAAAGGTATAATACCTAAGCTTTTAGAGATGTTCATAGGAAAAAGAAAAGAGTATAAAAACCTTTTAAAGACAGCATCGACAACTATAGAAAGTACTTTGTACGATTCCTTGCAATATATCTATAAGATAATAGCAAACTCTGTTTACGGTTTAATGGGATTTAGTAACAGTACTTTATATTCCTATTCTTCCGCAAAGACGTGTACTACTATAGGTAGAAACATGATTACCTATCTAGATTCTATAATGAATGGTGCTGTGTGGGAAAACGATAAGCTTATCCTAGCAGATTTTCCTAGAAATATATTTTCGGGCGAAACAATGTTCAACAAAGAATTGTCAGTTCCTCAGATGAATGAATCT---------------------TTTAAGTTCAGGAGCGTATACGGAGATACAGATTCTATATTTTCGGAGATATCTACCAAAGATATAGAGAAAACAGCCAAGATAGCAAAACACCTAGAACATATAATAAATACAAAAATATTACACGCTAACTTTAAAATAGAATTCGAAGCAATTTATACACAATTGATATTACAATCAAAGAAGAAATATACTACAATAAAGTATTTAGCGAACTACAAACCAGGTGACAAACCTATAAGAGTAAACAAAGGAACCAGCGAAACACGTAGAGACGTAGCGTTGTTTCACAAACACATGATACAAAGATATAAAGATATGTTAATGAAGCTGCTAATGGAAAGCAAA---GGACAGCAAGAAATAACTAGACTAATTCTTCAAAGTTTAGAAACAGATATGGTAACCGAGTTT---ACTCATAATAGGGAATTTGACAAATATTTATTGAGTAGGAAACATCACAATAATTATAAATCAGCGACTCACTCAAATTTTGAACTAGTTAAAAGATACAATCTAGAGAATACAGAAAAGATAGAAATAGGAGAAAGATACTTTTATATCTATATATGTGATATTAGTTTACCATGGCAAAAAAAACTATGTAATATATTGTCCTATGAAGTAATCGCTGATAGTAAGTTTTCTCTGCCTAAAGACAAAAGAATATTCTATGAAATATATTTTAAAAGAATAGCGTCTGAAGTAGTAAATCTGCTAACGGATAAAACACAATGTACATTATTTTTCAGCAGACTTTTCGGTACTAAGCCTGTATTTTCATCAGAC

>NC_024447.1:100701-103667 Pigeonpox virus isolate FeP2, complete genome

ATGGATATAAGGTGTGTAAACTGGTTTGAGAACAAAGGAGAAATAAAATATATTTACTTAAAAGCTATTAACAGAGAATCGAATGTTGTATTTATAAGGTTTAATTATTACTATCACTATGTATACGACGCTTCA------AAAGAACTAGAATATAAACCTAAAGAACGTTTAGATTTAGGAAAGTTCAAAATCATTAATATAGATGAAAAACTAAATACCGATATAAGATATGTTGAACAACGAGATTATTATACTTCAGAATTAGTACTCGTGAAGGAT---CTAAAAAGAAATAGAGAAAAACAATATCTACAGGAATATTTAGATATATCCTGGTTTTATCTACTTAATAATATTACTCCGGACGGGTGTTATAAAATAGATATAGAACATCTAACTCTTATAAAAAGAGATTGTTATCATTGTGATGATGTTAGCAAAGTATTCATTCAAGAAATACCTATATTTGAAGTTAAATTTACTTACTTACTGTTTGACATAGAATGTCAATTTGATAAAAAGTTTCCGTCTGTATTCGTAAACCCTATTTCACACATCAGTTGTTGGATTATAGAT---AAGGTTACTGAATATAAATTTACCTTAATCAATACAGATCTATTACCGGATAAAGAGCCTAGT---------------------------ATATTACATCACAAAGATTTCTCTCCAAAAGACAGAATAACTTACTGTACAGAAGTTGTGATGCTACTTATAATGAAAAAAATTCTAGAACATAGATTCGACTTTGTAATAACTTTTAACGGAAATAATTTCGATATTAGGTATATATCTGGAAGGCTAGAAATTCTAGAGAAATCTTTTATATATTTCACATCTCCCGATGCTACAGAAACAGTCAAACTTAAAATATTTGAAAGATTTGTTACCGGT------------GGAACTTTCACTAATAAAACATATCACATAAACAATAATAATGGTGTCATATTTTTTGATTTGTATGCTTTCATACAAAAAACAGAACGATTAGATTCTTACAAACTAGATAGTATATCTAAAAATATATTTAATTGTAACATTACTATAAAAGAAATAGATGACAGGGTTTTAACATTGGAAGCCACGGTAAAAGATAATTCTAACGATAAATTATCTATATTTTCTAGAGTATTGGAAACCGGTAATTATATCACTATAGGAAATAATGACGTAAGTAAAATAATATATAAAGATATAAACCAAGATAGTTTTATAATTAAAGTTATATCTAATAACAGAGATTATGAAGTAGGGTCGTTACATAATATAAGTTTTGGAAAGGATGATGTTGACTTAAAAGTTATGTACAAAAACTACAATCTGGAAATAGCGTTAGATATGGAAAGGTATTGTATTCATGACGCGTGTCTCTGTAAATACATATGGGATTACTACAGAGTACCCAGTAAGATTAACGCCGCATCTTCTACTTATCTTTTACCACAAAGCTTAGCATTAGAATATAGGGCCAGTACTCTTATTAAAGGACCATTACTGAAGTTACTATTGGAAGAACGAGTAATCTATACTAGAAAAATCACAAAGGTAAGATATCCGTATATAGGTGGAAAGGTATTTCTTCCTTCTCAGAAAACTTTCGAAAATAATGTTATGATATTCGATTATAATAGTCTGTATCCAAATGTGTGCATCTATGCTAATCTATCACCAGAGAAATTAGTGTGTATAGTGTTAAATACTAATAAACTAGAAGCAGAAATAAATATGAGATCAATCAAAAGTAAGTTCCCATATCCCGATTATGTTTGTATCTCATGTGAATCTAGACTATCTGATTATTATAGCGAGATTATAGTTTACGATAGAAGAGATAAAGGTATAATACCTAAGCTTTTAGAGATGTTCATAGGAAAAAGAAAAAAGTATAAAAACCTTTTAAAGACAGCATCGACAACTATAGAAATTACTTTGTACGATTCCTTGCAATATATCTATAAGATAATAGCAAACTCTGTTTATGGTTTAATGGGATTTAGTAACAGTACTTTATATTCCTATTCTTCCGCAAAGACGTGTACTACTATAGGTAGAAACATGATTACCTATCTAGATTCTATAATGAATGGTGCTGTATGGGAAAACGATAAGCTTATCCTAGCAGATTTTCCTAGAAATATATTTTCTGGAGAAACCATGTTTTCTAAAGAATTAAAAGTAGAACAGATGAACGAAACG---------------------TTTAAATTTCGAAACGTTTACGGAGATACAGATTCCATATTTACAGAAATATCTAATAGAGATGTAGATAAAACCATAAAAATAGCAAAACACCTAGAAAATATAATTAATACAAAAATATTATATGATAACTTTAAAATAGAATTCGAAGCTGTTTATACACAATTAATTTTACAGTCAAAGAAAAAATACACAACTATAAAGTATTTAGCAAATCATAAGCCAGGTGATAAGACTATAAGAATTAATAAAGGGACTAGTGAAACACGTCGAGACGTAGCATTATTTCATAAACAGATGATACAAAAATATAAAGACTTATTAATGAAAATGTTACTAGAAGGAAAA---GGA---AAAGATATAACTAGATTAATTCTTCAAAGTTTAGAAACAGATATGGTAACCGAGTTT---ACTCATAACAGGGAATTTGATAAATACTTATTAAGTAGGAAACATCACAATAATTATAAATCAGCTACTCACTCAAATTTTGAACTAGTTAAAAGATACAATCTAGAGAATACAGAGAAGATAGAAATAGGAGAAAGATACTTTTATATCTACATATGTGATATTAGTTTACCGTGGCAAAAAAAGCTATGTAATATATTATCCTATGAAGTAATCGCCGATAGTAAATTTTCTCTACCTAAAGACAAAAGAATATTCTATGAAATATATTTTAAAAGAATAGCGTCTGAAGTAGTAAATCTGCTAACGGATAAAACACAATGTACAGTATTTTTCAGCAGGCTTTTCGGTACTAAGCCTGTATTTTCATCAGAC

>NC_024446.1:101802-104768 Penguinpox virus isolate PSan92, complete genome

ATGGATATAAGGTGTGTAAACTGGTTTGAGAACAAAGGAGAAATAAAATATATTTACTTAAAAGCCATTAACAGAGAATCGAATGTTGTATTTATAAGGTTCAATTATTACTATCACTATGTATACGATGCTTCA------AAAGAACTAGAATATAAACCTAAAGAACGTATAGATTTAGGAAAGTTCAAAATCATTAATATAGACGAAAAACTAAATACCGATATAAGATATGTTGAACAACGAGATTATTATACTTCAGAATTAGTACTTATAAAGGAT---CTAAAAAGAAATAGAGAAAAACAATATCTACAAGAATATTTAGATATAACCTGGTTTTATCTACTTAATAATATAACACCAGACGGGTGTTATAAAATAGATATAGAACATCTAACTCCTATAAAAAAAGATTGTTATCATTGTGATGATGTTAGCAAAGTATTCATTCAGGAAATACCGATATTTGAAGTTAAATTTACTTATTTACTGTTTGACATAGAATGTCAATTTGATAAAAAGTTTCCGTCTGTATTTGTAAACCCTATTTCACATATCAGTTGTTGGATTATAGAT---AAGGTTACTGAATATAAATTTACTTTAATCAATACAGATCTATTACCGGATAAAGAGCCTAGT---------------------------ATATTACATCACAAAGATTTCTCTCCAAAAGACAGGATAACTTACTGTACAGAAGTTGTGATGCTGCTTATAATGAAAAAAATTCTGGAACATAGATTCGACTTTGTAATAACGTTTAACGGAAATAATTTCGATATCAGGTATATATCTGGAAGGCTAGAAATTCTAGAGAAATCTTTTATATATTTCTCACTTCCTGATGCTACAGAAACAGTTAAACTTAAAATATTTGAAAGATTTGTTACCGGT------------GGAACATTCACTAATAAAACATATCACATAAACAATAATAATGGTGTCATATTTTTTGATTTGTATGCTTTCATACAAAAAACAGAACGATTAGATTCTTACAAACTAGATAGTATATCTAAAAATATATTCAATTGTAACATTACTATAAAAGAAATAGATGACATAGTTTTAACATTGGAAGCTACGGTAAAAGATAATTCTAACGATAAGTTATCTATATTTTCTAGAGTTTTAGAAACCGGTAATTATATCACTATAGGAAATAACGACGTAAGCAAAATAATATATAAAGATATAAACCAAGATAGTTTTATAATTAAAGTTATATCTAATAACAGAGATTATGAAGTAGGGTCATTACATAATATAAGTTTTGGAAAGGATGATGTTGACTTAAAAGATATGTACAAAAACTACAATCTGGAAATAGCGTTAGATATGGAAAAGTATTGTATTCATGATGCGTGTCTCTGTAAATACATATGGGATTATTACAGAGTACCCAGTAAGATTAATGCCGCATCTTCTACTTATCTTTTACCGCAAAGCTTGGCATTAGAATATAGGGCCAGTACTCTTATTAAAGGACCATTACTGAAGTTATTATTGGAAGAACGAGTAATTTATACCAGAAAAATCACAAAGGTAAGATATCCGTATATAGGTGGAAAGGTATTTCTTCCTTCTCAGAAAACTTTCGAAAATAATGTAATGATATTCGATTATAATAGTCTGTATCCAAATGTATGCATCTATGCTAATCTATCACCAGAGAAACTAGTGTGTATAGTGTTAAATACTAATAAACTGGAAGCAGAAATAAATATGAGATCAATCAAAAGTAAGTTCCCATATCCTGATTATGTTTGTATCTCGTGTGAATCTAGACTATCCGATTATTATAGCGAGATTATAGTTTACGATAGAAGAGAGAAAGGTATAATACCTAAGCTTTTAGAGATGTTCATAGGAAAAAGAAAAGAGTATAAAAACCTTTTAAAGACAGCATCGACAACTATAGAAATCACTTTGTACGATTCCTTGCAGTATATCTATAAGATAATAGCAAACTCTGTTTACGGTTTAATGGGATTTAGTAACAGTACGTTATATTCCTATTCTTCCGCAAAGACGTGTACTACTATAGGTAGAAACATGATTACCTATCTAGATTCTATAATGAATGGTGCTGTATGGGAAAACGATAAGCTTATCCTAGCAGATTTTCCTAGAAATATATTTTCTGGAGAAACCATGTTTTCTAAAGAATTAAAAGTAGAACAGATGAATGAAACG---------------------TTTAGATTTCGAAACGTTTACGGAGATACAGATTCTATATTTACAGAAATATCTAATAGAGATGTAGATAAAACCATAAAAATAGCAAAACACCTAGAAAATATAATTAATACAAAAATATTATATGATAACTTTAAAATAGAGTTCGAAGCTGTTTATACACAATTAATTTTACAGTCAAAGAAAAAATACACAACTATAAAGTATTTAGCAAATCATAAGCCAGGTGATAAGACTATAAGAATTAATAAAGGGACTAGTGAAACACGTCGAGACGTAGCATTATTTCATAAACAGATGATACAAAAATATAAAGACTTATTAATGAAAATGTTACTAGAAGGAAAA---GGA---AAAGATATAACTAGATTAATTCTTCAAAGTTTAGAAACAGATATGGTAACCGAGTTT---GCTCATAACAGGGAATTTGATAAATACTTATTAAGTAGGAAACATCACAACAATTATAAATCAGCTACTCACTCAAATTTTGAACTAGTTAAAAGATACAATCTAGAGAATACAGAGAAGATAGAAATAGGAGAAAGATACTTTTATATCTACATATGTGATATTAGTTTACCGTGGCAAAAAAAGCTATGTAATATATTATCCTATGAAGTAATCGCCGATAGTAAATTTTCTCTACCTAAAGACAAAAGAATATTCTATGAAATATATTTTAAAAGAATAGCGTCTGAAGTAGTAAATCTGCTAACGGATAAAACACAATGTACAATATTTTTCAGCAGGCTTTTCGGTACTAAGCCTGTATTTTCATCAGAC

>MT799800.1:116606-119572 Cheloniid poxvirus 1, complete genome

ATGGATATCCGTTGCGTAAACTGGTTCGAAAACAAAAGCGATGTTAAATATATATACCTGAAGGCTCTATGTAAAGATAATAGTGTTATATTTATACGATTTAACTATTACTACTACTACGTGTATAATTCGCAA------GAAGAACTAAGCTTTAAACCTATAGAGAAAATAGAGTTAGGAAATTTTAATATTATAAAGATAGATGAAATTTTAGAAGATAACGTAGAAAAAATTAAAGATAGAGAGTCTTATAAAGCTGATTTAGTTGTAGTAAAAGAT---GTAAAAAGAAGCAGAGAAAGGAAGTATTTAGGAGAATATTTAGATATAACATGGTTTTATATACTTAATAAAATTATGCCAGACGGATGTTATCGTATTGATAAAAACTATTTGACACACGTATATGGAGATTGTTATCACTGTGAAGATGTAAAAAAAGTATTTATAGAAGAAATACCTATTTTCGAAGTTAAGTTCACTTATTTATTCTTTGACATAGAATGTCAGTTTGATAAAAAATTTCCTTCTGTATTTGTTAATCCTATATCTCATATTAGTTGTCTGATAATAGAT---AAAAAAGACGAATATAAGTTTACTCTAATTAATACTGATCTACTACCTGATGTTAAACCTACT---------------------------ATATCATCGCGTAAAGAATTTTCTCCACACGATAGGATTACATTCTGTACCGAAATAGTACTGTTACGCATAATGAAGAAAATACTAGAAGAGAAATTTGATTTTGTAGTTACTTTTAACGGCAATAACTTTGATATACGTTATATTTCGAACAGATTAACTATATTAGAGAAGAAAAGTATAAAATTTTCTCTTCCAGACAATACGGAAACTGTTGATCTAGCCATAATGGGAAGATTTCTTTCAGGA------------GGCAATTTTACTAATAAAACTTATTATATAAACAACAATAACGGGGTTATGTTTTTTGACCTATATACTTTCATTCAGAAAACAGAACGTTTAGATTCATACAAACTAGATAATATATCCAAGAATATATTTAATTGTAATGCTATAATAAAAGATGTAACAAACGATGTAATTACTATGGAAGCTAGCATACGAGAAAATACTAGAGATAAAATATCTATATTTTCTGAAGTATTACAAACTGGTAACTATATCACCATAGGTAATAACGATATAACAAAGATAATATATAAAGATATAAGAGTTGATAGCTTTACTATAAAAGTTATTTGT---AACAAAAAATACGAATGTGGAGGTATACAATTTATAAGCTTTGGTAAAGACGATGTGGATTTAAGGTCTATGTACGCTAATTACAATTTGGATATAGCTTTAGATATGGAAAAATATTGTATGCACGATGCTTGCCTATGTAAATACATATGGGATTATTACAAAATACCTAGTAAAATAGATGCTGCCTCGTCTACATATATGCTACCACAAAGATCGGCTTTAGAATACAGAGCTAGCACTCTTATAAAAGGCCCTCTTCTAAAGCTACTCTTAGAAGAAAAAATAGTATATATTAGAGCGGCCAGCAAAATAAGATATCCTTATATAGGAGGAAAAGTATTTCTTCCTTCACAAAAAACATTTGAAAATAATATTATGGTATTCGATTATAACAGTTTATATCCCAATGTTTGTATTTATGGCAACTTATCCCCAGAAAAATTAGTATGTGTAATATTAAACAGCAATAAACTGGAGTCTGATATAAATCTTAGGATACTAAAGCGCAGGTACTCTTATCCAGATTATATCTGTATCTTATGTGACTCTAGAATAGAGGGATATTACAGCGAGATAATAGTATATGATAGAAGAAGTAAAGGTATTATACCAAAACTTTTAGAACTATTCATATCTAAGAGAAAAAAATACAAATCTCTTCTTAAAGAAGCTGCTACAACAGTAGAAACTACATTGTATGATTCTCTACAATACATATATAAGATAATAGCTAATTCAGTTTATGGATTAATGGGATTCAATAACAGTAGCCTTTACTCTTATTCTTCTGCCAAAACATGTACTACTATAGGGAGAAATATGATAACGTATTTAGATTCTGTCATAAACGGGGCCGTGTGGGAGAACGATAAATTAATACTGGCCGATTTTCCTAGAAATATATTCTCTGGTGAAACTATGTATTCTAAAGAGATAGATGTTTGCTGTATGAATGAAACG---------------------TTTAAATTTAGAAATGTTTATGGTGATACGGATTCCATATTTACAGAAATAACAACTAGAGATGTAGAAAAAACTATTAAAGTAGCAAAAAATTTAGAAGATATTATCAATACAAAAATATTACACGCTAACTTTAAAATAGAGTTTGAAGCTGTATATACACAATTAATTTTGCAATCAAAGAAGAAATACACAACTATAAAGTATCCAGCTAATCACAAACCTGGAGATAAACCTATAAGAATTAACAAAGGAACTAGCGAAACACGTAGAGATGTAGCTTTATTCCATAAACAGATGATACAGAAATACAAGGATCTATTAATGAAAATGCTAATGGAAGGTAAA---AATCAACAAGATATAACTAGAGATATATTACAACATTTGGAAGTTGAAATGGTTACTGAATTC---TCTAATAATAAAGAATTTGAAAAATACTTACTAAGCAGAAAACATCATAACGATTACAAATTAGCAACCCATTCTAACTTTGAATTAGTAAAACAGTATAATTTAGTAAATACAGAAAAGATAGAAATTGGTGAACGTTACTTCTATCTTTATATATGTGATGTAAATATTCCATGGCAGAAAAAACTTTGTAATATTCAATCATACGAAACAATAGCGGATAGTAAATTTTATCTACCTAGAGATAAAAGAATATTTTATGAAGTCTATTTTAAGAGAATAGCATCAGAAGTAGTAAACTTATTAACAGATAAGACTCAGTGTATACTCTTCTTTAATAGGTTATTCGGTACTAGACCTGTATTCTCATCAGAC

>KX857216.1:108448-111414 Shearwaterpox virus strain SWPV-1, complete genome

ATGGATATACGCTGTGTAAATTGGATTGAGAATAAAGGAGATGTTAAATATATATATCTTAAAGCTATAACGAGAGATAACTGTGTTATATTTATACGTTTTAACTATTACTATTATTATGTATATGATTCTGAA------GAAGAACTAAGCTTTACTCCTATAGAAAGGATAGAGTTAGGAAAATTTAACGTTATAAAGATCGATGAAATATTAGAAGATAATATAGAAAATGTTAATGAAAGAGAATCGTATAAATCTGATTTAGTTGTAGTGAAAGAT---GTAAAAAGAAATAGAGATAGAAAGTATCTTCAGGAATATTTAGATATAACATGGTTTTATCTACTTAATAATTTAGTACCTGACGGATGTTATCGTATAAATAGAGACTATTTAAAACACATACATGGTGATTGTTATCATTGTGACGATATAAATAAAGTGTTTGTAGAAGAAATACCTATTTTCGAAGTTAAATTTACTTACCTATTCTTTGATATAGAATGTCAATTCGATAAAAAGTTTCCTTCTGTATTTGTTAATCCTATATCTCATATCAGTTGTCTAATTATAGAT---AAAAAGGAAGAGTATAAATTTACACTGATAAACACAGATCTATTACCCGATGTAAAGCCTACA---------------------------ATAACATCACGTAAAGAATTTTCTCCACATAATAGAATTACATTTTGTAATGAGGTAATTATGTTACGTATAATGAAAAAAATATTGGAAGAAAGATTTGATTTTGTAGTAACATTCAACGGTAATAATTTTGATATACGTTATATTTCTAACAGATTAACTATATTAGAAAAAAAGAGCATAAAATTTTCTCTTCCTGATAATACAGAAACAATAGACTTAGCTATAATGGGAAGATTTCTTTCAGGA------------GGAAATTTTACTAATAAAACATATTATATAAACAACAATAACGGAGTTATGTTCTTTGATCTATACACTTTTATTCAGAAAACAGAACGATTAGATTCTTATAAACTTGATAGCATATCTAAAAATATATTTAATTGTAATGGAATAATAAAAGATATCTCTGAAGGAGTAATTACTATAGAAGCCGATACAAGTAAAAATACAAAGGATAAAATATCTATATTTGCTAAAGTATTACAAACAGGTAATTATATAACTATAGGAAATAATGATGTGTCCAAAATAATATATAAAGATATAAAAACTGACAGTTTTACTATTAAAGTTATTTCC---GACAAAGAATATGAAATTGAAAGCATTCAATCTATAAGTTTTGGAAAAGATGATGTAGATTTAAGATCTATGTACGCCAATTATAATTTAGATATAGCTCTTGACATGGAAAGATATTGTATTCATGATGCCTGTCTATGTAAATATATATGGGACTATTATAAGATACCCAGCAAAATTGATGCAGCTTCGTCTACATATATGTTACCTCAAAGATCGGCTTTGGAATATAGAGCTAGCACGCTTATAAAAGGTCCTCTTCTTAAGCTATTATTAGATGAGAAGATAGTATATGTTAGATCAGCTAGTAAAATAAGATATCCGTATATAGGAGGAAAAGTATTTCTTCCTTCACAAAAGACTTTCGAGAATAACGTCATGGTATTTGATTATAACAGTTTATATCCTAATGTGTGTATTTACGGAAATCTATCTCCAGAAAAATTAGTATGTGTAATATTAAGTAGTAATAAACTAGAATCAGAAATAAATTTTAAAATCTTAAAGCGTAAGTATGCTTATCCAGATTATATATGTGTTTTGTGCGATTCAAGAATAGAAGGTTATTATAGTGAGATTATAGTATACGATAGAAGAAACAAAGGTATAATACCAAAACTATTAGAATTGTTCATAACCAAGAGAAAGAAATATAAATCTCTCCTTAGAGAAGCTACTACAACTGTAGAAACAACACTGTATGACTCATTACAGTATATCTATAAAATTATAGCTAATTCAGTTTATGGATTGATGGGTTTTAATAATAGCAGTCTTTACTCATATTCTTCAGCAAAGACATGTACTACAATAGGTAGAAATATGATAACGTATTTGGATTCTGTTATAAATGGAGCAGTATGGGAGAATGACAAGTTAATATTAGCAGATTTTCCAAGAAATATATTTTCAGGCGAAACTATGTTCTCAAAAGAAATAGAAGTAGAACAAATGAATGAAACT---------------------TTTAAATTTAGAAATGTCTATGGAGATACAGATTCTATATTCACAGAAATATCAAACAGAGACGTTGAAAAAACTATAAAGATAGCAAAAATTCTAGAGAATATAATTAATACAAAGATATTACATGCTAATTTCAAAATAGAGTTTGAAGCGGTTTATACACAATTAATTCTACAATCAAAGAAAAAATACACAACTATTAAATATTTAGCAGATTATAAACCCAGTGATAAACCTATAAGAATTAATAAAGGAACCAGTGAAACACGTAGAGATGTAGCATTATTTCATAAACAGATGATACAAAAGTATAAAGATTTATTAATGAAAATGTTAATGGAAGGAAAA---GGACAACAAGATATAACTAGAGACATATTACAACATTTGGAAGTTGAGATGATAAGCGAATTT---TCAAGTAATAAAGAGTTTGAAAAATATCTTTTGAGTAGGAAACATCATAATGATTATAAATCAGCTACTCATTCCAATTTTGAACTAGTAAAAAATTATAATCTAGTAAACACGGAAAAGATTGAAATAGGAGAAAGATACTTTTATCTTTACATATGTGATATTAATTTACCTTGGCAGAAAAAACTTTGTAACATTCAATCTTATGAAACTATAGCAGATAGCAAATATTCTTTACCTAAAGATAAAAGAATATTTTATGAAGTTTATTTCAAGAGAATAGCATCTGAAGTAGTAAATTTGTTAACAGATAAAACACAATGTATACTTTTCTTCAATAGATTATTCGGTACTAGACCAGTATTCTCATCAGAC

>OM869483.1:115192-118158 Finch poxvirus isolate pox_35627, complete genome

ATGGATATCCGGTGCGTTAATTGGTTTGAAAATAAAGGTGATGTTAAGTATATATATCTAAAGGCTATAACTAAAGATAATGGTGTTATATTTATACGTTTTAATTATTACTATTATTATGTCTATGATTCTGAA------GAAGAGTTAAAATTTAAACCAATAGAAAGAATAGAACTAGGAGAATTTGATATTATAAAGATAGATGAGGTTATAGATAATGATATAGAAACTATTAACGAAAGGAAGACTTATAAATCTAATTTAGTTGTAGTGAAAGAC---GTAAAAAGAAGTAGAGAGAAAAAATACCTAGGAGAATATTTAGATATAACATGGTTTTATATACTAAACAATATTGTACCAGACGGATGCTATCGTATAAATAAAGATCATTTAAAGCAAATATATACCGACTGTTATCATTGCGATAATGTAAGAGAAATGATTATAGAAGAAATACCAATTTTCGAAGTTAAATTTACTTATCTATTCTTTGATATAGAATGTCAGTTTGACAAAAAATTTCCTTCTGTATTTGTTAATCCTATATCCCATATTAGTTGTTTAATAATAGAC---AAAAAAGAAGAATATAAGTTTACTCTGATAAACACAGATCTATTACCAGATGTTGAACCAACG---------------------------ATATCATCGCGTAAACAATTTTGTCCTCGTGATAAAATAACATTTTGTAGTGAAGTAATTATGCTGCGTATAATGAAAAAAATATTAGAAGAAAGATTTGATTTCGTAGTTACGTTTAACGGCAATAACTTTGATATACGTTATATTTCCAATAGACTAACTATATTAGAAAAGAAAAGCATAAAGTTCTCTCTTCCTGATAATACAGAAACTACTGATTTAGCTATAATAGGAAGATTTCTTTCAGGA------------GGTAATTTTACTAATAAAACTTACCATATAAACAACAACAATGGGGTTATGTTCTTCGATTTATATACTTTTATACAGAAAACAGAACGATTGGATTCTTACAAATTAGATAATATATCTAAGAACATATTTAACTGTAACGCTATAATAAAGGATATATCGGATGATATAATTACCATGGAAGCTGGTATAAACGAAAACACTAAAGACAAGATATCTATATTTTCTGAAGTATTACAAACGGGTAATTACATTACTATAGGTAACAGCGATATATCAAAGATAGTATATAAAGATATAAGATCTGATAGTTTTACTATAAAAGTTGTTTCT---AACAAACCATATGAATGCGGAAGTATACAATTCATAAGTTTTGGAAAAGATGATGTAGATTTAAGATCCATGTACGCTAACTATAATCTGGATATAGCACTTGATATGGAAAAATATTGTATTCATGACGCGTGCTTATGTAAATACATATGGGATTATTACAAGATACCTAGTAAAATAGATGCTGCTTCATCTACTTACATGTTACCCCAAAAATCTGCGTTAGAATATAGGGCTAGTACGCTTATCAAAGGTCCTCTACTAAAGTTGTTATTAGAAGAGAAAGTAGTGTATATAAGAGAGGCTAGTAAAGTAAGATACCCGTATATAGGAGGAAAAGTATTTCTCCCATCCCAGAAAACATTCGAAAATAATGTTATGATATTTGACTATAATAGTTTATATCCTAACGTATGTATTTACGCTAATCTATCACCTGAAAAATTAGTATGTATACTATTAAACAGCAATAAACTAGAATCTGATATAAATCTTAAAATGCTAAAGCGAAAGTATCCTTATCCAGATTATGTTTGCGTAATGTGTGATTCTAGAATCGAAGGATATTATAGCGAGATTATAGTATACGATAGAAGGAATAAAGGTATTATACCAAAACTTCTAGAACTGTTTATATCAAAGAGAAAGAAATACAAATCTCTTCTTAAAGAAGCTACTACAACTATTGAAACTACTCTATACGATTCTCTCCAATATATTTACAAGATCATAGCTAATTCAGTTTATGGATTAATGGGATTTCATAGCAGTAGTCTCTATTCCTATTCTTCGGCTAAAACATGTACTACTATAGGCAGAAACATGATAACCTATTTAGATTCTGTTATAAACGGAGCTGTATGGGAAAATGATAAACTAATACTTGCAGATTTTCCTAGAAATATATTTTCTGACAAAACTATGTTTTCGAAAGAAATAGAAGTTCCTCCTATGAATGAAACA---------------------TTTAAATTTAGAAACGTTTACGGAGATACAGATTCTATATTCACAGAGATATCAAATAGAGATGTTGAGAAAACTATAAAGATAGCAAAAATCCTAGAAAATATAATCAATACAAAAATATTACACGCTAATTTCAAAATAGAGTTTGAAGCTGTTTATACACAACTGATTCTTCAGTCAAAGAAAAAATACACTACCATCAAGTATTTAGCAGACTACAAGCCTGGAGATAAACCTATAAGAGTTAATAAAGGAACTAGCGAAACACGTAGAGACGTAGCTCTATTTCATAAACAAATGATACAGAAATATAAAGATCTTTTGATGAAAATGTTAATGGAAGGTAAA---AATCAACAAGATATAACCAGAGATATCTTACAGCATTTAGAAGTTGAAATGGTAAGTGAATTT---TCTAATAATAAAGAATTTGCGAAATATCTTCTTAGTAGAAAACATCATAACGATTACAAGTCTGCCACTCATTCTAATTTTGAATTAGTAAAACAGTATAATCTAATAAACACAGAAAAGATTGAAATCGGTGAACGATACTTTTATCTTTACGTATGCGATATAACGCTTCCGTGGCAGAAAAAACTTTGTAATATTCAGTCATACGAAACAATCGCGGATAGTAAATACTTTTTGCCCAGTGATAAAAGAATATTTTATGAAGTCTATTTTAAGAGAATAGCTTCAGAAGTAGTAAACTTGTTAACAGATAAGACTCAATGTATACTTTTCTTTAACAGATTATTCGGTACTAGGCCGGTATTTTCATCAGAC

>MK903864.1:103203-106169 Magpiepox virus, complete genome

ATGGATATCCGGTGCGTTAATTGGTTTGAGAATAAAGGTGATATTAAGTATATATATCTAAAGGCTATAACTAAAGATAATGGTGTTATATTTATACGTTTTAACTATTACTATTATTATGTCTATGATTCTGAA------GAAGAGTTAAAATTTAAACCAATAGAGAGAATAGAACTAGGAAAATTTGATATTATAAAGATAGATGAGGTTTTAAATGACGATATAGAAAATATTAACGAAAGAAAGACTTATAAATCTGATTTAGTTGTAGTGAAAGAC---GTAAAAAGAAGTAGAGAAAAAAAGTATTTAGGAGAATATTTAGATATAACATGGTTTTATATACTAAACAATATTGTACCAGATGGATGTTATCGCATAAACAAAGATAATTTAAAACAAATATATACTGACTGCTATCATTGCGATAATGTGAAAGAAATGATTATAGAGGAAATACCAATTTTCGAAGTTAAATTTACTTACCTATTCTTCGATATAGAATGTCAGTTTGATAAAAAATTTCCTTCTGTATTTGTTAATCCTATATCCCATATTAGTTGTTTAATAATAGAC---AAAAAAGATGAGTATAAATTTACTCTGATAAACACAGATTTATTACCAGATGTTGAACCTACT---------------------------ATATCATCGCGCAAACAATTTTGTCCTCGTGATAAAATAACATTTTGTAGCGAAGTAATTATGCTACATATAATGAAAAAAATACTTGAAGAGAGATTTGATTTCGTAGTCACGTTTAACGGCAATAACTTTGATATACGTTATATTTCTAACAGACTAACTATATTAGAAAAGAAAAGCATAAAGTTCTCTCTTCCTGATAACACAGAAACTACTGATTTAGCTATAATAGGAAGATTTCTTTCAGGA------------GGTAATTTTACTAATAAAACTTACCATATAAACAACAACAATGGGGTTATGTTCTTCGATTTATATACTTTTATACAGAAAACAGAACGATTGGATTCTTACAAATTAGATAATATATCTAAGAACATATTTAACTGTAACGCTATAATAAAGGATATATCGGATGATATAATTACAATGGAAGCTGGTATAAACGAAAATACCAAAGACAAAATATCTATATTTTCTGAAGTATTACAAACTGGTAATTATATTACTATAGGTAACAGCGATATATCAAAAATAGTATATAAAGATATAAGATCTGATAGTTTTACTATAAAAGTTGTTTCT---AACAAACCATATGAATGCGGAAGTATACAATTCATAAGTTTTGGAAAAGATGATGTAGATTTAAGATACATGTACGCTAACTATAATCTGGATATAGCACTTGATATGGAAAAATATTGTATTCATGACGCGTGCTTATGTAAATACATATGGGATTATTACAAGATACCTAGTAAAATAGATGCTGCTTCATCTACTTACATGTTACCCCAAAAATCTGCGTTAGAATATAGGGCTAGTACGCTTATCAAAGGTCCTCTCCTAAAGTTGTTATTAGAAGAGAAAGTAGTGTATATAAGAGAGGCTAGTAAAGTAAGATACCCGTATATAGGAGGGAAAGTATTTCTCCCATCCCAGAAAACATTTGAAAATAATGTTATGATATTTGATTATAATAGTTTATATCCTAATGTATGTATTTACGCTAACCTATCACCTGAAAAATTAGTATGTATACTATTAAACAGCAATAAACTAGAATCTGAGATAAATCTTAAAATGCTAAAGCGCAAGTATCCTTACCCAGATTATGTTTGTGTAATGTGTGATTCTAGAATAGAAGGATATTATAGCGAGATTATAGTATACGATAGAAGGAATAAAGGTATTATACCAAAACTTCTAGAATTATTTATATCAAAGAGAAAGAAATACAAATCTCTTCTTAAAGAAGCTACTACAACTATTGAAACTACTCTATACGATTCCCTCCAATATATTTACAAGATCATAGCTAATTCAGTTTACGGATTAATGGGATTTCATAGCAGTAGTCTCTATTCCTATTCTTCGGCTAAAACATGTACTACTATAGGCAGAAACATGATAACCTATTTAGATTCTGTTATAAACGGAGCTGTATGGGAAAATGATAAACTAATACTTGCAGATTTTCCTAGGAATATATTTTCTGACAAAACTATGTTTTCTAAAGAAATAGAAGTTCCTCCTATGAACGAAACA---------------------TTTAAATTTAGAAACGTTTACGGAGATACAGATTCTATATTCACAGAGATATCAAATAGAGATGTTGAGAAAACTATAAAGATAGCAAAAATCCTGGAAAATATAATCAATACAAAAATATTACACGCTAATTTCAAAATAGAGTTTGAAGCTGTTTATACGCAACTGATTCTTCAGTCAAAGAAAAAATATACTACCATAAAGTATTTAGCAGACTACAAGCCAGGAGATAAACCTATAAGAGTTAACAAAGGAACTAGCGAAACACGTAGAGACGTAGCTCTATTTCATAAACAAATGATACAGAAATATAAAGATATTTTGATGAAAATGTTAATGGAAGGTAAA---AATCAACAAGATATAACCAGAGATATTTTACAGCATTTAGAAGTTGAAATGGTAAGTGAATTT---TCTAATAATAAAGAATTTGTGAAATATCTTCTTAGTAGAAAACATCATAACGATTACAAGTCGGTAACTCATTCTAATTTTGAATTAGTAAAACAGTATAATCTAATAAACACAGAAAAGATTGAAATCGGTGAACGATACTTTTATCTTTACGTATGCGATATAACGCTTCCGTGGCAGAAAAAACTTTGTAATATTCAATCATACGAAACAATCGCGGATAGTAAATACTTTTTACCTAGTGATAAAAGAATATTTTATGAAGTCTATTTTAAGAGAATAGCTTCAGAAGTGGTAAACCTATTAACGGATAAGACTCAATGTATACTTTTCTTTAATAGATTATTCGGTACTAAACCGGTATTTTCATCAGAC

>NC_005309.1:122909-125875 Canarypox virus, complete genome

ATGGATATCCGGTGCGTTAATTGGTTTGAGAATAAAGGTGATATTAAGTATATATATCTAAAGGCTATAACTAAAGATAATGGTGTTATATTTATACGTTTTAACTATTACTATTATTATGTCTATGATTCTGAA------GAAGAGTTAAAATTTAAACCAATAGAGAGAATAGAACTAGGAGAATTTGATATTATAAAGATAGATGAGGTTTTAGATGACGATATAGAAAATATTAACGAAAGGAAGACTTATAAATCTGATTTAGTTGTAGTGAAAGAC---GTAAAAAGAAGTAGAGAAAAAAAGTATTTAGGAGAATATTTAGATATAACATGGTTTTATATACTAAACAATATTGTACCAGATGGATGTTATCGCATAAACAAAGATAATTTAAAACAAATATATACCGACTGCTATCATTGCGATAATGTGAAAGAAATGATTATAGAGGAAATACCAATTTTCGAAGTTAAATTTACTTATCTATTCTTTGATATAGAATGTCAGTTTGACAAAAAATTTCCTTCTGTATTTGTTAATCCCATATCTCATATTAGTTGTTTAATAATAGAC---AAAAAAGATGAGTATAAGTTTACTCTGATAAACACAGATTTATTACCAGATGTTGAGCCTACT---------------------------ATATCATCGCGCAAACAATTTTGTCCTCGCGATAAAATAACATTTTGTAGCGAAGTAATTATGCTACGTATAATGAAAAAAATACTTGAAGAGAGATTTGATTTCGTAGTTACGTTTAACGGTAATAACTTTGATATACGTTATATTTCCAATAGACTAACTATATTAGAAAAGAAAAGCATAAAGTTCTCTCTTCCTGATAACACAGAAACTACTGATTTAGCTATAATAGGAAGATTTCTTTCAGGA------------GGTAATTTTACTAATAAAACTTACCATATAAACAACAACAATGGGGTTATGTTCTTCGATTTATATACTTTTATACAGAAAACAGAACGATTGGATTCTTACAAATTAGATAATATATCTAAGAACATATTTAACTGTAACGCTATAATAAAGGATATATCGGATGATATAATTACCATGGAAGCTGGTATAAACGAAAATACCAAAGACAAGATATCTATATTTTCTGAAGTATTACAAACGGGTAATTACATTACTATAGGTAACAGCGATATATCAAAGATAGTATATAAAGATATAAGATCTGATAGTTTTACTATAAAAGTTGTTTCT---AACAAACCATATGAATGCGGAAGTATACAATTCATAAGTTTCGGAAAAGATGATGTAGATTTAAGATCCATGTACGCTAACTATAATCTGGATATAGCACTTGATATGGAAAAATATTGTATTCATGACGCGTGCTTATGTAAATACATATGGGATTATTACAAGATACCTAGTAAAATAGATGCTGCTTCATCTACTTACATGTTACCCCAAAAATCTGCGTTAGAATATAGGGCTAGTACGCTTATCAAAGGTCCTCTCCTAAAGTTGTTATTAGAAGAGAAAGTAGTGTATATAAGAGAGGCTAGTAAAGTAAGATACCCATATATAGGAGGAAAAGTATTTCTTCCATCCCAGAAAACATTCGAAAATAATGTTATGATATTTGATTATAATAGTTTATATCCTAACGTATGTATTTACGCTAACCTATCACCTGAAAAATTAGTATGTATACTATTAAACAGCAATAAACTAGAATCTGATATAAATCTTAAAATGCTAAAGCGAAAGTATCCTTATCCAGATTATGTTTGCGTAATGTGTGATTCTAGAATCGAAGGATATTATAGCGAGATTATAGTATACGATAGAAGGAATAAAGGTATTATACCAAAACTTCTAGAATTGTTTATATCAAAGAGAAAGAAATACAAATCTCTTCTTAAAGAAGCTACTACAACTATTGAAACTACTCTATACGATTCTCTCCAATATATTTACAAGATCATAGCTAATTCAGTTTATGGATTAATGGGATTTCATAGCAGTAGTCTCTATTCCTATTCTTCTGCTAAAACATGTACTACTATAGGCAGAAACATGATAACCTATTTAGATTCTGTTATAAACGGAGCTGTATGGGAAAATGATAAACTAATACTTGCAGATTTTCCTAGGAATATATTTTCTGATAAAACTATGTTTTCTAAAGAAATAGAAGTTCCTCCTATGAACGAAACA---------------------TTTAAATTTAGAAACGTTTACGGAGATACAGATTCTATATTCACAGAGATATCAAATAGAGATGTTGAGAAAACTATAAAGATAGCAAAAATCCTGGAAAATATAATCAATACAAAGATATTACACGCTAATTTCAAAATAGAGTTTGAAGCTGTTTATACGCAACTGATTCTTCAGTCAAAGAAAAAATATACTACTATCAAGTATTTAGCAGACTACAAGCCAGGAGATAAACCTATAAGAGTTAACAAAGGAACTAGCGAAACACGTAGAGACGTAGCTCTATTTCATAAACAAATGATACAGAAATATAAAGATCTTTTGATGAAAATGTTAATGGAAGGTAAA---AATCAACAAGATATAACCAGAGATATTTTACAGCATTTAGAAGTTGAAATGGTAAGTGAATTT---TCTAATAATAAAGAATTTACGAAATATCTTCTTAGTAGAAAACATCATAACGATTACAAGTCTGCCACTCATTCTAATTTTGAATTAGTAAAACAGTATAATCTAATAAATACAGAAAAGATTGAAATCGGTGAACGATACTTTTATCTTTACGTATGCGATATAACGCTTCCGTGGCAGAAAAAACTTTGTAATATTCAGTCATACGAAACAATCGCAGATAGTAAATACTTTTTGCCCAGTGATAAAAGAATATTTTATGAAGTCTATTTTAAGAGAATAGCTTCAGAAGTAGTAAACTTGTTAACAGATAAGACTCAATGTATACTTTTCTTTAACAGATTATTCGGTACTAGGCCGGTATTTTCATCAGAC

>ON408417.1:116303-119269 Crowpox virus strain 122740AU/2021, complete genome

ATGGATATCCGGTGCGTTAATTGGTTTGAGAATAAAGGTGATATTAAGTATATATATCTAAAGGCTATAACTAAAGATAATGGTGTTATATTTATACGTTTTAACTATTACTATTATTATGTCTATGATTCTGAA------GAGGAGTTAAAATTTAAACCAATAGAGAGGATAGAACTAGGAGAATTTGATATTATAAAGATAGATGAGGTTTTAGATGACGATATAGAAAATATTAACGAAAGGAAGACTTATAAATCTGATTTAGTTGTAGTGAAAGAC---GTAAAAAGAAGTAGAGAGAAAAAGTATTTAGGAGAATATTTAGATATAACATGGTTTTATATACTAAACAATATTGTACCAGATGGATGTTATCGCATAAACAGAGATAATTTAAAACAAATATATACTGACTGCTATCATTGCGATAATGTGAAAGAAATGATTATAGAGGAAATACCAATTTTCGAAGTTAAATTTACTTACCTATTCTTCGATATAGAATGTCAGTTTGATAAAAAATTTCCTTCTGTATTTGTTAATCCCATATCTCATATTAGTTGTTTAATAATAGAC---AAAAAAGATGAGTATAAGTTTACTCTGATAAACACAGATTTATTACCAGATGTTGAACCTACT---------------------------ATATCATCGCGCAAACAATTTTGTCCTCGCGATAAAATAACATTTTGTAGCGAAGTAATTATGCTACGTATAATGAAAAAAATACTTGAAGAGAGATTTGATTTCGTAGTCACGTTTAACGGCAATAACTTTGATATACGTTATATTTCTAACAGACTGACTATATTAGAAAAGAAAAGCATAAAGTTCTCTCTTCCTGATAACACAGAAACTACTGATTTAGCTATAATAGGAAGATTTCTTTCAGGA------------GGTAATTTTACTAATAAAACTTACCATATAAACAACAACAATGGGGTTATGTTCTTCGATTTATATACTTTTATACAGAAAACAGAACGATTGGATTCTTACAAATTAGATAATATATCTAAGAACATATTTAACTGTAACGCTATAATAAAGGATATATCGGATGATATAATTACCATGGAAGCTGGTATAAACGAAAATACCAAAGACAAAATATCTATATTTTCTGAAGTATTACAAACTGGTAATTACATTACTATAGGTAACAGCGATATATCAAAGATAGTATATAAAGATATAAGATCTGATAGTTTTACTGTAAAAGTTGTTTCT---AACAAACCATATGAATGCGGAAGTATACAATTCATAAGTTTTGGAAAAGATGATGTAGATTTAAGATCCATGTACGCTAACTATAATCTGGATATAGCACTTGATATGGAAAAATATTGTATTCATGACGCGTGCTTATGTAAATACATATGGGATTATTACAAGATACCTAGTAAAATAGATGCTGCTTCATCTACTTACATGTTACCCCAAAAATCTGCGTTAGAATATAGGGCTAGTACGCTTATCAAAGGTCCTCTCCTAAAGTTGTTATTAGAAGAGAAAGTAGTGTATATAAGAGAGGCTAGTAAAGTAAGATACCCGTATATAGGAGGGAAAGTATTTCTCCCATCCCAGAAAACATTCGAAAATAATGTTATGATATTTGATTATAATAGTTTATATCCTAACGTATGTATTTACGCTAACTTATCACCTGAAAAATTAGTATGTATACTATTAAACAGCAATAAACTAGAATCTGAGATAAATCTTAAAATGCTAAAGCGAAAGTATCCTTACCCAGATTATGTTTGCGTAATGTGTGATTCTAGAATCGAAGGATATTATAGCGAGATTATAGTATACGATAGAAGGAATAAAGGTATTATACCAAAACTTCTAGAATTGTTTATATCAAAGAGAAAGAAATACAAATTTCTTCTTAAGGAAGCTACTACAACTATTGAAACTACTCTATACGATTCTCTACAATATATTTACAAGATCATAGCTAATTCAGTTTATGGATTAATGGGATTTCATAGCAGTAGTCTCTATTCGTATTCTTCGGCTAAAACATGTACTACTATAGGCAGAAACATGATAACCTATTTAGATTCTGTTATAAACGGAGCTGTATGGGAAAATGATAAACTAATACTTGCAGATTTTCCTAGAAATATATTTTCTGACAAAACCATGTTTTCTAAAGAAATAGAAGTTCCTCCTATGAACGAAACA---------------------TTTAAATTTAGAAACGTTTACGGAGATACAGATTCTATATTCACAGAGATATCAAATAGAGATGTTGATAAAACTATAAAGATAGCAAAAATCCTGGAAAATATAATCAATACAAAAATATTACACGCTAATTTCAAAATAGAGTTTGAAGCTGTTTATACGCAACTGATTCTTCAGTCAAAGAAAAAATACACTACCATCAAGTATTTAGCAGACTACAAGCCAGGAGATAAACCTATAAGAGTTAACAAGGGAACCAGCGAAACTCGTAGAGACGTAGCTTTATTTCATAAGCAAATGATACAGAAATATAAAGATCTTTTGATGAAAATGTTAATGGAAGGTAAA---AATCAACAAGATATAACCAGAGATATTTTACAGCATTTAGAAGTTGAAATGGTAAGTGAATTT---TCTAATAATAAAGAATTTGCGAAATATCTTCTTAGTAGAAAACATCACAACGATTACAAGTCTGCCACTCATTCTAATTTTGAATTAGTAAAACAGTATAATCTAATAAACACAGAAAAGATTGAAATCGGTGAACGATACTTTTATCTTTACGTATGCGATATAACGCTTCCGTGGCAGAAAAAACTTTGTAATATTCAGTCATACGAAACAATCGCAGATAGTAAATACTTTTTACCCAGTGATAAAAGAATATTTTATGAAGTCTATTTTAAGAGAATAGCTTCAGAAGTAGTAAACTTGTTAACAGATAAGACGCAATGTATACTTTTCTTTAACAGATTATTCGGTACTAGGCCGGTATTTTCATCAGAC

>NC_006998.1:c56656-53636 Vaccinia virus, complete genome

ATGGATGTTCGGTGCATTAATTGGTTTGAAAGTCACGGTGAAAACAGATTTTTATATCTGAAATCCAGATGTCGAAATGGTGAGACCGTATTTATACGATTTCCTCATTACTTTTATTACGTAGTGACGGACGAAATATATCAGTCATTGTCTCCTCCTCCATTTAATGCGAGGCCGTTGGGAAAGATGAGAACTATAGACATTGACGAGACAATAAGTTATAATCTA---GATATTAAAGATAGAAAATGCTCCGTCGCAGATATGTGGTTGATAGAAGAGCCAAAGAAACGCAGCATACAAAATGCCACCATGGATGAATTTCTCAATATTAGTTGGTTTTATATTTCTAACGGGATATCTCCAGACGGATGTTACTCGTTGGACGAGCAATATTTGACAAAGATTAACAATGGATGTTATCATTGTGACGATCCACGTAACTGTTTCGCTAAAAAAATACCTAGATTCGATATCCCAAGATCGTACTTATTTCTAGATATAGAGTGTCACTTCGATAAGAAGTTTCCTTCTGTATTTATTAACCCAATCTCGCATACAAGTTACTGTTATATCGATTTAAGTGGTAAACGATTATTGTTTACGCTCATTAATGAAGAGATGTTAACGGAACAGGAAATACAAGAAGCCGTCGATAGAGGATGTTTGAGGATACAGTCACTAATGGAAATGGATTACGAACGAGAACTAGTTTTATGTTCTGAAATAGTTTTGTTACGAATAGCTAAACAATTGTTGGAACTAACGTTCGACTATGTCGTTACCTTTAACGGACATAACTTTGATCTGAGATATATTACTAATCGTCTAGAGTTATTAACAGGAGAGAAGATTATCTTTAGATCTCCGGACAAAAAGGAAGCTGTACATCTCTGTATTTATGAGAGAAATCAGTCTAGTCATAAGGGAGTAGGCGGCATGGCCAATACTACGTTTCACGTTAATAACAATAATGGAACTATATTTTTCGATCTATATTCATTCATTCAAAAATCTGAAAAATTGGATTCGTACAAATTGGATTCTATATCCAAGAACGCGTTCAGTTGCATGGGTAAAGTATTAAATAGAGGAGTTAGAGAAATGACGTTCATCGGTGACGATACTACGGACGCGAAAGGCAAAGCCGCTGCATTTGCAAAGGTTTTAACCACAGGTAATTATGTGACTGTTGATGAGGATATTATATGTAAAGTAATTCGTAAAGATATTTGGGAAAATGGATTTAAAGTCGTACTATTATGT---------CCTACTTTACCTAATGATACATATAAATTATCTTTCGGAAAGGATGACGTTGATTTAGCTCAGATGTATAAGGATTATAATCTAAACATAGCTTTAGATATGGCTAGATACTGTATTCATGATGCTTGTTTGTGTCAGTATTTGTGGGAGTATTATGGAGTAGAAACAAAAACAGACGCGGGTGCGTCAACATATGTGCTTCCTCAATCCATGGTATTCGAATATAGAGCGAGTACAGTCATCAAGGGTCCACTGTTAAAGCTATTGTTGGAAACTAAAACTATCTTAGTTAGATCAGAAACAAAACAAAAGTTTCCTTATGAAGGCGGTAAGGTATTTGCTCCAAAACAAAAAATGTTTAGTAATAATGTATTAATCTTTGATTATAACAGTCTGTATCCTAATGTGTGTATCTTTGGAAATCTATCTCCGGAAACATTAGTCGGTGTCGTTGTTAGTACCAATAGATTGGAAGAAGAAATAAATAATCAGCTCTTGCTTCAGAAATATCCACCTCCTAGATATATTACGGTTCATTGTGAACCTAGACTACCGAACCTCATCTCTGAAATAGCAATTTTCGATAGATCGATAGAAGGAACCATTCCTAGACTATTAAGAACATTTTTGGCAGAGAGAGCCAGATATAAAAAGATGTTAAAACAGGCTACCAGTTCAACTGAAAAGGCCATCTATGATTCCATGCAATATACGTACAAGATAGTAGCCAACTCAGTATATGGTCTGATGGGATTTAGAAATAGTGCTCTATACTCATACGCTTCGGCTAAGAGTTGCACATCCATAGGACGTAGAATGATCTTGTATCTAGAATCGGTACTAAATGGAGCAGAGTTATCTAACGGTATGTTACGGTTTGCCAATCCATTAAGTAATCCATTT---------------TATATGGACGATAGAGATATTAATCCGATTGTGAAAACATCGTTGCCTATAGATTACAGATTTCGTTTTCGTAGCGTGTATGGAGATACCGACTCCGTGTTTACAGAGATAGACAGTCAAGATGTAGATAAGTCCATAGAAATAGCAAAGGAGTTAGAACGACTGATTAATAATAGAGTATTGTTTAATAATTTTAAAATAGAGTTTGAGGCGGTATATAAGAATCTGATTATGCAATCGAAGAAGAAATATACAACGATGAAATACTCGGCATCGTCGAATTCAAAATCTGTACCTGAGAGAATTAATAAAGGTACTAGTGAAACTAGAAGAGATGTTTCCAAGTTTCATAAGAATATGATTAAGACATACAAGACCAGACTGTCTGAGATGTTGTCTGAAGGACGGATGAATTCTAATCAGGTATGTATAGATATTCTCCGTTCTTTAGAAACAGATTTACGATCCGAATTTGATAGTAGATCGTCTCCTCTAGAATTATTTATGTTGAGTCGAATGCATCACTCAAATTATAAATCCGCAGATAACCCTAATATGTATTTGGTTACTGAATATAATAAAAATAATCCAGAAACTATAGAACTTGGAGAACGATATTATTTTGCATATATTTGTCCGGCTAATGTACCATGGACCAAAAAACTTGTAAATATTAAAACATATGAAACAATTATCGATAGAAGTTTTAAACTCGGCAGTGATCAAAGAATATTTTACGAAGTTTACTTTAAACGATTGACGTCCGAAATAGTCAATCTATTGGATAATAAAGTTTTATGCATCTCATTCTTTGAAAGAATGTTTGGTTCAAAACCTACATTTTACGAAGCA

GSDM Tree

(MF001304.1_c165763-165224_Murmansk_poxvirus_strain_LEIV-11411_complete_genome:0.0778910538,MF001305.1_c164808-164269_NY_014_poxvirus_strain_2013_complete_genome:0.0774941485,((KU749311.1_173861-174445_Volepox_virus_strain_CA_complete_genome:0.0772347044,(KU749309.1_150174-150722_Raccoonpox_virus_strain_85A_complete_genome:0.0897280289,KU749310.1_172437-172985_Skunkpox_virus_strain_WA_complete_genome:0.0773348965)98:0.0131910516)99:0.8653195281,(((NP_051846.1_m132L_Myxoma_virus:0.1007421399,NP_052021.1_gp132L_Rabbit_fibroma_virus:0.2444396356)100:1.7069206684,(((WCB86927.1_CPPV116_hypothetical_protein__Cooks_petrelpox_virus_:1.2147511728,(((ARF02807.1_SWPV1-246__Shearwaterpox_virus_:1.3432104682,((WCB86827.1_CPPV016_hypothetical_protein__Cooks_petrelpox_virus_:0.0000021277,YP_009448176.1_hypothetical_protein_C1178_gp283__Flamingopox_virus_FGPVKD09_:0.0304161583)100:0.2704272335,(YP_009046354.1_hypothetical_protein_HM89_gp126__Pigeonpox_virus_:0.0836667227,(WCB86986.1_CPPV175_hypothetical_protein__Cooks_petrelpox_virus_:0.0556853009,YP_009448040.1_hypothetical_protein_C1178_gp125__Flamingopox_virus_FGPVKD09_:0.0917737271)100:0.1531745483)100:0.5693510950)73:0.0969293647)95:0.3869405993,(UOX38602.1_hypothetical_protein__Finch_poxvirus_:0.1342098153,NP_955035.1_hypothetical_protein_CNPV012__Canarypox_virus_:0.1784389795)100:0.4984614376)94:0.1767606731,((ARF02766.1_SWPV1-192__Shearwaterpox_virus_:0.7898722232,YP_009177139.1_hypothetical_protein_ASN15_gp118__Turkeypox_virus_:1.1626011997)93:0.1963170823,QRY18970.1_ORF-100__Teiidae_poxvirus_1_:1.0963097821)66:0.1816702298)62:0.1770755974)65:0.3315729645,((((((((URH27575.1_hypothetical_protein_99866_00235__Fowlpox_virus_:0.0169224256,ART91662.1_hypothetical_protein__Fowlpox_virus_:0.0000029581)99:0.0055670710,(URH28352.1_hypothetical_protein_V_cmp_00231__Fowlpox_virus_:0.0066921228,UNS14472.1_ALPV-308__Albatrosspox_virus_:0.0000029581)99:0.0066935891)75:0.0000024287,AYO89822.1_hypothetical_protein_FPV229__Fowlpox_virus_:0.0053503223)59:0.0000021114,NP_039192.1_hypothetical_protein_FPV229__Fowlpox_virus_:0.0000029581)70:0.0000022362,AXY04933.1_hypothetical_protein__Fowlpox_virus_:0.0000029581)100:0.1870925695,(YP_009046452.1_putative_A47L-like_protein__Pigeonpox_virus_:0.0156319452,(YP_009448152.1_putative_A47L-like_protein__Flamingopox_virus_FGPVKD09_:0.0052023978,WCB87127.1_CPPV316_putative_A47L-like_protein__Cooks_petrelpox_virus_:0.0000029581)97:0.0053190682)90:0.0220827866)95:0.0478498644,YP_009046221.1_putative_A47L-like_protein__Penguinpox_virus_:0.0000027381)99:0.4933524879,(ARF02840.1_SWPV1-281__Shearwaterpox_virus_:0.4071458082,(AYP74072.1_hypothetical_protein__Fowlpox_virus_:0.3162196939,(QRI43019.1_hypothetical_protein_ChPV301__Cheloniid_poxvirus_1_:0.0690597986,(QGM48949.1_conserved_hypothetical_protein__Magpiepox_virus_:0.0217939703,(UOX38782.1_hypothetical_protein__Finch_poxvirus_:0.1551379011,NP_955325.1_hypothetical_protein_CNPV302__Canarypox_virus_:0.1386113011)100:0.1608940337)100:0.1948511529)77:0.1421976118)99:0.1321900066)100:0.4301825649)97:0.4371865842)55:0.3236139194,(((AXY05184.1_hypothetical_protein__Fowlpox_virus_:0.0000029581,((NP_039184.1_hypothetical_protein_FPV221__Fowlpox_virus_:0.0052969467,AYO89814.1_hypothetical_protein_FPV221__Fowlpox_virus_:0.0000029581)98:0.0053009375,ART91654.1_vaccinia_A47L-like_protein__Fowlpox_virus_:0.0000029581)97:0.0106360807)93:0.0000022676,AXY04662.1_hypothetical_protein__Fowlpox_virus_:0.0000029581)93:0.0234476736,(YP_009046447.1_A47L-like_protein__Pigeonpox_virus_:0.0327612093,(YP_009448146.1_hypothetical_protein_C1178_gp240__Flamingopox_virus_FGPVKD09_:0.0000029581,YP_009046216.1_A47L-like_protein__Penguinpox_virus_:0.0214946013)73:0.0053316400)62:0.0541759175)99:0.8348700331)100:1.4987768715)54:0.3377794906,((YP_009408097.1_Immunoprevalent_protein_Eptesipox_virus:0.8448924259,(((MN240300.1_c166555-165827_Alaskapox_virus_complete_genome:0.0882576316,(((((AY009089.1_c154402-154034_Camelpox_virus_CMS_complete_genome:0.0000021886,MK910851.1_c154204-153836_Camelpox_virus_strain_Negev2016_complete_genome:0.0081178059)100:0.1459253102,DQ437594.1_c153307-152573_Taterapox_virus_strain_Dahomey_1968_complete_genome:0.0119958306)95:0.0000029581,(KY358055.1_c145385-144651_Variola_virus_strain_VD21_17th_century_complete_genome:0.0000029581,(DQ441432.1_c146026-145292_Variola_virus_strain_Korea_1947__Lee_Masterseed__complete_genome:0.0039844627,DQ441437.1_c146904-146170_Variola_virus_strain_Sierra_Leone_1969__V68-258__complete_genome:0.0000029581)100:0.0039866094)100:0.0079950601)97:0.0123029440,MH607143.1_c167386-166652_Akhmeta_virus_isolate_Vani_2010_complete_genome:0.0241476595)76:0.0000024722,((MN912466.1_c156293-155559_Ectromelia_virus_WH_complete_genome:0.0200355295,(KC813500.1_c153312-152578_Cowpox_virus_strain_MonKre08_4_complete_genome:0.0000029581,((AY243312.1_c154675-153917_Vaccinia_virus_WR_complete_genome:0.0038712163,MG599038.1_c157906-157148_Buffalopox_virus_isolate_Karachi_2005_complete_genome:0.0000029581)100:0.0040107937,(KY349117.1_c166272-165538_Horsepox_virus_strain_MNR_complete_genome:0.0039926603,(M35027.1_c153690-152956_Vaccinia_virus_Copenhagen_complete_genome:0.0039916634,AY484669.1_c159516-158782_Rabbitpox_virus_complete_genome:0.0000029581)100:0.0000029581)99:0.0039875890)100:0.0120342452)98:0.0000027251)100:0.0040453616,NC_055231.1_c163765-163031_Orthopoxvirus_Abatino_complete_genome:0.0079198669)96:0.0118913722)83:0.0259624901)99:0.1233778634,(KU749309.1_c145369-144644_Raccoonpox_virus_strain_85A_complete_genome:0.1099870192,(KU749310.1_c167631-166873_Skunkpox_virus_strain_WA_complete_genome:0.0434865176,KU749311.1_c169057-168281_Volepox_virus_strain_CA_complete_genome:0.0831491866)84:0.0129269240)78:0.0891389337)91:0.3746874561,(HQ849551.1_c134784-134035_Yoka_poxvirus_strain_DakArB_4268_complete_genome:0.3723503913,(MF001305.1_c159640-158771_NY_014_poxvirus_strain_2013_complete_genome:0.0871240290,MF001304.1_c160603-159728_Murmansk_poxvirus_strain_LEIV-11411_complete_genome:0.1244014181)100:0.1715678788)97:0.1819946196)94:0.9642791798)62:0.1219734083,(((((((((((((ref_XP_032903802.1__location_chromosome_chromosome_2_completeness_complete_organism_Amblyraja_radiata_isolate_CabotCenter1_gcode_1_chromosome_2_sex_male_tissue_type_testis_liver_country_USA__Gulf_ofa:0.9848847129,(ref_XP_031796450.1__location_chromosome_chromosome_5_completeness_complete_organism_Sarcophilus_harrisii_gcode_1_chromosome_5_cell_line_91H_gasdermin-E_Sarcophilus_harrisii:0.6028996068,((((ref_NP_061239.1__chromosome_6_organism_Mus_musculus_strain_C57BL_6_gcode_1_chromosome_6_map_6_gasdermin-E_precursor_Mus_musculus:0.0169116605,ref_XP_021046698.1__location_chromosome_chromosome_2_completeness_complete_organism_Mus_pahari_gcode_1_chromosome_2_sex_female_tissue_type_tail_gasdermin-E_Mus_pahari:0.0446093379)100:0.2616328423,ref_XP_004626528.1__chromosome_Unknown_completeness_complete_organism_Octodon_degus_isolate_3935_gcode_1_chromosome_Unknown_sex_female_gasdermin-E_Octodon_degus:0.1205800210)100:0.0789339559,(ref_NP_001180041.1__chromosome_4_organism_Bos_taurus_breed_Hereford_gcode_1_chromosome_4_map_4_gasdermin-E_Bos_taurus:0.1525732072,(ref_XP_024418892.1__chromosome_Unknown_completeness_complete_organism_Desmodus_rotundus_isolate_DRU21DN04_gcode_1_chromosome_Unknown_sex_male_tissue_type_muscle_skin_sample_dev_stage_adult_country_Us:0.1245707096,(ref_XP_006912063.1__chromosome_Unknown_completeness_complete_organism_Pteropus_alecto_gcode_1_chromosome_Unknown_sex_male_tissue_type_kidney_country_Australia__Anglican_Church_Grammar_School_East_Bro:0.0421630138,ref_XP_016015046.2__chromosome_Unknown_completeness_complete_organism_Rousettus_aegyptiacus_isolate_mRouAeg1_gcode_1_chromosome_Unknown_sex_male_tissue_type_muscle_dev_stage_adult_country_USA__Berkes:0.0713843142)100:0.1090459579)100:0.0810243640)99:0.0663626247)92:0.0520691002,(ref_XP_001096213.2__location_chromosome_chromosome_3_completeness_complete_organism_Macaca_mulatta_isolate_AG07107_bio_material_Coriell_AG07107_gcode_1_chromosome_3_sex_female_tissue_type_fibroblasta:0.0624636669,(ref_NP_001120925.1__chromosome_7_organism_Homo_sapiens_gcode_1_chromosome_7_map_7p15.3_gasdermin-E_isoform_a_Homo_sapiens:0.0045183791,ref_XP_001159685.3__location_chromosome_chromosome_7_completeness_complete_organism_Pan_troglodytes_isolate_Yerkes_chimp_pedigree__C0471__Clint__gcode_1_chromosome_7_sex_male_tissue_type_blood_dev_ss:0.0091344002)100:0.0032830680)100:0.6247006594)100:0.2578624534)100:0.3084567364)58:0.2782988578,ref_XP_027689547.1__location_chromosome_chromosome_2_completeness_complete_organism_Chelonia_mydas_isolate_rCheMyd1_gcode_1_chromosome_2_sex_male_tissue_type_blood_dev_stage_adult_country_Israel__Mes:0.0943396295)53:0.1633278925,ref_XP_023962559.1__location_chromosome_chromosome_2_completeness_complete_organism_Chrysemys_picta_bellii_isolate_RCT428_sub_species_bellii_gcode_1_chromosome_2_sex_female_country_USA__Grant_Co._...:0.0147036732)53:0.1682629435,(ref_NP_001006361.1__chromosome_2_organism_Gallus_gallus_breed_Leghorn_gcode_1_chromosome_2_map_2_gasdermin-E_Gallus_gallus:0.2690203959,ref_XP_009324004.1__chromosome_Unknown_completeness_complete_organism_Pygoscelis_adeliae_isolate_BGI_AS28_gcode_1_sex_male_country_Antarctica_chromosome_Unknown_PREDICTED__non-syndromic_hearing_impae:0.0452115560)100:0.1871772960)58:0.3897829869,ref_XP_029444983.1__location_chromosome_chromosome_2_completeness_complete_organism_Rhinatrema_bivittatum_gcode_1_chromosome_2_gasdermin-E_isoform_X1_Rhinatrema_bivittatum:0.3664004251)60:0.2861438592,(ref_XP_002933445.2__location_chromosome_chromosome_6_completeness_complete_organism_Xenopus_tropicalis_strain_Nigerian_gcode_1_chromosome_6_sex_female_tissue_type_liver_and_blood_dev_stage_adult_nots:0.7466063413,ref_XP_040208817.1__location_chromosome_chromosome_5_completeness_complete_organism_Rana_temporaria_gcode_1_chromosome_5_gasdermin-E_Rana_temporaria:0.5253528572)99:0.2166526727)100:1.1574562036,XP_032891479.1_gasdermin-A_Amblyraja_radiata:0.9838009380)99:0.7098439536,ref_XP_015154998.2__location_chromosome_chromosome_27_completeness_complete_organism_Gallus_gallus_isolate_bGalGal1_gcode_1_chromosome_27_sex_female_tissue_type_blood_country_USA__Fayetteville_lat_ls:0.9735095635)92:0.0659559447,ref_XP_029428328.1__location_chromosome_chromosome_12_completeness_complete_organism_Rhinatrema_bivittatum_gcode_1_chromosome_12_gasdermin-A_Rhinatrema_bivittatum:1.3128880614)95:0.2653475672,((((((ref_XP_035935879.1__chromosome_Unknown_completeness_complete_organism_Halichoerus_grypus_isolate_241Hg_ecotype_Northwest_Atlantic_gcode_1_chromosome_Unknown_sex_male_tissue_type_blood_dev_stage_molts:0.0807691174,ref_XP_004633937.1__chromosome_Unknown_completeness_complete_organism_Octodon_degus_isolate_3935_gcode_1_chromosome_Unknown_sex_female_gasdermin-A_Octodon_degus:0.0913688971)77:0.0155918263,(((ref_XP_014975037.1__location_chromosome_chromosome_16_completeness_complete_organism_Macaca_mulatta_isolate_AG07107_bio_material_Coriell_AG07107_gcode_1_chromosome_16_sex_female_tissue_type_fibroblaa:0.0436633563,ref_NP_835465.2__chromosome_17_organism_Homo_sapiens_gcode_1_chromosome_17_map_17q21.1_gasdermin-A_Homo_sapiens:0.0000029581)96:0.0108587622,ref_XP_001171222.1__location_chromosome_chromosome_17_completeness_complete_organism_Pan_troglodytes_isolate_Yerkes_chimp_pedigree__C0471__Clint__gcode_1_chromosome_17_sex_male_tissue_type_blood_devs:0.0000029607)100:0.0491106952,ref_XP_024429158.1__chromosome_Unknown_completeness_complete_organism_Desmodus_rotundus_isolate_DRU21DN04_gcode_1_chromosome_Unknown_sex_male_tissue_type_muscle_skin_sample_dev_stage_adult_country_Us:0.0736267252)91:0.0110020515)66:0.0000029651,(ref_XP_016020091.2__chromosome_Unknown_completeness_complete_organism_Rousettus_aegyptiacus_isolate_mRouAeg1_gcode_1_chromosome_Unknown_sex_male_tissue_type_muscle_dev_stage_adult_country_USA__Berkes:0.0130945158,ref_XP_006924750.1__chromosome_Unknown_completeness_complete_organism_Pteropus_alecto_gcode_1_chromosome_Unknown_sex_male_tissue_type_kidney_country_Australia__Anglican_Church_Grammar_School_East_Bro:0.0155295525)99:0.0480278269)72:0.0149589716,ref_XP_024836266.1__location_chromosome_chromosome_19_completeness_complete_organism_Bos_taurus_isolate_L1_Dominette_01449_registration_number_42190680_breed_Hereford_gcode_1_chromosome_19_sex_fem...:0.0718989089)91:0.0530564322,sp_Q9EST1_GSDMA_MOUSE_Gasdermin-A_OS_Mus_musculus_OX_10090_GN_Gsdma_PE_2_SV_1:0.0166635663)90:0.0490339345,(sp_Q32M21_GSDA2_MOUSE_Gasdermin-A2_OS_Mus_musculus_OX_10090_GN_Gsdma2_PE_2_SV_1:0.1119388985,sp_Q5Y4Y6_GSDA3_MOUSE_Gasdermin-A3_OS_Mus_musculus_OX_10090_GN_Gsdma3_PE_1_SV_1:0.0293760062)51:0.0124508742)100:0.5315687744)55:0.0398466008,(((ref_NP_081236.1__chromosome_15_organism_Mus_musculus_strain_C57BL_6_gcode_1_chromosome_15_map_15_gasdermin-D_Mus_musculus:0.4296195654,(ref_XP_015001615.2__location_chromosome_chromosome_8_completeness_complete_organism_Macaca_mulatta_isolate_AG07107_bio_material_Coriell_AG07107_gcode_1_chromosome_8_sex_female_tissue_type_fibroblasta:0.0295894078,(ref_XP_009454389.2__location_chromosome_chromosome_8_completeness_complete_organism_Pan_troglodytes_isolate_Yerkes_chimp_pedigree__C0471__Clint__gcode_1_chromosome_8_sex_male_tissue_type_blood_dev_ss:0.0314150535,ref_NP_079012.3__chromosome_8_organism_Homo_sapiens_gcode_1_chromosome_8_map_8q24.3_gasdermin-D_Homo_sapiens:0.0000029607)100:0.0231882635)100:0.2440102957)96:0.1337700359,(ref_XP_023104116.1__location_chromosome_chromosome_F2_completeness_complete_organism_Felis_catus_isolate_Cinnamon_breed_Abyssinian_gcode_1_chromosome_F2_sex_female_gasdermin-D_Felis_catus:0.3917435594,(ref_NP_001039625.1__chromosome_14_organism_Bos_taurus_breed_Hereford_gcode_1_chromosome_14_map_14_gasdermin-D_Bos_taurus:0.1894642594,(ref_XP_024428237.1__chromosome_Unknown_completeness_complete_organism_Desmodus_rotundus_isolate_DRU21DN04_gcode_1_chromosome_Unknown_sex_male_tissue_type_muscle_skin_sample_dev_stage_adult_country_Us:0.2362370467,(ref_XP_006913014.1__chromosome_Unknown_completeness_complete_organism_Pteropus_alecto_gcode_1_chromosome_Unknown_sex_male_tissue_type_kidney_country_Australia__Anglican_Church_Grammar_School_East_Bro:0.0651402090,ref_XP_036089037.1__chromosome_Unknown_completeness_complete_organism_Rousettus_aegyptiacus_isolate_mRouAeg1_gcode_1_chromosome_Unknown_sex_male_tissue_type_muscle_dev_stage_adult_country_USA__Berkes:0.0758158329)100:0.0833051999)93:0.0881585966)96:0.1235663553)92:0.0772058967)100:0.6175507089,(ref_XP_023358160.1__location_chromosome_chromosome_4_completeness_complete_organism_Sarcophilus_harrisii_gcode_1_chromosome_4_cell_line_91H_gasdermin-B_Sarcophilus_harrisii:0.8633702800,((((ref_NP_001231146.1__chromosome_19_organism_Bos_taurus_gcode_1_chromosome_19_map_19_gasdermin-B_Bos_taurus:0.6587275547,ref_XP_035935913.1__chromosome_Unknown_completeness_complete_organism_Halichoerus_grypus_isolate_241Hg_ecotype_Northwest_Atlantic_gcode_1_chromosome_Unknown_sex_male_tissue_type_blood_dev_stage_molts:0.7722501048)96:0.1435701087,ref_XP_024429114.1__chromosome_Unknown_completeness_complete_organism_Desmodus_rotundus_isolate_DRU21DN04_gcode_1_chromosome_Unknown_sex_male_tissue_type_muscle_skin_sample_dev_stage_adult_country_Us:0.2731137987)98:0.1584538890,(ref_XP_024897515.1__chromosome_Unknown_completeness_complete_organism_Pteropus_alecto_gcode_1_chromosome_Unknown_sex_male_tissue_type_kidney_country_Australia__Anglican_Church_Grammar_School_East_Bro:0.0791893445,ref_XP_036078993.1__chromosome_Unknown_completeness_complete_organism_Rousettus_aegyptiacus_isolate_mRouAeg1_gcode_1_chromosome_Unknown_sex_male_tissue_type_muscle_dev_stage_adult_country_USA__Berkes:0.2377456346)86:0.0567080828)86:0.1882097164,(ref_XP_014975035.1__location_chromosome_chromosome_16_completeness_complete_organism_Macaca_mulatta_isolate_AG07107_bio_material_Coriell_AG07107_gcode_1_chromosome_16_sex_female_tissue_type_fibroblaa:0.2676638884,(ref_NP_001159430.1__chromosome_17_organism_Homo_sapiens_gcode_1_chromosome_17_map_17q21.1_gasdermin-B_isoform_3_Homo_sapiens:0.0290767859,ref_XP_009430521.1__location_chromosome_chromosome_17_completeness_complete_organism_Pan_troglodytes_isolate_Yerkes_chimp_pedigree__C0471__Clint__gcode_1_chromosome_17_sex_male_tissue_type_blood_devs:0.0178508959)100:0.0628526003)100:0.3192200912)100:0.8821326619)97:0.2538096270)85:0.2551233162)91:0.2078305605,ref_XP_031803207.1__location_chromosome_chromosome_1_completeness_complete_organism_Sarcophilus_harrisii_gcode_1_chromosome_1_cell_line_91H_gasdermin-C_Sarcophilus_harrisii:0.8416961765)79:0.2050744678,((ref_NP_001039469.1__chromosome_14_organism_Bos_taurus_breed_L1_Hereford_gcode_1_chromosome_14_map_14_gasdermin-C_Bos_taurus:0.4616807552,((ref_XP_021073018.1__location_chromosome_chromosome_17_completeness_complete_organism_Mus_pahari_gcode_1_chromosome_17_sex_female_tissue_type_tail_gasdermin-C_Mus_pahari:0.5497824926,(ref_XP_028708741.1__location_chromosome_chromosome_8_completeness_complete_organism_Macaca_mulatta_isolate_AG07107_bio_material_Coriell_AG07107_gcode_1_chromosome_8_sex_female_tissue_type_fibroblasta:0.0465299352,(ref_NP_113603.1__chromosome_8_organism_Homo_sapiens_gcode_1_chromosome_8_map_8q24.21_gasdermin-C_Homo_sapiens:0.0061527148,ref_XP_001153860.1__location_chromosome_chromosome_8_completeness_complete_organism_Pan_troglodytes_isolate_Yerkes_chimp_pedigree__C0471__Clint__gcode_1_chromosome_8_sex_male_tissue_type_blood_dev_ss:0.0420937011)100:0.0296370828)100:0.1854451232)71:0.0493527295,(ref_XP_036088919.1__chromosome_Unknown_completeness_complete_organism_Rousettus_aegyptiacus_isolate_mRouAeg1_gcode_1_chromosome_Unknown_sex_male_tissue_type_muscle_dev_stage_adult_country_USA__Berkes:0.3503405224,ref_XP_024427836.1__chromosome_Unknown_completeness_complete_organism_Desmodus_rotundus_isolate_DRU21DN04_gcode_1_chromosome_Unknown_sex_male_tissue_type_muscle_skin_sample_dev_stage_adult_country_Us:0.2126263019)96:0.0851915729)71:0.1411697281)91:0.1778018319,ref_XP_019678683.1__location_chromosome_chromosome_F2_completeness_complete_organism_Felis_catus_isolate_Cinnamon_breed_Abyssinian_gcode_1_chromosome_F2_sex_female_gasdermin-C_Felis_catus:0.3701279637)98:0.4342141779)96:0.8962896994)54:0.3395672531)99:1.2506313176)94:0.3587345866);

PYD Tree

(sp_Q8WXC3_PYDC1_HUMAN:0.0000021986,NP_660183.1_1-88:0.8189957255,((((NP_001104231.1:0.5582152076,(q919n6_danio:0.1596274241,q919l7_caspa:0.1464068014)100:1.6604787690)78:0.3649286491,q504j1_caspb:0.8779213839)75:0.3248494404,q91vj1_pycard:1.3275505794)53:0.3488003785,(q56p42_pydc2:2.4248812488,((((o35368_ifi203:1.9252008714,(tr_B2CWB9_B2CWB9_9POXV:0.1873719086,NP_051902.1:0.1151924580)98:0.8588394249)30:0.1685723872,QHG62576.1_hypothetical:1.3381578798)25:0.0205813423,((YP_227401.1:0.4922697513,((NP_073403.1:0.0000024848,ABQ43490.1_pyrin:0.0322639322)100:0.5536693021,NP_570174.1:0.6631629338)89:0.1050133382)91:0.2980680604,(QDJ94995.1_IFN-inducible:0.0322861746,YP_009407969.1:0.3451439803)100:1.1424169120)56:0.1652826846)38:0.2909839033,((((((Vaccinia:0.0000024478,(Cowpox:0.0000024085,((Orthopox_abatino:0.1055672911,Alaskapox:0.0953186299)96:0.0427250561,Akhmeta_virus:0.0648530371)92:0.0274133695)92:0.0511067176)53:0.0098506539,Buffalopox:0.0000023745)51:0.0083698252,(Rabbitpox:0.0000027502,((Horsepox:0.0531890168,Variola_Virus:0.0358970206)28:0.0000020161,Monkeypox:0.0242749897)26:0.0000027502)32:0.0115162760)97:0.2153206803,(Volepox:0.0403124363,Skunkpox:0.0547150846)81:0.0431585044)36:0.0492731175,Raccoonpox:0.0848552751)84:0.2158394416,((NY_014:0.0278797725,Murmansk_poxvirus:0.0311930000)97:0.1567232511,Yoka_poxvirus:0.3046051547)97:0.3533709024)96:0.7863255573)26:0.1931065783)79:0.4863747229)100:0.9712551055);

DNA Polymerase Tree

(MT712273.1_55688-58654:2.5584313207,(NC_028238.1_70062-73028:1.9228613427,NC_006998.1_c56656-53636:9.6541110156)90:0.6360366954,((((OK348853.1_91721-94687:0.0000029233,NC_002188.1_93460-96426:0.0000029233)100:0.2890607953,(NC_036582.1_95291-98260:0.0029595319,OP292971.1_108285-111254:0.0086872138)81:0.0125534112)77:0.0796120677,(NC_024447.1_100701-103667:0.0572080844,NC_024446.1_101802-104768:0.0381255670)98:0.0541196830)100:1.2223071060,((MT799800.1_116606-119572:0.6598637406,(OM869483.1_115192-118158:0.0357538333,((MK903864.1_103203-106169:0.0438871461,ON408417.1_116303-119269:0.0272873441)63:0.0165220543,NC_005309.1_122909-125875:0.0140937333)56:0.0294472186)100:0.5769287496)71:0.3206755027,KX857216.1_108448-111414:0.4506249724)100:1.4588372318)69:0.5441896556);
